# Identification of lipid quantitative trait loci linked with cardiometabolic disease in Asian Indians and Europeans: A genome-wide association study and Mendelian randomization

**DOI:** 10.1371/journal.pmed.1005039

**Published:** 2026-04-23

**Authors:** Madhusmita Rout, Christopher E. Aston, Ravindranath Duggirala, Harald H. Goring, Oliver Fiehn, Dharambir K. Sanghera

**Affiliations:** 1 Department of Pediatrics, College of Medicine, University of Oklahoma Health Sciences Center, Oklahoma City, Oklahoma, United States of America; 2 Harold Hamm Diabetes Center, University of Oklahoma Health Sciences Center, Oklahoma City, Oklahoma, United States of America; 3 Department of Health and Behavioural Sciences, University of Texas A&M, San Antonio, Texas, United States of America; 4 South Texas Diabetes and Obesity Institute, The University of Texas Rio Grande Valley, Edinburg, Texas, United States of America; 5 UC Davis West Coast Metabolomics Center, Davis, California, United States of America; 6 Department of Pharmaceutical Sciences, University of Oklahoma Health Sciences Center, Oklahoma City, Oklahoma, United States of America; 7 Department of Physiology, College of Medicine, University of Oklahoma Health Sciences Center, Oklahoma City, Oklahoma, United States of America; 8 Oklahoma Center for Neuroscience, University of Oklahoma Health Sciences Center, Oklahoma City, Oklahoma, United States of America; Shanghai Jiao Tong University Affiliated Sixth People's Hospital, CHINA

## Abstract

**Background:**

Genetic mechanisms that predispose people to type 2 diabetes (T2D) and cardiovascular disease (CVD) remain poorly understood, partly because of a lack of sufficient data on non-European ethnic groups. Extending these evaluations to diverse cohorts is essential for gaining insights into the molecular pathways involved in disease development among human populations. In this study, we aimed to evaluate the genetic connection between the human lipidome and cardiometabolic disorders. We conducted a metabolite genome-wide association study (mGWAS) in a Punjabi population from India, along with multi-layer replication studies using the UK Biobank and other independent European and non-European cohorts.

**Methods and findings:**

We performed mGWAS using 516 lipid metabolites in 3,000 Punjabi Sikh individuals, and validation was performed in 1.13M Europeans and 15K individuals from Asian Indian ancestry using independent cohorts of the UK Biobank, GeneRISK, DIAMANT, PROMIS, and other studies. We identified 609 SNP-metabolite associations representing 236 SNP-metabolite pairs that attained genome-wide significance (*p* </= 5 × 10^−8^). Of the 36 SNP-lipid metabolite signals that survived multiple testing correction (*p* </= 1.92 × 10^−10^), 33 associations were not reported before, and 3 associations were confirmed to be ancestry-specific. Using colocalization analysis, polygenic risk scores, and Mendelian randomization approaches, we identified a causal association of LPC O-16:0 with T2D, represented by a lead variant in CD45, a key regulator of T- and B-cell antigen receptor signaling, and is already used as a therapeutic target. Another possible causal relationship of PC 38:4 (C) in protecting against coronary artery disease risk in Asian Indians, attributed to a variant in the untranslated region in the *FADS1/2* genes, may be specific to ancestry and/or could not be confirmed in Europeans because of extensive pleiotropy in this region. The main limitation of this study was the absence of an independent validation cohort of Asian Indians from India.

**Conclusions:**

The mGWAS of Asian Indians offers new insights into the diverse molecular origins of cardiometabolic diseases and suggests potential pathways for innovative treatments. Our findings highlight the need for additional research on human lipidomics to better understand the downstream effects of the genome and its impact on cardiometabolic health.

## Author summary

**Why was this study done?:**

- Genome-wide studies have shown that genes influencing blood lipid metabolites are often linked to different diseases.
- However, most of this research has been done in people of European ancestry.
- Studying more diverse populations is important to better understand how these genetic pathways contribute to disease in different ethnic groups.

**What did the researchers do and find?:**

- We analyzed genetic influences on 516 lipids in 3,000 Punjabi Sikh individuals and validated the results in large European and non-European cohorts.
- We identified new genetic pathways linking to specific lipids and confirmed the causal association of LPC O-16:0 with T2D influenced by genetic variation in genes regulating T- and B-cell antigen receptor signaling.
- The rigorous multilayered analysis and validation studies also identified an ancestry-specific variant in genes that may protect against heart disease risk by modulating PC 38:4 in Asian Indians.

**What do these findings mean?:**

- Through rigorous analytical approaches, our study has discovered new metabolite markers and genes that intersect with pathways of inflammation and immuno-vascular diseases, which have not been reported in previous European studies.
- Including more non-European cohorts in such research would ultimately aid in identifying distinct disease subtypes based on genetic pathways. These advances could be beneficial in clinical practice, enabling the effective personalized therapies and preventive strategies.
- The absence of an independent validation in a similar ethnic cohort due to the unavailability of data is the main limitation of this study.

## Abstract 3

In a genome-wide association study combined with Mendelian randomization, Madhusmita Rout and colleagues investigated associations between the human lipidome and cardiometabolic disorders in an Indian population.

## Introduction

The increasing prevalence of type 2 diabetes (T2D), obesity, and cardiovascular disease (CVD) represents a significant public health crisis of the 21st century. According to the International Diabetes Federation’s projections, diabetes prevalence globally will rise from 463 million in 2019–700 million by 2045. Numerous epidemiological and clinical studies on South Asians (SAs) living abroad have consistently demonstrated that, compared to Caucasians and other ethnic groups, SAs have a higher incidence of T2D and are significantly more susceptible to CVD [1]. Rapid urbanization, population growth, western diets, pollution, and aging have made the Indian subcontinent an epicenter of the growing epidemic of cardiometabolic diseases [2,3]. SAs exhibit a unique body composition with uneven adiposity marked by a low muscle mass, higher fat around the abdomen, with a low body mass index (BMI) but a metabolically obese phenotype as highlighted by multiple independent studies, which may correlate with the increased chronic low-grade inflammation, insulin resistance, T2D, and a high prevalence of macro and microvascular complications in this population [1,4,5]. However, the exact mechanism responsible for their increased risk of diabetes and CV diseases is unknown. Individuals from the Indian subcontinent are estimated to make up 60% of the global burden of CVD [6]. However, there is a lack of comprehensive lipidomic and genome-wide data for Asian Indian populations.

Circulating lipids and lipid subclasses, such as free fatty acids (FA), ceramides (Cer), sphingolipids (SM), phospholipids, and triacylglycerols (TGs), are shown to be differentially regulated in T2D, heart disease, stroke, Alzheimer’s, and cancer in multiple investigations, including studies from our group [7–14]. Genome-wide association studies (GWAS) of blood lipids have identified genetic associations shared between lipid subclasses and various diseases; however, the large bulk of the information from such studies is derived from European (EU) populations. Therefore, extending these evaluations to diverse cohorts would be crucial for gaining insights into the molecular pathways of disease development among diverse ethnic groups.

In this study, we have performed a metabolite-GWAS of blood lipid phenotypes associated with T2D and CV traits in a Punjabi population originating from Northern India. The overall goals of this study were to identify new genetic associations with lipid metabolites, validate known metabolite-genetic associations, and identify ancestry-specific differences using 468,515 individuals from the UK Biobank (UKBB), including British EU (459,143) and SA (9,372), and other independent cohorts from published studies. Further, using fine mapping, colocalization, and Mendelian randomization (MR) approaches, we aimed to identify candidate causal variants showing shared genetic associations between metabolites and cardiometabolic disease phenotypes.

## Materials and methods

### Study cohort

A total of 3,000 individuals (1,725 T2D cases and 1,275 controls) were included in this study from the Asian Indian Diabetic Heart Study (AIDHS)/Sikh Diabetes Study (SDS) [15–17]. The Sikh population is a relatively homogenous endogamous community from Northern India. Sikhs are primarily non-smokers, and ~50% of them are vegetarians. However, the incidence of cardiometabolic diseases in Sikhs and SAs has markedly increased over the past two decades [1,18]. T2D was diagnosed based on their medical records for symptoms and use of diabetic medications, and following the American Diabetes Association guidelines as described earlier [16,17]. Non-diabetic controls (*N* = 1,275) were selected based on a fasting blood glucose (FBG) <100.8 mg/dl (5.6 mmol/l) or a 2-hour glucose <141.0 mg/dl (7.8 mmol/l). All blood samples were obtained at the baseline visit. Subjects with type 1 diabetes, those with a family member with type 1 diabetes, or rare forms of T2D subtypes (maturity-onset diabetes of the young [MODY]) or secondary diabetes (from, e.g., hemochromatosis or pancreatitis) were excluded from the study based on clinical reports, as previously described [15,17,19]. Coronary artery disease (CAD) was considered if there was use of nitrate medication (nitroglycerine), electrocardiographic evidence of angina pain, coronary angiographic evidence of severe (greater than 50%) stenosis, or echocardiographic evidence of myocardial infarction. The diagnosis was based on the date of coronary artery bypass graft (CABG) or angioplasty and medication usage obtained from patient records, as described previously [17,20]. All participants in this study were recruited following the written informed consent procedures approved by the institutional review boards (IRBs). All AIDHS/SDS protocols and consent documents were reviewed and approved by the University of Oklahoma Health Science Center’s (IRB #2911 and IRB # 13302), the University of Pittsburgh (IRB # 021234), as well as the Human Subject Protection (Ethics) Committees at the participating hospitals and institutes in India, as described previously [14,21–23]. The local institutions and hospitals also separately obtained Federal Wide Assurance (FWA) from the Office of Human Research Protection, US Department of Health and Human Services (S1 File). All human studies reported in this manuscript abided by the Declaration of Helsinki principles.

**Fig 1:**
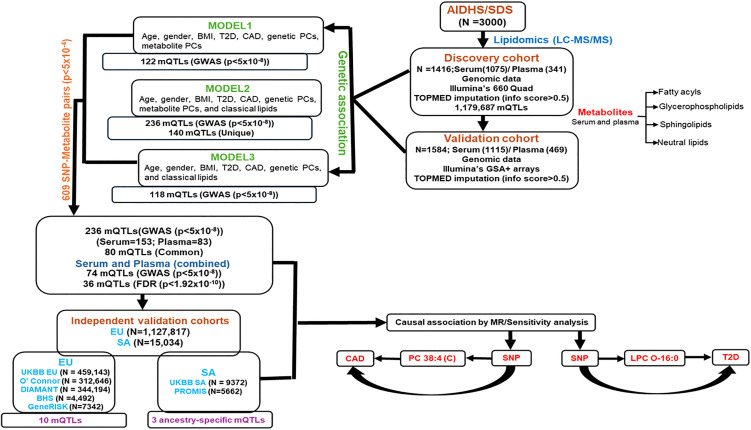
Summary of the workflow detailing the study design, lipidomics analysis, and the outcomes.

BMI was calculated as [weight (kg)/height (m^2^)]. A tape measure of the waist and hip circumferences at the abdomen and the hip, respectively, were recorded. For using BMI thresholds for obesity, we used the World Health Organization’s (WHO) guidelines [24]. Blood pressure (BP) was measured twice after a 5-minute seated rest period with the participant’s feet flat on the floor. Serum lipids [total cholesterol (TC), TGs, high‐density lipoprotein cholesterol (HDL‐C), and low‐density lipoprotein cholesterol (LDL‐C)] were measured using standard enzymatic methods (Roche, Basel, Switzerland) as described previously [17,20,25–27]. The design and the workflow for the study are shown in Fig 1.

### Inclusivity in global research

Additional information regarding the ethical, cultural, and scientific considerations specific to inclusivity in global research is included in the Supporting Information (S1 Text).

### Metabolomics/lipidomics profiling, quality control (QC), and analysis

Aliquots of 50 µL serum/plasma were shipped on dry ice to the UC Davis West Coast Metabolomics Center to measure lipidomics profiles using liquid chromatography–tandem mass spectrometry (LC–MS/MS) in untargeted mode. The serum and plasma aliquots were extracted with a degassed ternary mixture of methanol, MTBE, and water, as described in detail previously [28]. The data produced by LC–MS/MS is a relative quantification method that utilizes internal standards as surrogate markers for quantification. The LC–MS/MS analyses were conducted using an Agilent 1290 Infinity LC system (Agilent Technologies, Santa Clara, CA, USA) equipped with a Waters Acquity CSH C18 2.1 × 100 mm, 1.7 μm particle size column, maintained at 65 °C at a flow rate of 0.6 mL/min. The mobile phases consisted of (A) 60:40 (v/v) acetonitrile:water with 10 mM ammonium formate/0.1% formic acid and (B) 90:10 (v/v) isopropanol:acetonitrile with 10 mM ammonium formate/0.1% formic acid. Chromatography gradient was: 0 min 15% (B); 0–2 min 30% (B); 2–2.5 min 48% (B); 2.5–11 min 82% (B); 11–11.5 min 99% (B); 11.5–12 min 99% (B); 12–12.1 min 15% (B); and 12.1–15 min 15% (B). Mass spectrometric detection of lipids was performed on Agilent 6530 and 6546 QTOF mass spectrometers [28] in data-dependent MS/MS mode using both electrospray ionization positive and negative ion modes. Raw data were processed using MS-DIAL (v. 4.90) software [29] from 280 to 1,500 Da from 0.3 to 12.6 min with a centroiding tolerance of 10 mDa and a minimum peak height amplitude of 500 with a mass slice width of 50 mDa, an accurate mass tolerance of 10 mDa, and 6s alignment retention time tolerance as described [29].

We used three types of internal standards for the analysis: 43 Biorec (commercial), 43 blank, and 43 pooled samples (derived from representative samples of the AIDHS/SDS population) to assess the technical variance and robustness of the analytical method, as described previously [30,31]. Lipids were identified by MS/MS matching to LipidBlast within MS-DIAL at MS1 difference <10 mDa and MS/MS similarity >700 with manual inspection of all similarity matches. All peaks had signal-to-noise ratios greater than 3:1 for annotated compounds and greater than 10:1 for unknowns, using blank samples as negative controls. All data peaks were processed using MS-DIAL 4 and the concentrations (pmol per μl serum or plasma) were calculated by using internal standards, where the tag of level 2 was assigned if the lipid was quantified by an internal standard of the same lipid polar head class, and the tag of level 3 was assigned if the lipid was quantified by an internal standard of a similar lipid class or representative standard compound based on the Lipid Standard Initiative (LSI) guidelines (https://lipidomics-standards-initiative.org/) as described previously [29]. The identification of unknown MS/MS spectra was elucidated by using internal standards, consulting the literature, or predicting the putative structure based on fragment ion evidence.

### Batch correction

The samples were sent in two batches, each containing 1,500 samples, to measure the lipidomics profile. Each batch was sent separately at a different time, with a gap of approximately a year between processing. QC was assured by randomizing injection sequences and injecting 10 QC pool samples before the actual sequence of samples, injecting QC pool samples at the beginning and the end of each batch sequence and between each 10 actual samples, injecting QC method blanks after each set of 10 actual samples, checking the peak shape and the intensity of spiked internal standards and the internal standard added before injection. The QC pool samples were used to correct for batch differences, longitudinal drift, and instrument variation, causing technical data variance by random forest machine learning in the SERRF algorithm [32]. SERRF defines systematic errors as errors not only associated with batch effects and injection order but also considers patterns of related compounds. The cross-validated relative standard deviation (coefficient of variation, %CV) is used to evaluate the data performance. Per batch, reproducibility was achieved with a 7.3% CV as the median overall acquired lipids in positive electrospray ionization (ESI) mode using the sample pool QCs. For the negative ESI mode, reproducibility was measured at a median 8.6% CV across all lipids for the sample pool QCs.

### Genotyping, imputation, and quality controls

Genomic DNA was extracted from buffy coats using QIAamp blood kits (Qiagen, Chatsworth, CA) or by the salting-out procedure [33]. Samples were genotyped using the Illumina 660W Quad BeadChip, Illumina Global Screening Arrays (GSA), and GSA with multi-disease content (GSA+) arrays as described previously [19,20,34]. Both discovery (training) and test sets for Punjabi cohorts originated from the AIDHS/SDS, excluding families due to relatedness. The discovery set was genotyped using Illumina’s 660 Quad and the training cohort with GSA+ arrays. There was no sample overlap in the training and test datasets. Also, samples with genotyping call rate <95%, cryptic relatedness, population outliers, departures from Hardy–Weinberg equilibrium (HWE) (*p* < 10^−7^) or minor allele frequency (MAF) <5% were excluded before association testing. To increase genome coverage, data were imputed using Minimac4 [35] (https://imputationserver.sph.umich.edu/) with TOPMED r3 multiethnic reference panel in NCBI Build 38 (hg38) coordinates as reported previously [3,36]. Of a total of 23,739,260 variants, we removed variants with an imputation certainty info score <0.5, MAF < 0.001, and with HWE in controls (*p* < 1 × 10^−6^) before further analysis. The genetic principal components (PCs) were estimated from our Sikh population, as the existing HapMap2, HapMap3, and 1000 Genomes data do not include data from Punjabi Sikhs, as described previously [17,34]. The lipid metabolite PCs were calculated from the metabolite data generated for the 3,000 AIDHS/SDS individuals for serum and plasma, respectively, using a standard PCA algorithm, which is an orthogonal transformation that captures the maximum variance in the data. This process involves centring the data, calculating the covariance matrix, and then finding the eigenvectors and eigenvalues of that matrix [13,37].

### Statistical analysis

The clinical and demographic variables were summarized using the mean for continuous variables and percentages for categorical variables using the SPSS software version 29 (IBM, New York City, USA). Multivariate linear regression analyses were performed to assess the impact of individual metabolite markers on T2D, CAD, and other cardiometabolic risk factors (e.g., BMI, waist, FBG) after adjusting for covariates such as age, sex, and medications as described previously [13]. We conducted a lipid metabolite GWAS on 385 serum and 321 plasma metabolite species that are associated with T2D and other cardiometabolic traits. Genome-wide analysis was performed after adjusting for age, sex, BMI, T2D, CAD, 5 genetic PCs, and 5 metabolite PCs in Model 1 and including blood lipids in Model 2, along with covariates of age, sex, BMI, T2D, CAD, 5 genetic, and metabolite PCs [38]. We further performed association analysis for top mQTLs after adjusting for age, sex, BMI, T2D, CAD, 5 genetic PCs, and blood lipids in Model 3. For selecting the independent signals, LD clumping was used with an LD threshold (*R*^2^ > 0.6). All regression analyses were performed using PLINK 2.0 [39]. We assessed the pairwise correlation between selected lipids and phenotypic traits such as blood glucose, TG, systolic blood pressure (SBP), and diastolic blood pressure (DBP) using SPSS software version 29 (IBM, New York City, USA). Logistic regression analyses were performed to assess the impact of metabolite-associated SNPs on T2D and CAD after adjusting for covariates using SVS version 8.9.1 (Golden Helix, Bozeman, MT, USA). The heritability of each lipid metabolite was calculated using the genome-wide complex trait analysis (GCTA) software package [40] and selecting genome-wide significant variants. All analyses were performed using PLINK 2.0 [39], SVS version 8.9.1 (Golden Helix, Bozeman, MT, USA), the SPSS software version 29 (IBM, New York City, USA), and Metaboanalyst 6.0 (Quebec, Canada).

### Independent validation cohorts

#### Replication studies for the genetic association of metabolites.

The genetic associations in the AIDHS/SDS were replicated using the summary statistics data in multiple studies available with genome-wide genotypes and lipidomics data. For SAs, we utilized the summary statistics data of 340 known lipids from the 5,662 participants in a study from the Pakistani cohort [41]. We used summary statistics data of 596 lipid species in serum from 4,492 individuals from the Busselton Health Study (BHS) [42]. The BHS is a community-based study in Western Australia that includes both related and unrelated individuals (predominantly of EU ancestry), containing genome-wide SNP data, longitudinal phenotype data, and blood serum data. We used summary statistics data on 179 lipid species from 7,342 individuals in the prospective GeneRISK cohort [43,44], which assesses the impact of the genetic risk of CVD in people from Southern Finland [45].

Additionally, we used genome-wide genotype and phenotype data from the UKBB in individuals of EU (*N* = 459,143) and SA ancestry (*N* = 9,372) to replicate metabolite-associated SNPs with T2D and CAD following the approval (Application # 78635) described previously [3,36]. For genetic analysis, we used imputed data released by the UKBB for EU and SA subjects and excluded outliers for heterozygosity or genotype missing rates (0.2 > missing rate) as well as ambiguous SNPs (MAF > 0.44). Participants with inconsistent reports and genotypic inferred sex inconsistencies or withdrawn consent were removed, as explained previously [46].

Additionally, we used the summary statistics data from O’Connor and colleagues [47], which comprised datasets from seven EU cohorts (*N* = 312,646) containing 33,122,978 variants, and available for both additive and recessive models to understand the association of T2D with metabolite-associated SNPs. We also utilized summary statistics data from the DIAMANT cohort (*N* = 344,194) [48], a population-based, dynamic, and prospective cohort of individuals with diabetes, to understand the association between T2D and metabolite-associated SNPs.

### Genome-wide polygenic risk score (PRS) construction and analysis

Asian Indian ancestry-specific PRS (PRSAI) for T2D and CAD were constructed using candidate variants derived from genome-wide genotypes of AIDHS/SDS and other robustly associated variants reported for GWAS studies of CAD and T2D for people from SA studies [17,20,34,49,50]. The details of SNP selection criteria from 46,985,978 common and rare variants for the PRS construction have been described previously [3,36]. After regression analysis (1) we selected all significant SNPs (*p* < 10^−2^), (2) QC was performed (a) excluding INDELs, duplicate and multiallelic SNPs, (b) SNPs with info score <0.80, (c) including SNPs with MAF > 0.01 and MAF < 0.45, (3) Remaining SNPs with *p* < 10^−4^ were chosen. (4) Then we removed clumping-based linkage disequilibrium (LD) using *R*^2^ (LD) <= 0.25 and 500 Kb distance, a total of 2,921 and 925 significant SNPs were used for the construction of the PRSAI in T2D and CAD, respectively [3,36,51]. To construct European PRS (PRSEU), we used the summary statistics data from O’Connor *and colleagues*, [47] for T2D PRS and 113,320 variants from the Myocardial Infarction Genetics and CARDIoGRAM Exome chip meta-analysis [52]. The selection criteria of SNPs for constructing European PRSEU were the same as PRSAI. A total of 1847 and 405 significant SNPs were selected for the construction of the PRSEU in T2D and CAD, respectively. The individual-level regression coefficients were multiplied by the number of risk alleles to compute the PRS in training and test sets as described previously [3,36,53]. The weighted PRS was calculated using the following equation:


$${PRS}_{j}=\sum_{i}^{N}\;\beta_{i}*\;{dosage}_{ij}\;$$

where *N* is the number of SNPs in the score, *β*_*i*_ is the effect size (or beta) of variant *i*, and dosage is the number of copies of SNP in the genotype of individual *j* [54].

For exploratory analysis, we also constructed the genome-wide PRS for selected top lipid species, which showed a strong causal association with cardiometabolic traits using 2,577 and 2,538 SNPs, respectively, for LPC O-16:0 and PC 38:4 (C), using the same QC details described above for T2D and CAD PRS. The goal was to compare the genetic contribution of a single lead variant related to lipid traits with that of combined polygenic variants concerning the disease.

### MR analysis

We performed a two-sample MR analysis [55] to investigate the causal effect of metabolite-associated SNPs on T2D and CAD. The associations between the instrumental variables (gene variants) and the exposure (metabolite) and the outcome (T2D, CAD, or risk factor phenotypes) are estimated from different studies, mainly AIDHS/SDS, SA (UKBB)), EU (UKBB), data from O Connor and colleagues [47], and DIAMANT consortium (mixed ancestries). Three basic hypotheses were considered while conducting two-sample MR: (1) genetic instrument variables (IVs) should be robustly associated with the exposure; (2) IVs should not be directly correlated to the outcome and affect the outcome merely via the exposure without any gene pleiotropy; and (3) IV should be independent of any potential confounders. The combined SNP-specific estimates were calculated using the inverse-variance weighted (IVW) method when more than 2 associated SNPs were used as IVs. The odds ratios (ORs) of T2D and CAD were calculated per 1-SD increase in genetically predicted metabolite levels. Sensitivity analyses were performed using the MR Egger method of Burgess and Thompson [56], which is based on the hypothesis that the pleiotropic effects are independently distributed from the genetic associations with the exposure. A non-zero intercept is meaningful to MR Egger, signifying that gene pleiotropy is considered to exist. MR analyses were performed using the Two-sample MR package [57] in R version 4.3.3. We ensured that the genetic instrument was strongly associated with the exposure in the target (ancestry similar or non-similar) population based on regression (beta) coefficients and *p* value/*F* statistics accounting for the LD and allele frequency. Based on the differences in LD and MAF, the MR sensitivity analysis selected and excluded the variants from each ancestry to ensure data harmonization and reduce pleiotropy.

### Colocalization and pathway analyses

We further investigated whether a given genetic association of exposure (metabolite) was causally influencing the disease or trait outcomes by performing colocalization analysis using FUMA [58]. We analyzed each genetic region (maximum *p* value of lead SNPs < 10^−8^ and *R*^2^ threshold for independent SNPs < 0.5) for each associated QTL of interest by expanding 5Kb on each 5′ and 3′ site to explore if the genetic associations of exposure and the disease share the same underlying variants in a specific region. This analysis validates the causal association determined by MR and helps narrow down the potential causal genes. We also investigated whether the genetic association with the exposure or the disease is influenced by gene expression QTLs (eQTLs) in a particular tissue using FUMA. We identified chromatin interaction between eQTLs and disease or metabolite QTLs (mQTLs) using gene set enrichment analysis (GSEA). To gain insight into the underlying biological mechanisms of the identified set of significant mQTLs and disease associations, we performed over-representation analysis (ORA) implemented in integrated molecular pathway level analysis (IMPaLA) software [59] and using the data sources from Reactome, Kyoto Encyclopaedia of genes and genomes (KEGG), Encyclopaedia of human genes and metabolism (HumanCyc), and Wiki pathways as described previously [60].

### Fine mapping

Fine mapping analysis of the selected mQTLs were performed using a Bayesian fine-mapping method implemented in SuSiE using the GWAS summary statistics [61]. The sum of single effects model or SuSiE uses the sparse vector of regression coefficients as a sum of single-effect vectors, each with one non-zero element, and employs an iterative Bayesian stepwise selection (IBSS) that, instead of selecting a single variable at each step, computes a distribution on variables that captures uncertainty in which variable to select. IBSS thus helps in optimizing a variational approximation to the posterior distribution or posterior inclusion probability (PIP). The PIP is a value of each variable’s probability to be included in the true model in Bayesian analysis providing a credible set of variables for each selection [61].

## Results

**Fig 2:**
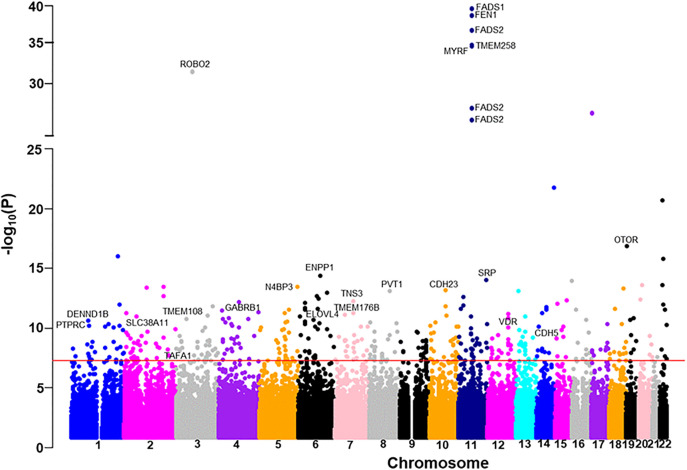
Manhattan plot showing associations of variants with serum lipid metabolite species of the AIDHS/SDS discovery cohort using directly genotyped and imputed SNPs on the x-axis and −log10 *p* value on the y-axis. The red horizontal line represents the genome-wide significance threshold of *p*-value = 5.0 × 10^−8^. The *p*-value was calculated using multivariate regression.

**Fig 3:**
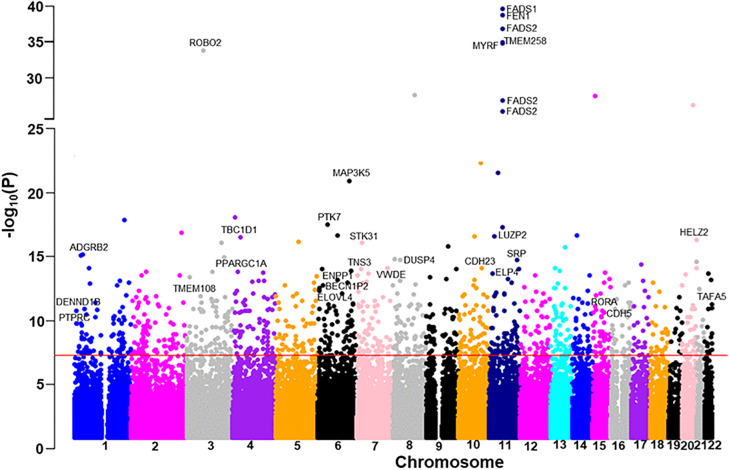
Manhattan plot showing associations of variants with plasma lipid metabolite species of the AIDHS/SDS discovery cohort using directly genotyped and imputed SNPs on the x-axis and −log10 *p* value on the y-axis. The red horizontal line represents the genome-wide significance threshold of *p*-value = 5.0 × 10^−8^. The *p*-value was calculated using multivariate regression.

The clinical and demographical characteristics of the AIDHS/SDS (with lipidomics data and complete cohort) and UKBB study subjects are shown in S1a, S1b, and S1c Table respectively. The workflow for this study is shown in Fig 1. Our LC–MS/MS analysis detected 1,565 and 1,880 serum and plasma lipids, respectively. After performing multi-layered QC involving peak resolution and batch effects, we evaluated phenotypic association of these metabolites with cardiometabolic phenotypic traits (T2D, CAD, FBG, SBP, and DBP, etc.) and identified 1,173 serum (385 with known and 788 with unknown structures) and 1,016 plasma metabolites (321 known and 695 unknown) to be associated with these traits (*p* </= 0.01). In this study, we present the results of genome-wide associations identified for compounds with known structures, including 385 serum and 321 plasma lipid metabolites, using the AIDHS discovery and validation cohorts. We identified 148 lipid metabolites across 13 lipid classes that are associated with 55 genetic loci in serum, and 89 metabolites within 12 lipid classes that are linked to 40 genetic loci in plasma (see S1 and S2 Figs). and the identification of the top 36 mQTLs associations (shared between serum and plasma) and their functional significance is summarized in Table 1. In the analysis of Model 1, we identified 122 significant SNP-metabolite associations reached GWAS significance (*p* ranging from 9.7 × 10^−8^ to 1.3 × 10^−21^) in 52 metabolites after adjusting for age, sex, BMI, genetic PCs, and metabolite PCs, along with T2D and CAD. After including clinical lipids in Model 2, 236 SNP-metabolite association signals achieved GWAS significance with 52 metabolites *p* values ranging from 9.6 × 10^−8^ to 1.5 × 10^−34^ (S2 and S3 Tables). Of these 140 SNP-metabolite associations were new and not found in Model 1, while 96 associations were significant and common in both models. In contrast, 26 significant SNP-metabolite pairs from Model 1 with the specified metabolite did not retain high statistical significance after controlling for clinical lipids in Model 2. The majority of the lipids (~94%) were associated with variation at a single locus, while 6% of metabolites were associated with two loci. The Manhattan plot summarizes the detection of top genetic loci in Model 2 for serum and plasma, as shown in Figs 2 and 3. In total, we identified 236 independent SNP-metabolite association pairs, including 153 pairs in serum and 83 pairs in plasma, that achieved a genome-wide *p* value (5 × 10^−8^). Among these, 80 metabolite-SNP QTLs displayed a consistent beta direction for the minor allele, and 74 independent signals attained robust significance when combined in a meta-analysis and were selected for further analysis. To adjust for multiple comparisons, a false discovery rate (FDR) was applied in addition to the GWAS empirical *p*-value threshold of 5 × 10^−8,^ and an FDR of 1.92 × 10^−10^ (5 × 10^−8^/262), and 40 out of 84 mQTLs survived the FDR correction and were selected for MR and colocalization analysis (Fig 1 and S4 Table).

**Table 1:** Identification of top SNP-Metabolite mQTLs located in functional and non-functional gene regions.

Predictor	SNP	Ref	Alt	MAF_AIDHS	MAF_SA	MAF_EU	CADD	Genes	Gene region	Metabolites	Beta	SE	*P*</=
1:177505006	rs144877273	A	G	0.013	0.012	0.010	6.1	*ASTN1,PTP4A1P7,PAPPA2, RPS14P2,CLEC20A*	intergenic	PC O-41:11	−0.15	0.02	4.45E−11
1:197554071	rs553095017^*^	G	A	0.006	0.013	0.0004	7.3	*DENND1B*	intron	LPC O-16:0	−0.19	0.03	7.26E−13
1:198778169	rs558096054	T	C	0.007	0.019	0.003	10.6	*PTPRC*	intergenic	LPC O-16:0	−0.18	0.03	1.43E−09
1:228691329	rs547301608	A	G	0.007	0.019	0.0006	4.2	*FTH1P2, RHOU*	intergenic	PC 42:5	−0.18	0.02	1.17E−12
3:133090016	rs549235168	G	T	0.007	0.026	0.000	6.6	*TMEM108*	intron	LPC O-16:0	−0.17	0.03	9.24E−11
3:168350463	rs564390773	T	C	0.006	0.009	0.000	4.3	*EGFEM1P*	intron	PC 36:3 Isomer B	−0.12	0.02	1.55E−12
3:67985862	rs146198792	G	T	0.017	0.007	0.013	1.2	*TAFA1*	intergenic	LPC O-16:0	−0.29	0.05	8.75E−08
3:77035421	rs145555444	C	T	0.009	0.012	0.0005	6.4	*ROBO2*	intron	LPC O-16:0	−0.29	0.02	3.44E−32
4:14555392	rs78842674	C	T	0.010	0.009	0.009	1.8	*LINC00504*	intron	FA 8:0 (caprylic acid)	−0.16	0.02	3.30E−12
4:187241284	rs185569860	G	T	0.011	0.021	0.005	0.6	*LOC107986335,LINC02374, LOC339975*	intergenic	PC O-41:11	−0.17	0.02	4.59E−12
4:47220987	rs114741223	G	A	0.010	0.006	0.028	0.2	*GABRB1*	intron	PC 28:0	−0.24	0.04	1.60E−11
4:62527292	rs571141934	T	C	0.003	0.012	0.00004	2.2	*HMGN1P11,LOC100131441*	intergenic	PC 34:1	−0.29	0.04	6.71E−11
5:178100812	rs542587019	C	T	0.009	0.023	0.00004	2.3	*N4BP3,RMND5B,NHP2,GMCL2, FAM153CP*	intergenic	LPC 17:0	−0.24	0.03	3.84E−14
6:131851573	rs182396254^*^	C	T	0.010	0.009	0.009	5.8	*ENPP1*	intron	LPC O-16:0	−0.20	0.03	1.04E−13
6:79945133	rs184696002^*^	A	G	0.010	0.023	0.009	0.2	*ELOVL4*	intron	LPC O-16:0	−0.17	0.03	7.14E−11
6:8280653	rs7771158	T	C	0.029	0.039	0.019	16.9	*LOC105374910, LOC105374911*	intergenic	LPC O-16:0	−0.16	0.02	1.04E−11
7:47187689	rs542067289	A	G	0.007	0.008	0.002	0.3	*TNS3*	intergenic	PC O-41:11	−0.16	0.02	8.17E−12
8:127997847	rs75558477	C	T	0.028	0.039	0.015	0.1	*PVT1*	intron	LPC O-16:0	−0.17	0.03	4.04E−11
10:27966471	rs377346277	G	T	0.006	0.005	0.000	0.9	*ARMC4*	intron	LPC 22:4	−0.14	0.02	3.23E−11
10:3486516	rs147210772	C	T	0.010	0.020	0.008	1.9	*LINC02669,LOC105376360*	intron	PC O-41:11	−0.15	0.02	6.60E−11
10:71785048	rs397517340^*^	C	T	0.009	0.019	0.000	24.1	*CDH23*	missense	LPC O-16:0	−0.21	0.03	6.97E−14
11:134814245	rs188837178	A	C	0.007	0.007	0.012	4.0	*LINC02714,LINC02706*	intergenic	LPC O-16:0	−0.17	0.03	4.69E−11
11:23435455	rs71490680	A	G	0.013	0.008	0.028	4.4	*MIR8054,WIZP1,THAP12P4,RPS2P38*	intergenic	PC O-41:11	−0.17	0.02	1.24E−12
11:61790331	rs102275^*^	T	C	0.205	0.165	0.362	5.5	*TMEM258*	intron	PC 38:4 Isomer C	−0.22	0.02	1.01E−35
11:61796827	rs4246215^*^	G	T	0.188	0.140	0.369	12.6	*FEN1*	UTR3	PC 38:4 Isomer C	−0.23	0.02	1.90E−39
11:61803311	rs174547	T	C	0.177	0.137	0.347	5.5	*FADS1*	intron	PC 38:4 Isomer C	−0.24	0.02	2.00E−40
										TG 53:4	0.17	0.03	1.73E−10
										FA 20:4	−0.09	0.02	1.06E−08
11:61817672	rs174562	A	G	0.178	0.137	0.345	4.1	*FADS2*	intron	CE 20:4	−0.20	0.02	6.47E−26
11:61830500	rs1535	A	G	0.180	0.136	0.350	2.4			PC 38:4 Isomer C	−0.15	0.01	1.89E−27
15:62431890	rs568020910	A	G	0.010	0.025	0.000	1.3	*TLN2*	intergenic	LPC 16:1	−0.30	0.05	7.45E−11
15:78123074	rs147988517	G	A	0.012	0.016	0.003	0.01	*CIB2*	intron	LPC O-16:0	−0.19	0.03	5.01E−13
16:66424543	rs112061126^*^	T	C	0.013	0.024	0.001	10.7	*CDH5,BEAN1,TK2*	intergenic	LPC 17:0	−0.21	0.03	2.08E−11
20:16661826	rs191368038	C	A	0.016	0.004	0.012	8.5	*RPLP0P1,KIF16B,RPL7AP13,SNRPB2,OTOR*	intergenic	LPC O-16:0	−0.21	0.03	2.74E−14

* There were other SNPs (rs542112266, rs578160766, rs142178143, rs568929890, rs174528, rs174541, rs113404079) that cleared FDR but were in linkage disequilibrium (LD) and are not represented in the table; AIDHS, Asian Indian Diabetic Heart Study; SA, South Asians; EU, Europeans. The *p*-value was calculated using multivariate regression.

### Replication studies and the discovery of population-specific mQTLs

For validation studies, we examined the association of all 236 mQTLs (*p* </= 5 × 10^−8^) identified in AIDHS/SDS in other independent cohorts available with lipidomics profiles. We considered our mQTL variant(s) to be new based on their associations with the metabolite or related metabolites, and CV disease and risk factors were not listed in the public repositories in context to the metabolite or the trait. For instance, the new mQTLs for LPC O-16:0 (*ELOVL4, ELP4, PTPRC/CD45, TMEM108, RORA, CDH23, HELZ2, and ROBO2*), were only detected in our study and no association was observed in any other published study. We further extended our search ± 1.5 Mb from our mQTLs in other studies and did not find any variant in LD with our lead SNP or proxy SNP showing association for the specific mQTL or other metabolites. However, extending this search beyond 6Mb from our lead variant in the *TNS3* (rs371318240) associated with LPC O-16:0, we identified two variants located upstream to the TNS3 variant encoded by *SUGCT* gene (rs554961731 and rs570248035) showing association with LPC O-16:0 in Cadby and colleagues [42]. However, these variants were not in LD with our lead SNP in the TNS3 region. Further evaluation of the *SUGCT* gene in AIDHS/SDS did not yield any significant association with LPC O16:0 or any other metabolite (S19 Table).

The mQTLs identified on the chromosome 11 region for FAs, PCs, and TGs were independently replicated in other external cohorts, SA and EU studies. In contrast to studies conducted in the EU, which have predominantly identified the chromosome 11 region (*FADS1/2/3*) as harboring loci linked to FAs, our research found no significant associations with any FA reaching the GWAS empirical p-value threshold for this region, except for arachidonic acid (FA 20:4) in Asian Indians that disappeared after adjusting for clinical lipids (S2 Table). In contrast, we identified a mQTL signal associated with the *FADS1*, *FADS2*, *TMEM258*, *FEN1*, and *MYRF* genes in this region with PC 38:4 (C) (S5 Table). The same variants rs174547 (*FADS1*) (Beta ± SE = −0.15 ± 0.01; *p* </= 7.2 × 10^−115^) and rs102275 (*TMEM258*) (Beta ± SE = −0.14 ± 0.01; *p* </= 1.5 × 10^−105^) were significantly associated with PC 38:4 (C) in Pakistani cohort, as seen in the AIDHS/SDS and were in the same direction. However, the same variants rs174547 (*FADS1*) (Beta ± SE = −0.47 ± 0.02; *p* </= 5.8 × 10^−98^) and rs102275 (*TMEM258*) (Beta ± SE = −0.47 ± 0.02; *p* </= 3.9 × 10^−99^) were associated with another related PC (PC 38:5) in the BHS cohort in Australians and PC18.2.0_20.4.0/PC38:6 in Finnish (Beta ± SE −0.17 ± 0.02, *p* </= 9.6 × 10^−24^) (S5 Table). Additionally, we also detected many robustly associated variants from different genomic regions with FA18:0;(2OH), represented by *ADGRB2*, *PPARGC1α*, *SPP1*, *HDAC5*, *PTK7*, and *TRIP4* genes, which have not been reported in any genome-wide study for lipid metabolites (S3 Table).

### Phenotypic correlation of serum metabolites with other cardiometabolic traits

We further determined the phenotypic correlation between the top mQTLs with other cardiometabolic traits, and significant results are presented in heat maps (S3 Fig). Circulating levels of all FAs correlated positively and significantly with FBG in AIDHS/SDS. Similarly, glycerophospholipids such as LPCs showed a significant positive correlation (ranging from *r* = 0.14 to 0.24 and *p* </= 2.7 × 10^−13^ to 1.1 × 10^−38^) with FBG except for LPC 16:1. On the other hand, most of the PCs correlated negatively with FBG (ranging from *r* = −0.04 to −0.14 and *p* </= 0.04 to 1.7 × 10^−14^). Likewise, most high-carbon-containing sphingolipids (SMs), SM d36:1 and SM d41:2 Isomer B, were reduced with FBG and TG, while high-carbon-containing neutral lipids, TG 50:2 and TG 50:6, were significantly reduced with increased FBG (S3 Fig).

### Association of mQTL variants with cardiometabolic phenotypes in UKBB

We further explored the association of all 236 mQTL variants with blood glucose, glycated hemoglobin (HbA1c), creatinine, C-reactive protein (CRP), apolipoprotein B (ApoB), atherosclerosis, arterial embolism and thrombosis, cerebral infarction, intracranial hemorrhage, and subarachnoid hemorrhage using phenotype data from UKBB. We identified 205 significant associations of top mQTL variants with cardiometabolic traits. The vast majority of the variants were from the *FADS* region represented by *FADS1/2*, *MYRF*, *FEN1*, and *TMEM258* genes, which showed strong association with ApoB, CRP, creatinine, glucose, and HbA1C levels with p values ranging between 5.0 × 10^−8^ to 2.5 × 10^−89^. These genes represented the top mQTLs for PC 38:4 (C), TG 53:4, CE 20:4, FA 20:4, FA 20:5. Another mQTL for SM 35:2;2O|SM 18:2;2O/17:0 in *ELP1* (chromosome 9) showed a strong association with glucose levels (*p* </= 3.1 × 10^−08^) and HbA1C (*p* </= 2.8 × 10^−07^). Additionally, the mQTL signals identified for FA 18:0;(2OH) in *PTK7*, and *PLCG2* for LPC O-16:0 were associated with ApoB, creatinine, and CRP levels at a modest *p* </= 1.3 × 10^−3^ (S6 Table).

### Univariable Mendelian randomization (MR) and sensitivity analysis

**Fig 4:**
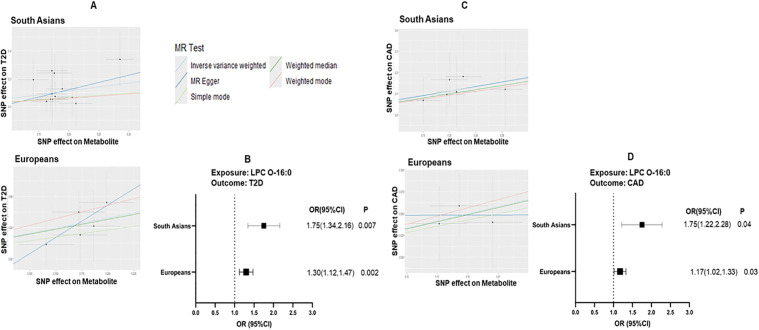
Scatter plots and forest plots showing genetic association between LPC O-16:0, T2D, and CAD. Using univariable MR and sensitivity analysis applying the IVW, weighted median, weighted mode, maximum likelihood, and MR-Egger methods for fixed effects (FE) and random effects (RE) **(A)** Scatter plots on top left to bottom show the effect of LPC O-16:0-associated SNPs on the exposure (metabolite) on the x-axis and outcome (T2D) on the y-axis with each dot represent individual SNP in SA, and EU. **(B)** Forest plot shows the IVW ORs and p-values of LPC O-16:0-associated SNPs on T2D in SA and EU. **(C)** Scatter plots on top left to bottom right show the effect of LPC O-16:0-associated SNPs on the exposure (metabolite) on the x-axis and outcome (CAD) on the y-axis, in SA, and EU. (D) Forest plot shows the IVW ORs and *p*-values of LPC O-16:0-associated SNPs on CAD in SA, and EU. MR, Mendelian randomization; T2D, type 2 diabetes; CAD, coronary artery disease; SA, South Asian; EU, European. The *p*-value was calculated using IVW regression.

To further evaluate the causal link between mQTLs and T2D or CAD, we utilized genetic instruments in the application of two-sample MR. Using univariable MR and sensitivity analysis applying the IVW, weighted median, weighted mode, maximum likelihood, and MR-Egger methods for fixed effects (FE) and random effects (RE), we tested the associations of all significantly associated mQTL on T2D or CAD and other cardiometabolic risk factors in other ancestries. We followed a strict quality control and systematic approach to ensure that a genetic instrument derived from one ancestry is valid for other ancestry using univariable 2-sample MR. We ensured that the genetic instrument was strongly associated with the exposure in the target (ancestry similar or non-similar) population based on regression (beta) coefficients and p-value/F statistics accounting for the LD and allele frequency. LPC O-16:0 showed significant positive associations with T2D and CAD. The MAFs for LPC O-16:0 associated mQTLs used for MR analysis in AIDHS, SAs, and EUs are mentioned in S7a Table. Genetically instrumented per 1SD increment of blood LPC O-16:0 level would increase T2D risk to over ~2-fold in SAs from UKBB OR 1.75 (95% CI [1.34,2.16]; *p* </= 0.007). A similar but less significant trend was seen in EUs, because the 7 top variant mQTLs of LPC O-16:0 detected in Asian Indians in this study were monomorphic in EUs. Also, the increased concentration of LPC O-16:0 would increase the risk of CAD in UKBB SA OR 1.75 (95% CI [1.22,2.28]; *p* </= 0.04) and UKBB EU OR 1.17 (95% CI [1.02,1.33]; *p* </= 0.03) (Fig 4; S8 and S9 Tables). Both heterogeneity and pleiotropy were minimal and not significant for both the LPC O-16:0 effect on T2D and CAD (S10a and S10b Table).

Genetically instrumented decrease in PC 38:4 (C) due to variation in *FADS1/2* and *FEN1*, variants showing negative ORs (0.90 to 0.77; *p* </= 8.1 × 10^−12^ to 1.2 × 10^−35^) was associated with a significantly increased risk for CAD in AIDHS/SDS with ORs and *p* values ranging from (1.28 to 1.36; *p* </= 0.007 to 9.6 × 10^−4^) (S5 Fig). The effects of these variants on CAD in SA from UKBB were in the same direction but were not significant (S11 Table). The MAFs for PC 38:4 (C) associated mQTLs used for MR analysis in AIDHS, SAs, and EUs are mentioned in S7b Table. On the other hand, in the UKBB EUs, the beta directional effects were opposite. Aside from the two metabolites (LPC O-16:0 and PC 38:4 (C)), no other lipid metabolites could be confirmed to have a causal association with T2D, CAD, or other cardiometabolic risk traits such as glucose, TG, SBP, and DBP using the MR approach. The genetic variance accounting for phenotypic variance in LPC O-16:0, measured by *R*-squared (*R*^2^), varied from 0.01% to 4.5% for each independent lead variant, while the aggregate *R*^2^ of the independent variants ranged from 15.1% to 20.9%. However, the total genetic variance (*R*^2^) explained by these variants for increasing the risk of T2D and CAD was 1.8% and 1.6%, respectively.

### Association between lipid metabolites with PRS

We next tested the association of the ancestry-specific and EU-derived PRS for T2D and CAD with all 262 mQTLs using a multivariate linear regression analysis adjusted for age, gender, and BMI. None of the top lipid metabolites revealed significant and positive associations with T2D PRS in both ancestry-specific or EU-derived polygenic scores (S12a Table). On the other hand, several polyunsaturated fatty acids (PUFAs) were positively associated with CAD PRS. Specifically, FA 18:1 (Beta ± SE = 0.007 ± 0.001; *p* </= 1.26 × 10^−14^), FA 18:2 (Beta ± SE = 0.006 ± 0.0009; *p* </= 3.77 × 10^−13^), FA 20:3 (Beta ± SE = 0.006 ± 0.0007; *p* </= 3.18 × 10^−17^), and FA 20:4 (Beta ± SE = 0.005 ± 0.0008; *p* </= 1.99 × 10^−11^) were significantly increased with increased PRS (S12b Table), showing the *R*^2^ range between 4.0% to 4.9% and 2.4% to 3.1% in SA and EU PRS, respectively. The genome-wide metabolite PRS (for the individual metabolite) of LPC O-16:0 (using 2,577 significant SNPs with *p* </= 10^−4^) revealed a strong association of PRS with LPC O-16:0, showing Beta ± SE 0.51 + 0.003; *p* </= 2.72 × 10^−80^, after adjusting for age, gender, BMI, T2D, CAD, and clinical lipids (S13a Table) explaining the genetic variance of 23.1% (*R*^2^). Similarly, the genome-wide metabolite-PRS for PC 38:4 (C) (using 2,538 significant SNPs with *p* </= 10^−4^) showed a robust association with PC 38:4 (C) metabolite (Beta + SE 0.70 + 0.002; *p* </= 1.84 × 10^−265^) after adjusting for covariates (S13a Table), and with the *R*^2^ of 36.7%. However, despite the high genetic variance, neither of the metabolite PRS for LPC O-16:0 nor PC 38:4 (C) could significantly predict the risk of T2D or CAD. LPC O-16.0 PRS showed an OR 0.98 (95% CI [0.58,1.39]; *p* </= 0.94) for T2D and OR 1.03 (95% CI [0.48,1.58]; *p* </= 0.91) for CAD, and showed an *R*^2^ range of 0.2% to 0.9%, respectively. Similarly, PC 38:4 (C) PRS showed an OR 0.97 (95% CI [0.52,1.42]; *p* </= 0.89) for T2D and OR 1.00 (95% CI [0.39,1.61]; *p* </= 0.99) for CAD, having an *R*^2^ range of 0.4% to 0.2%, respectively (S13b Table). Using the polygenic score only from the top 44 SNPs of LPC O-16:0, the PRS analysis predicted that increased LPC O-16:0 would significantly increase the T2D risk in AIDHS/SDS showing an OR 1.06 (95% CI [1.02,1.09]; *p* </= 0.004) in Models 1 and OR 1.05 (95% CI [1.02,1.09]; *p* < / = 0.005) in Model 2 with clinical lipids. However, the *R*^*2*^ value for T2D was marginally increased from 0.2% of the original metabolite to 0.7% (using the high-impact 44 SNPs) (S13b Table).

### Colocalization analysis of mQTLs with disease outcomes, co-sharing, and pathways

**Fig 5:**
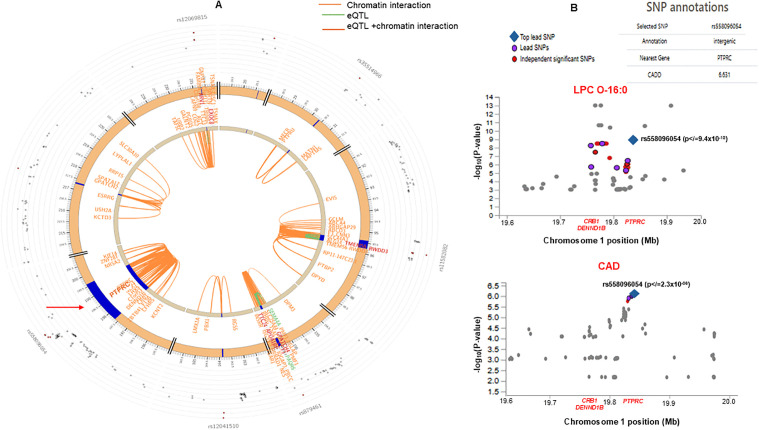
Colocalization and functional mapping to characterize genomic loci using FUMA. **(A**) Circos plot depicting the colocalization of the eQTL CRB1, DENND1B, and PTPRC region (chromosome 1q31.1-q32.1) showing cis-eQTL interaction and chromatin interaction with other genes on chromosome 1. The outermost layer of the Circos plot is the Manhattan plot showing SNPs with *p* values <0.05, and the SNPs with colors show LD (r2) with the top independent SNP: red r2 > 0.8, orange r2 > 0.6, blue r2 > 0.3, and other SNPs are colored gray. **(B)** Regional association plot of Chromosome 1 (197,201,504−198,757,476) region showing association with LPC O-16:0 and CAD identified by the top variant (rs558096054) in the *PTPRC* by FUMA. The p-value was calculated using multivariate regression.

Further fine mapping analysis by FUMA of chromosome 1q31.1-q31.3 associated with LPC O-16:0 (containing *PTPRC*, *CRB1*, and *DENND1B* genes) demonstrated strong chromatin interactions with several other genes, including *CFHR5*, *KIF14*, *ZNF281*, *ZBTB41*, *NEK7*, and *KCNT2*, reported previously to be associated with CAD and CV traits in the GWAS catalogue (Fig 5A and S14 Table). Colocalization analysis also identified the region within *PTPRC* (rs558096054), linked to LPC O-16:0 (in regression analysis) to be associated with an increased risk of CAD OR 3.35 (95% CI [2.62,4.07]; *p* </= 2.3 × 10^−6^) (Fig 5B). Moreover, the top lead SNP in the *PTPRC* and other variants associated with LPC O-16:0 (rs142028924; *RORA*) and rs145555444 (*ROBO2*), and (rs540305535; *LEPR*) have been previously reported in GWAS of CRP, Asthma, blood lipid phenotypes, Inflammatory bowel disease (IBD), SBP, T2D, BMI, waist-to-hip ratio (WHR), apolipoprotein A1 (ApoA1) with p-values ranging from 3.0 × 10^−08^ to 1.0 × 10^−56^ (Table 2).

**Table 2:** Genomic regions identified by the top lead variant showing co-sharing with lipid species and other diseases.

Predictor	SNP	Genes	Colocalised lipids	Associated Diseases	*P*-value range (</=)
1:118144267:T:C	rs151050045	*SPAG17*	FA, PC, TG	OA	6.00E−15
1:198778169:C:T	rs558096054	*PTPRC*	LPC, PC	Asthma, IBD, SLE, T1D, RA	8.00E−09 to 3.00E−15
1:212390679:C:T	rs139562877	*PACC1*	PC, PE	HDL-C	5.00E−10
1:5951468:A:G	rs548140784	*NPHP4*	PC	AD	7.00E−11
1:65612171:G:A	rs540305535	*LEPR*	DG, LPC	CRP, LDL-C, HDL-C, BMI, CAD, ApoA1, TC, T2D, Obesity	2.00E−09 to 9.00E−276
1:85370753:A:G	rs1498377	*DDAH1*	FA, TG	MS, BMI	3.00E−08 to 4.00E−09
2:205404049:G:A	rs533667777	*PARD3B*	DG, GlcCer, PC, TG	BMI, Weight, VAT, WBFM, AML, T2D, BF%, AD, MetS, HC	1.00E−08 to 3.00E−24
3:16206080:A:G	rs1217247018	*GALNT15*	TG	AML	6.00E−15
3:168290188:A:G	rs369841933	*EGFEM1P*	PC, PE	T2D, MetS, BMI, DR	9.00E−09 to 7.00E−12
3:186475647:A:G	rs6767055	*LINC02052*	Cer, TG	SYSBP, DYSBP	3.00E−08 to 8.00E−10
3:193670114:G:A	rs1006371374	*OPA1*	FA, TG	DYSBP	2.00E−08
3:67985862:T:G	rs146198792	*TAFA1*	LPC, TG	HOMA-B	5.00E−08
3:77035421:T:C	rs145555444	*ROBO2*	LPC	BMI, T2D	4.00E−15 to 7.00E−16
3:85264128:G:A	rs115464251	*CADM2*	Cer, SM	BMI, vitamin D levels, MetS, SYSBP, Glaucoma, Obesity	3.00E−11 to 1.00E−34
4:139627559:T:G	rs706343	*QKILA*	Cholesterol	AD	4.00E−08
4:38102009:T:C	rs547108940	*TBC1D1*	Cer, GM3, PE	CRP	4.00E−08
6:68299495:G:T	rs367833390	*LINC02549*	PC, SM	AD	1.00E−09
6:79945133:G:A	rs184696002	*ELOVL4*	LPC	WHR	3.00E−10
7:107555316:C:T	rs188763002	*COG5*	Cer, PI, SM	ApoA1, LDL-C, HDL-C, Knee OA, AF, vitamin D levels, CAD, HbA1c, TC	2.00E−08 to 6.00E−17
7:132401050:C:T	rs1560769	*PLXNA4*	FA	BMI, CRC	3.00E−09 to 9.00E−10
7:95433958:G:A	rs740265	*PON2*	DG	HDL-C, ApoA1	5.00E−10 to 1.00E−11
8:14665297:G:A	rs6984678	*SGCZ*	FA, PC, SM	BMI, AML, T2D, MetS, HDL-C	4.00E−08 to 2.00E−18
9:108900544:T:C	rs149529575	*ELP1*	SM	FBG, IGF-1	4.00E−09 to 3.00E−16
9:28679024:C:T	rs17451410	*LINGO2*	FA	BMI, TC, HDL-C, T2D, FBG, ApoA1 levels, BF%, MetS, WHR, Obesity, CRP, IA, HTN	2.00E−08 to 3.00E−46
9:9577501:A:C	rs142980516	*PTPRD*	Cer, FA, GM3, PC, SM	AML, SYSBP, AF, DYSBP, BMI, T2D	6.00E−11 to 1.00E−18
11:33893827:A:G	rs117174779	*LMO2*	FA	SYSBP, DYSBP	7.00E−08 to 5.00E−09
11:5390842:G:A	rs11037191	*OR51B5, OR51M1*	PC, SM	AD	1.00E−09
11:61776027:C:T	rs174528	*MYRF*	FA, LPC, PC, PE, TG	TG, Chron’s disease, CRC, TC, CID, Asthma, RA, IBD,AS	4.00E−08 to 9.00E−21
11:61790331:C:T	rs102275	*TMEM258*	LPC, PC, PE, TG	TG, Chron’s disease, CRC, LDL-C, TC, Asthma, ApoB, Glycemic traits, HDL-C	2.00E−08 to 1.00E−84
11:61796827:T:G	rs4246215	*FEN1*	FA, LPC, PC, PE, PI, TG	IBD	2.00E−15
11:61798436:C:T	rs174541	*FADS2*	FA, LPC, PC, PE, PI, TG	TG, Chron’s disease, CRC, LDL-C, TC, Asthma, IBD, AS, T2D, Weight, FBG	3.00E−08 to 5.00E−45
11:61803311:C:T	rs174547	*FADS1*	FA, LPC, PC, PE, PI, TG	TG, Chron’s disease, LDL-C, TC, Asthma, RA, IBD, ApoB, HDL-C, PCSK9, ApoA1	2.00E−08 to 6.00E−95
11:95111251:T:C	rs654543	*ENDOD1*	PC	AML	3.00E−19
12:128315124:G:A	rs10744375	*TMEM132C*	DG	FBG, AML, AD	2.00E−08 to 1.00E−12
12:26764911:C:T	rs11608906	*ITPR2*	LPC	SYSBP, TG, LDL-C, TC, HDL-C, T2D, RCC, TL, BF%, AF, MetS, WHR, CVD, DYSBP, EC,BCC	1.00E−08 to 5.00E−18
13:39446539:T:G	rs373374766	*LHFPL6*	TG	AD	1.00E−10
13:62729678:A:G	rs543869557	*LINC00448*	PE	AML	2.00E−10
14:63830277:C:T	rs544096674	*SYNE2*	CAR, Cer, FA, GlcCer, GM3, LPC, LPE, PC, PE	TG, Chron’s disease, LDL-C, TC, AF, IGF1, PC, HbA1c, Psoriasis, CES, CD	3.00E−09 to 4.00E−63
14:64747934:A:G	rs754435	*PLEKHG3,SPTB*	DG	TG, HDL-C	3.00E−10 to 7.00E−10
14:79217330:T:C	rs79703367	*NRXN3*	CAR, Cer, PC, PE, SM, TG	BMI, T2D, Weight, MetS, WHR, Obesity, AD, HbA1c, LEP, DR, VAT	3.00E−08 to 3.00E−46
15:53318816:C:A	rs561217563	*LINC02490*	HexCer, LPC	BMI, LDL-C, TC, T2D, MetS, CRP	8.00E−09 to 8.00E−15
15:60867006:T:C	rs142028924	*RORA*	LPC	CRP, Asthma, BMI, HDL-C, TG, CVD, IBD, SYSBP, T2D, WHR, ApoA1	3.00E−08 to 1.00E−56
16:65124203:A:G	rs9922276	*CDH11*	CAR, FA, PC	COPD, OA, Glaucoma, AD	2.00E−08 to 2.00E−12
16:81895774:A:G	rs13333348	*PLCG2*	FA, LPC	IBD, CAD, AD, APHD, IGF1	5.00E−10 to 7.00E−18
17:6587006:A:G	rs535675981	*KIAA0753*	Cer, PC, PE	SYSBP	2.00E−14
17:69249803:T:C	rs1420905	*ABCA5*	Cer, PC	TC, ApoB, LDL-C	3.00E−10 to 4.00E−22
18:27153281:A:C	rs75037302	*CHST9*	FA	BC, SYSBP, DYSBP	2.00E−08 to 8.00E−16
18:57757684:C:A	rs370257755	*ATP8B1*	Cer, FA, GM3, PE, PI	TC, LDL-C, Cancer	1.00E−08 to 8.00E−15
19:7966827:T:C	rs540585493	*ELAVL1*	DG	LDL-C	3.00E−15
21:34667765:T:C	rs117184570	*CLIC6*	CE, Cer, LPC, PC, PE, SM	PC	3.00E−08
22:24852307:A:G	rs1291074843	*SGSM1*	LPC	T2D, FBG, INS-related traits, HbA1c	2.00E−08 to 5.00E−09

AD, Alzheimer’s disease; AF, atrial fibrillation; AML, acute myeloid leukemia; APHD, acute pulmonary heart disease; ApoA1, apolipoprotein A1 levels; ApoB, apolipoprotein B levels; AS, aortic stenosis; AS, aortic stenosis; BC, breast cancer; BCC, basal cell carcinoma; BF%, body fat percentage; BMI, body mass index; CAD, coronary artery disease; CD, cardiac dysrhythmias; CE, cholesteryl esters; Cer, ceramides; CID, chronic inflammatory diseases; COPD, chronic obstructive pulmonary disease; CRC, colorectal cancer; CRP, C-reactive protein levels; CS, cardioembolic stroke; CVD, cardiovascular disease; DG, diacylglycerols; DR, diabetic retinopathy; DYSBP, diastolic blood pressure; EC, endometrial cancer; FA, fatty acids; FBG, fasting glucose; GlcCer, glucosylceramide; GM3, monosialoganglioside; HbA1c, glycated hemoglobin levels; HC, hypertrophic cardiomyopathy; HDL-C, HDL cholesterol; HexCer, hexosylceramide; HTN, hypertension; IA, intracranial aneurysm; IBD, inflammatory bowel disease; IGF1, insulin-like growth factor 1 levels; INS-related traits, insulin-related traits; LDL-C, LDL cholesterol levels; LEP, leptin levels; LPC, lysophosphatidylcholine; MetS, metabolic syndrome; MS, multiple sclerosis; OA, osteoarthritis; PC, prostate cancer; PC, phosphatidylcholine; PE, phosphoethanolamine; PI, phosphatidylinositol; RA, rheumatoid arthritis; RCC, renal cell carcinoma; SM, sphingomyelin; SYSBP, systolic blood pressure; T2D, type 2 diabetes; TC, total cholesterol levels; TG, triglyceride levels; TL, telomere length; VAT, visceral adipose tissue; vitamin D levels, serum 25-hydroxyvitamin D levels; WBFM, whole body fat mass; WHR, waist-hip ratio; Source of disease association-GWAS Catalogue and publicly available data and literature. The *p*-value was calculated using multivariate regression.

**Fig 6:**
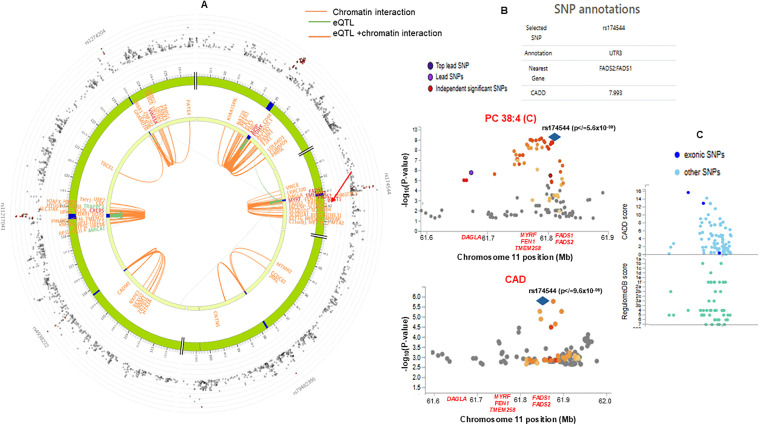
Colocalization and functional mapping to characterize genomic loci using FUMA. **(A)** Circos plot depicting the colocalization of the eQTL FADS region (chromosome 11q12-q13.1) showing cis-eQTL interaction and chromatin interaction with other genes on chromosome 11. The outermost layer of the Circos plot is the Manhattan plot showing SNPs with *p* values <0.05, and the SNPs with colors show LD (r2) with the top independent SNP: red r2 > 0.8, orange r2 > 0.6, blue r2 > 0.3, and other SNPs are colored gray. **(B)** Regional association plot of the *FADS* region Chromosome 11 (61,752,636−61,788,518) shows evidence of association with PC 38:4 (C) and CAD identified by rs174544 by FUMA. **(C)** CADD and RegulomeDB score plots of the SNPs located under the *FADS* region. The *p*-value was calculated using multivariate regression.

Interactive analysis of eQTL association and chromatin interaction mapping in FUMA, we detected strong evidence of *cis-*eQTL and chromatin interactions within the 11q12.2 region encompassing *FADS1/2*, *MYRF*, *FADS3*, *DAGLA*, with other loci like *LRRC10B*, *APIP*, *PDHX*, *CXCR5*, *SLC37A4*, and *GRAMD1B*, etc., previously reported to be associated with CAD and CV traits in the GWAS catalogue (Fig 6A and S15 Table). The FADS1 polymorphism (rs174544), strongly associated with PC 38:4 (C), TG 53:4, and FA 20:4 in this study, was also linked to CAD risk in the AIDHS, as shown by colocalization analysis in FUMA (Fig 6B). Interestingly, the individuals with high CAD PRS exhibited significantly lower levels of PC 38:4 (C). For example, the mean levels of PC 38:4 (C) in patients within the highest quartile of PRS were significantly lower (0.91 ± 0.053 µM) compared to those in the lowest quartile of PRS (1.09 ± 0.039 µM) (*F* = 14.22, two-tailed *p* </= 0.005). Regional plots show well-defined peaks for the metabolite and CAD association represented by a 3’UTR variant (rs174544) within the *FADS1* region OR 1.36 (95% CI [1.18,1.51]; *p* </= 9.6 × 10^−6^) with a CADD score of 7.993 (Fig 6B). Many variants within this region also showed high CADD and RegulomeDB scores. This same *FADS1* rs174544 variant has been linked with comorbidities such as Crohn’s disease, asthma, rheumatoid arthritis, and IBD, and has been shown to regulate clinical parameters such as TG, TC, LDL-C, Apo B, and Apo A1 levels (Table 2). The heritability of the top metabolites achieving GWAS significance is detailed in S16 Table. Notably, FA 18:0;(2OH) showed the highest heritability of 0.78. Both LPC O-16:0 and PC 38:4 (C) exhibited heritability of 0.16 and 0.13, respectively.

The interactive visualization using GSEA validated established candidate genes and identified other prospective genes through the use of eQTL mapping and chromatin interaction mapping. Using GSEA, we identified several crucial pathways that were significantly over-represented in the mQTLs for the LPCs and specifically the ether-containing LPC O-16:0. Overrepresentation of SLIT-ROBO signaling, AMP signaling, leptin and adiponectin signaling, RORA and PGC1α signaling were the most relevant pathways due to genetic variation in *ROBO2*, *DENN1B*, *LEPR*, *ENPP1*, and *CRB1* genes, connected with circulating LPC O-16:0 (Table 3). ROBO2 proteins bind with SLIT proteins in the cell signaling protein complex, which is involved in many diverse physiological and pathological functions, including axon guidance and angiogenesis [62]. The *LEPR* gene transcribes the leptin receptor protein, which regulates the leptin hormone responsible for controlling appetite and body fat [63,64]. Transcription factor *RORA* recruits *PGC1α* and histone acetylase p300 by binding to DNA response elements, activating transcription of the downstream genes [65]. Dysregulation of these pathways collectively contributes to metabolic diseases, immune system dysfunction, cancer, and inflammation, neural development, obesity, diabetes, and vascular dysfunction (Table 3).

**Table 3:** Over-represented pathways regulated by genes associated with metabolites.

Metabolites	Pathways	Source	Overlapping genes	All pathways genes (N)	*P*</=
LPC O-16:0, DG 34:1	AMPK signaling pathway – Homo sapiens (human)	KEGG	*TBC1D1;LEPR*	120 (120)	4.13E−03
	reversal of insulin resistance by leptin	BioCarta	*LEPR*	9 (9)	7.35E−03
	Leptin and adiponectin	Wikipathways		10 (10)	8.16E−03
	Signaling by Leptin	Reactome		11 (11)	8.97E−03
	Leptin Insulin Overlap	Wikipathways		17 (17)	1.38E−02
LPC O-16:0	Regulation of lipid metabolism by PPAR alpha	Reactome	*HELZ2*	20 (20)	1.63E−02
	Development and heterogeneity of the ILC family	Wikipathways	*RORA*	32 (32)	2.59E−02
	Translocation of SLC2A4 (GLUT4) to the plasma membrane	Reactome	*TBC1D1*	34 (34)	2.75E−02
	Transcriptional regulation of white adipocyte differentiation	Reactome	*HELZ2*	47 (47)	3.78E−02
	Inflammatory bowel disease – Homo sapiens (human)	KEGG	*RORA*	65 (65)	5.20E−02
	HIF-1-alpha transcription factor network	PID		66 (66)	5.28E−02
LPC O-16:0, DG 34:1	Adipocytokine signaling pathway – Homo sapiens (human)	KEGG	*LEPR*	69 (69)	5.51E−02
PC 38:4 Isomer C, FA 20:5, TG 53:4	Alpha Linolenic Acid and Linoleic Acid Metabolism	SMPDB	*FADS1;FADS2*	6 (6)	9.97E−07
	oleate biosynthesis	HumanCyc		8 (8)	1.86E−06
	Omega-9 FA synthesis	Wikipathways		12 (12)	4.38E−06
	alpha-linolenic acid (ALA) metabolism	Reactome		13 (13)	5.18E−06
	alpha-linolenic (omega3) and linoleic (omega6) acid metabolism	Reactome		13 (13)	5.18E−06
	eicosapentaenoate biosynthesis	HumanCyc		13 (13)	5.18E−06
	Omega-3-Omega-6 FA synthesis	Wikipathways		14 (14)	6.04E−06
	Omega-3 fatty acid metabolism	EHMN		16 (16)	7.97E−06
	Biosynthesis of unsaturated fatty acids − Homo sapiens (human)	KEGG		27 (27)	2.33E−05
	Omega-6 fatty acid metabolism	EHMN		28 (28)	2.51E−05
	Cholesterol metabolism (includes both Bloch and Kandutsch–Russell pathways)	Wikipathways		46 (46)	6.85E−05
	Linoleate metabolism	EHMN		72 (72)	1.69E−04
	Fatty acid metabolism	Reactome		176 (177)	1.01E−03
	PPAR signaling pathway – Homo sapiens (human)	KEGG	*FADS2*	74 (76)	2.19E−02

### Fine mapping analysis to calculate PIP and Z-scores

The fine mapping analysis of the mQTL regions associated with LPC O-16:0 yielded stronger PIP scores for the top mQTL (rs558096054; *PTPRC*), showing a PIP = 0.99; Z-score = −6.38 (S17a Table). This same SNP was associated with increased risk of CAD OR 3.35 (95% CI [2.62,4.07]; *p* </= 2.3 × 10^−6^) through colocalization analysis. Similarly, fine mapping of the *FADS* region showed a strong association with PC 38:4 (C) (regression) for a variant (rs174564; *FADS2*) with a PIP of 0.61; Z-score = −9.03. This SNP was in strong LD (*R*^2^ = 0.98) with 3′UTR variant (rs174544) within the *FADS1* region which showed increased CAD risk through colocalization analysis (S17b Table).

We also performed regression analysis excluding metabolite PCs from the model (Model 3) and compared results across adjusted and unadjusted models. Overall, in the combined serum and plasma, 29.5% of GWAS significant SNPs became less significant while 71.5% associations remained significant at the GWAS p value or higher. At the same time, the average change in the *β* values was <2 units between models 2 and 3. Average *β* varied significantly between model 1 and the other two models (2 and 3) because of the addition of clinical lipids in models 2 and 3 (S18 Table). We have presented the change of beta between models 2 and 3 in S6 Fig. Despite these differences, the (*β*) direction of associations remained consistent in all associated SNPs across all models. It was interesting to note that the major QTLs identified for LPC O-16:0, PC34:1, FA18:0(2OH), DG O-37:1|DG O-21:0_16:1, and PC38:4 remained statistically significant in all three models. However, the QTL association for TG 53:4 and CE20:4 in the FADS1/2 region did not achieve GWAS *p*-values in Model 3.

## Discussion

We previously reported the untargeted lipidomics profiles of 3,000 Asian Indian participants, identifying lipid metabolites associated with T2D, particularly the role of essential FAs and their derivatives in metabolic and cardiovascular disease [13]. In this study, we investigated the genetic architecture of lipid metabolite species and how their interactive influences alter the metabolism and disease outcomes by performing mGWAS and replication studies using multiple cohorts of individuals from SA and EU ancestries. Of the 236 associations (mQTLs) that achieved genome-wide significance, 151 SNPs were from the known loci, while the remainder were from intergenic regions (Table 1). About 33% of these genetic signals were co-shared among disease phenotypes in several previous GWAS of chronic diseases, including lipids, T2D, CV phenotypes, inflammation, Alzheimer’s disease, cancers, or asthma, suggesting common genetic effects shared across disease phenotypes as summarized in Table 2.

Many associations between SNPs and lipid metabolites remained strong even after adjusting for conventional lipid levels (Model 2). This adjustment helped eliminate potential false-positive associations that other clinical lipids could influence and confirmed that the observed genetic associations were independent of clinical lipids. Because of ethnic and genetic differences, most SNP-lipid associations identified in the Punjabi sample were ancestry-specific and not identified in independent studies of EUs. Either the same lead variant was not found in EU studies, or the index variant was not associated with the same lipid species. Of all 236 mQTLs identified in this study, we examined the association of the same SNPs or their proxies or the genes encoding these variants across all previously reported studies.

Our study detected ~10 variants from the *FADS* cluster (chr11q12-q13.1 region) that were associated with the same lipid class or species as reported in earlier published studies in EUs from Australia and Finland [42–44]. Interestingly, our study detected new ancestry-specific mQTLs in AIDHS/SDS, whose association was validated only in SAs from the UKBB or Pakistan (S5 Table). For instance, we identified three ancestry-specific mQTL that achieved genome-wide significance in Sikhs, which were also replicated in the Pakistani cohort (S4 Fig). Specifically, we found two mQTL associated with PC 38:4 (C) involving *FADS1* (rs174547) and *TMEM258* (rs102275). PC38:4, PC38:5, and PC38:6 -all carry 38 carbon atoms but differ by the position of the double bond at the 4, 5, or 6 positions, respectively, in their fatty acid chain, which may change their biological and physical properties in addition to the specific structural configuration affected by isomer C or A. Both *FADS1* and *FADS2* encode FA desaturase enzymes, Δ5 and Δ6 desaturases, respectively, which play critical role in the biosynthesis of long-chain PUFAs [66]. Genetic variation in *FADS1/2* genes has been reported to influence dyslipidemia, inflammatory diseases, T2D, fatty liver disease, cancers, and neurological disorders [67–72]. However, because of complex and controversial findings specifically related to FADS1/2 enzymes, the precise mechanisms by which the therapeutic potential of targeting these enzymes remains unclear. The TMEM258, a transmembrane protein 258 (located within this genetic region), was robustly associated with PC 38:4 (C) and PC(38:5) (A), PC18.2.0_20.4.0/PC38:6 in this study. *TMEM258* has emerged as a potential therapeutic target, particularly in the context of IBD and endoplasmic reticulum stress, and apoptosis [73].

We also identified new mQTLs modulating serum levels of LPCs, particularly the signal for an ether-containing LPC O-16:0 was shown by genetic regions represented by *PLCG2* and *TAFA1* in serum and *ELOVL4* and *GMEB2,* not reported previously. Additionally, 25 new QTL associations emerged for LPC O-16:0, after adjusting for clinical lipids, which included genes like *ROBO2*, *PTPRC*, *TBC1D1*, *TMEM108*, *CDH23*, *CRB1*, and *DENND1B*, etc., with p values ranging from 9.37 × 10^−10^ to 1.50 × 10^−34^. The genetic variance contributed by these mQTLs explained up to 19.1% of the phenotypic variability for LPC O-16:0 in AIDHS/SDS. Associations of SNPs with rare MAFs are generally ancestry-specific because rare variants are more recently shaped by recent demographic events, such as population bottlenecks and genetic drift, thus being restricted within specific populations [22]. In our study, the *PTPRC* (rs558096054) variant associated with LPC O-16:0 has a MAF of 0.007 in the AIDHS/SDS cohort versus MAF of 0.004 in Europeans, which, perhaps because of its rarity, could not be validated in other lipidomics studies.

The population-specific mQTLs for FA18:0;(2OH) have not been documented in any genome-wide studies of lipid metabolites, which showed the highest heritability of 0.78. Interestingly, most of the genes (*ADGRB2*, *PPARGC1α*, *HDAC5*, *PTK7*, and *TRIP4*) associated with FA18:0;(2OH) have also been linked to T2D, obesity, and CAD phenotypes [74,75]. Fine mapping analysis also revealed the top mQTL (rs76495718; *PPARGC1A*) had a PIP = 0.99 and Z-score = −7.08. FA18:0;(2OH) with an alcohol functional group at the second carbon position that is also referred to as dihydroxy stearic acid. The hydroxyl group is introduced through the oxidation of stearic acid. Moreover, this fully saturated FA18:0;(2OH) interacts with *PPAR*s (peroxisome proliferator-activated receptors and *PGC1α*), which regulate inflammation and glucose metabolism. *PGC1α* is a transcription coactivator that enhances the activity of *PPAR*s and other transcription factors. Two well-known diabetes drugs, Pioglitazone (a *PPAR*-gamma agonist) and Metformin, activate *PGC-1α* via the AMPK and cAMP response element-binding (CREB) proteins pathway [76]. It is highly involved in T2D and obesity, mainly through its influence on insulin sensitivity and lipid metabolism. The specific FA18:0;(2OH) mQTL SNPs in *PGC1α* and *PTK7* exhibited a strong association with creatine levels and HbA1c in the UK Biobank.

To determine causal effects of these and other significant mQTLs with cardiometabolic disease with statistical robustness and sensitivity, we employed a two-sample MR approach utilizing data from UKBB and other large-scale GWAS cohorts, some available with lipid metabolite traits. The MR approach can outwit the noise of the reverse causation using suitable gene variants as a genetic instrument and minimize the effects of confounding due to horizontal pleiotropy. According to our study, LPC O-16:0 was identified as a common marker for showing a causal relationship in T2D. The LPC O-16:0 is a type of ether-linked lysophospholipids involved in various physiological and pathological processes, particularly inflammation, cell signaling, and lipid metabolism [77]. Compared to diacyl-LPCs, ether-linked LPCs have a distinct role in influencing vascular inflammation. The oxidized low-density lipoproteins (oxLDL) can produce ether-linked LPCs, which contribute to endothelial dysfunction and plaque formation [78]. Specifically, the LPC O-16:0 is a pro-inflammatory lipid mediator that activates toll-like receptors (TLRs) by recruiting immune cells. Its accumulation can cause macrophage activation and T-cell response [79]. LPC signaling has been linked to demyelination and oxidative stress in the brain and has been shown to cause inflammation in the central nervous system [77,78]. Because LPCs may worsen insulin resistance and adipose tissue inflammation in obesity, T2D, and metabolic syndrome, this may explain the increased risk of atherosclerotic vascular disease associated with LPCs in diabetes. Our study identified *PTPRC* represented by rs558096054, the top QTL for LPC O-16:0, which is a protein tyrosine phosphatase receptor type C, also called CD45, a key regulator of T- and B-cell antigen receptor signaling, and is already used as a biomarker and therapeutic target. Fine mapping analysis also identified a high PIP score (PIP 0.99) for this variant. Monoclonal antibody (anti-CD45) is used to target *PTPRC* in immunology and cancer [80]. While both *PTPRC* and LPC are involved in immune regulation and inflammatory processes, our study has identified the causal association of LPC O-16:0 for increasing the risk for T2D using IVW-MR and colocalization approaches. GSEA confirmed LPC O-16:0 in regulating AMPK signaling, leptin signaling, inflammatory bowel disease, and lipid metabolism through *PGC-1α*. Additionally, the *ENPP1* gene was among the other LPC O-16:0 QTLs identified in this study. *ENPP1* is a drug target that is involved in insulin signaling and nucleotide metabolism. Inhibitors of *ENPP1* are currently in preclinical/clinical development for metabolic diseases, cancer, and calcification disorders [81,82]. Based on this data, LPC O-16:0, through its role in inflammation, may serve as a potential biomarker for the early detection and prevention of T2D. Several mQTLs for LPC-O16:0 detected in this study were co-shared with other diseases reported in earlier GWAS of Asthma, CRP, BMI, HDL-C, TG, CVD, IBD, SBP, T2D, WHR, and ApoA1.

Chromosome 11q12-q13.1 region revealed strong co-sharing of associated genes with multiple lipid species (FAs, PCs, LPCs, PEs, and TGs), and these genes co-localize with other loci linked to various chronic diseases like Crohn’s disease, asthma, rheumatoid arthritis, IBD, and blood levels of ApoB, HDL-C, PCSK9, ApoA1, LDL-C, identified in large GWAS and meta-analysis studies. Additionally, the top QTLs identified for PC 38:4 (C), TG 53:4, CE 20:4, FA 20:4, FA 20:5 in the FADS region revealed robust association with ApoB, CRP, creatinine, glucose, and HbA1c levels in UKBB (p values 5 × 10^−8^ to 2.47 × 10^−89^), which suggests an intricate link between lipid metabolism and cardiometabolic disease and common pathways underlying lipid associated CV and metabolic diseases. The region representing the *FADS1/2*, *MYRF*, and *TMEM258* revealed *cis-*eQTL and chromatin interactions with neighboring genes, suggesting pleiotropy and pinpointing shared biology between multiple traits and disease. Due to extensive pleiotropic interactions in this region, the causal association of PC 38:4 (C) with CAD or T2D could not be confirmed in sensitivity analysis because the directional effects for T2D and CAD with this metabolite were conflicting. Moreover, the association of a single key SNP with multiple metabolomic sub-phenotypes from different lipid classes also made the selection of SNP variants for genetic instruments challenging. PC 38:4 (C) contains palmitic acid and adrenic acid involved in various cellular and disease processes, including inflammation and immune response, atherosclerosis, and Alzheimer’s disease. Reduced levels of PC 38:4 were found in patients with severe coronary calcification compared to those with mild calcification [83]. However, the role of PC 38:4 in CVD is inconsistent and controversial, and more research is needed to understand the precise role of PC 38:4 and other PC species in the development and progression of CVD.

The genome-wide PRS for T2D did not reveal any strongly associated lipid metabolites, whereas the genome-wide PRS for CAD identified several PUFAs to be significantly increased in individuals with high PRS at *p* </= 5 × 10^−8^, both in ancestry-specific and EU-derived PRS scores for CAD (S12b Table). Their *R*^2^ values ranged between 4% to 4.9% in ancestry-specific CAD PRS and 2.4% to 3.1% in EU-derived PRS, which further supports the role of these lipids in T2D and cardiovascular etiologies. It was strange to observe that despite having a strong heritability (with *R*^2^ values ranging between 19% to 37.3%), the metabolite PRS for LPC O-16:0 and PC 38:4 (C) did not provide any strong evidence of the utility of aggregated genetic scores for predicting T2D or CAD than the individual lead SNPs for these lipid markers. Conversely, the aggregated metabolite PRS derived from the top 44 lead SNPs of LPC O-16:0 successfully predicted the risk of developing T2D with increased levels of LPC O-16:0 from 6% (95% CI [2%,9%]; *p* </= 0.004).. Note that including only the high-impact 44 SNPs increased the heritability of LPC O-16:0 from 0.16 to 0.19, compared to the heritability derived from genome-wide SNPs. The motivation for exploring the PRS for only top lipid markers was to validate the individual lead variants and rule out the possibility of other genetic regions affecting the marker, specifically for LPC O-16:0, which has not been reported previously. At the same time, we also sought to identify ancestry-specific differences, if any, using ancestry-specific and European-derived PRS*.* These findings also suggest that the genome-wide metabolite PRS scores are not superior predictors of disease risk when compared to single or top variant mQTLs. Somewhat similar results have been shown for PRS in previous metabolomics studies [43].

There are several strengths and some limitations of this study. This is the first report with detailed genetic characterization of lipid metabolite phenotypes and their role in T2D and CV phenotypes using a mGWAS, MR, and colocalization studies in an Asian Indian population. The role of lipids and lipid-derived metabolites in insulin resistance and low-BMI phenotype of T2D (specific for Asian ancestries from South Asia) is less characterized. By examining a relatively homogenous study cohort collected from a narrow geographic location (Punjab), we reduced heterogeneity confounded by dietary and cultural differences often observed in study cohorts originating from distant geographical locations. These inclusions imparted sufficient power to detect the mQTL effects with decent effect sizes in our study, using the modest size of our Punjabi cohort (*R*^2^ values ranging from ~19% to 37%). Second, to minimize false discoveries, we employed strong QC measures, including adjusting for batch effect, using genomic control PCs, metabolite PCs, and controlling for clinical lipids in the analyses modeling. Third, using other independent studies with lipidomics data created with a similar LC–MS/MS platform, our study successfully validated many earlier known genetic regions associated with lipid mQTLs found in other studies on EUs (*FADS* cluster). Fourth, using rigorous analysis approaches and QC, our study identified new mQTLs regulating LPC O-16:0 and PC 38:4 (C) transecting the pathways linked with T2D, glucose metabolism, CAD, stroke, inflammation, and immuno-vascular diseases. The AIDHS/SDS patients were not receiving lipid-lowering medications during recruitment, resulting in accurate estimates of lipid species and their associated outcomes without using any statistical correction. It is important to note that the strong correlation between lipid metabolites and their interconnected pathways can influence the results. We used lipid PCs to account for the primary source of this variability, which enhances the statistical robustness of the model and helps identify true associations [38]. The weaknesses include the absence of an independent validation cohort of Asian Indians from India. Dietary patterns may modulate blood levels of lipid metabolites, which can in turn influence the ethnic differences and perturb genetic associations through gene-diet or gene-environment interactions. The lipid profiles in serum and plasma are not comparable because they are different biological materials. By utilizing genetic associations and applying Bonferroni correction (FDR of 1.92 × 10^−10^), we identified 36 metabolite-SNP QTLs that exhibited consistent beta direction for the minor allele in both serum and plasma. The present study only reports genetic association for structurally characterized (annotated) lipid metabolite peaks. Future characterization of genetic signals in the unannotated peak regions will be crucial to identify additional new disease pathways. Patients of AIDHS/SDS were ascertained based on T2D and matched non-diabetic controls according to age and ethnicity, which may have influenced genetic associations with lipid metabolites. The currently available genetic data resources for SAs from UKBB do not include lipidomics data, which was a limitation of this study conducted on an underserved population. However, using other external datasets of EU populations from Finland, Australia, Germany, the US, and SAs from Pakistan, we successfully validated mQTLs not reported in EUs while replicating previously reported genetic associations.

In summary, we identified new lipid mQTLs associated with phospholipid subclasses LPC O-16:0 and FA 18:0;(2OH), not reported previously. In addition, we confirmed the involvement of the chromosome 11 region representing *FADS1/2* and *TMEM258* genes for their role in a variety of metabolic and inflammatory diseases, while identifying an ancestry-specific association of PC 38:4 (C) in this region, which may increase CAD risk in SAs. Using two-sample MR, PRS, and colocalization analysis, we identified and confirmed a causal association between LPC O-16:0 with T2D, which may be triggered by activation of AMPK, insulin, and leptin signaling contributing to metabolic diseases, immune system dysfunction, cancer, and inflammation. Ultimately, our study provides strong evidence that more studies on human lipidomics are needed to identify the downstream effects of the genome and how it impacts human cardiometabolic health.

## Supporting information

S1 Fig Bar graph showing the number of lipid metabolites sharing the same genetic locus in serum in AIDHS cohort. The numbers of each lipid class that shares the same genetic locus is tabulated in S20a Table. FA, fatty acids; CAR, carnitines; LPC, lysophosphatidylcholine; LPE, lysophosphatidylethanolamine; PC, phosphatidylcholine; PE, phosphoethanolamine; PI, phosphatidylinositol; Cer, ceramides; GlcCer, glucosylceramide; SM, sphingomyelin; DG, diacylglycerol; TG, triacylglycerol.
(DOCX)

S2 Fig Bar graph showing the number of lipid metabolites sharing the same genetic locus in plasma in AIDHS cohort. The numbers of each lipid class that share the same genetic locus is tabulated in S20b Table. FA, fatty acids; CAR, carnitines; LPC, lysophosphatidylcholine; LPE, lysophosphatidylethanolamine; PC, phosphatidylcholine; PE, phosphoethanolamine; PI, phosphatidylinositol; Cer, ceramides; SM, sphingomyelin; DG, diacylglycerol; TG, triacylglycerol.
(DOCX)

S3 Fig Heat map showing the correlation of clinical traits with metabolites. FAs showed a strong positive correlation with FBG (*p* ranging from 2.7 × 10^−08^ to 1.7 × 10^−26^).Sphingolipids were negatively correlated with TG (*p* ranging from 4.2 × 10^−30^ to 5.0 × 10^−49^). FBG, fasting blood glucose; TG, triglycerides; SYSBP, systolic blood pressure; DYSBP, diastolic blood pressure; FA, fatty acids; LPC, lysophosphatidylcholine; PC, phosphatidylcholine; PE, phosphoethanolamine; Cer, ceramides; GlcCer, glucosylceramide; SM, sphingomyelin.
(DOCX)

S4 Fig Forest plot showing the effect size and confidence intervals of FA 20:4 and PC 38:4 (C) genetic association in AIDHS and PROMIS cohorts. The *p*-value was calculated using multivariate regression.
(DOCX)

S5 Fig Forest plot showing odds ratios (OR) and *p* values of the individual significant SNPs in the *FADS* region for their association with PC38:4 (C) and coronary artery disease (CAD). The *p*-value was calculated using multivariate regression.
(DOCX)

S6 Fig Bar graphs showing differences in βeta-coefficients for mQTLs in (A) Serum and (B) Plasma lipids. (DOCX)

S1 Table **a**: Clinical characteristics of the AIDHS/SDS individuals with lipidomics data. **b**: Clinical characteristics of the AIDHS/SDS individuals. **c**: Clinical characteristics of the UK Biobank individuals.
(XLSX)

S2 Table Association of genetic variants with serum lipid metabolite species in AIDHS/SDS. The *p*-value was calculated using multivariate regression.
(XLSX)

S3 Table Association of genetic variants with plasma lipid metabolite species in AIDHS/SDS. The *p*-value was calculated using multivariate regression.
(XLSX)

S4 Table Association of genetic variants with combined serum and plasma metabolite species in AIDHS/SDS. The *p*-value was calculated using multivariate regression.
(XLSX)

S5 Table Genetic associations of metabolite QTLs in the *FADS* region and their validation in other studies. The *p*-value was calculated using multivariate regression.
(XLSX)

S6 Table **a**: The association of mQTL variants with apolipoprotein B in UK Biobank. **b**: The association of mQTL variants with C-reactive protein in UK Biobank. **c**: The association of mQTL variants with Atherosclerosis in UK Biobank. **d**: The association of mQTL variants with Cerebral infarction in UK Biobank. **e**: The association of mQTL variants with Subarachnoid hemorrhage in UK Biobank. **f**: The association of mQTL variants with creatinine in UK Biobank. **g**: The association of mQTL variants with glucose in UK Biobank. **h**: The association of mQTL variants with glycated hemoglobin (HbA1C) in UK Biobank. The *p*-value was calculated using multivariate regression.
(XLSX)

S7 Table **a**: The LPC O-16:0 mQTL SNPs and their minor allele frequencies in different cohorts. **b**: The PC 38:4 (C) mQTL SNPs and their minor allele frequencies in different cohorts.
(XLSX)

S8 Table **a**: The LPC O-16:0 mQTL SNPs and their association with T2D in South Asians. **b**: The LPC O-16:0 mQTL SNPs and their association with T2D in Europeans. The *p*-value was calculated using multivariate regression.
(XLSX)

S9 Table **a**: The LPC O-16:0 mQTL SNPs and their association with CAD in South Asians. **b**: The LPC O-16:0 mQTL SNPs and their association with CAD in Europeans. The *p*-value was calculated using multivariate regression.
(XLSX)

S10 Table **a**: Horizontal pleiotropy and Cochrane Q-statistics of the effect of LPC O-16:0 associated SNPs on T2D. **b**: Horizontal pleiotropy and Cochrane Q-statistics of the effect of LPC O-16:0-associated SNPs on CAD.
(XLSX)

S11 Table The PC 38:4 (C) mQTL SNPs and their association with CAD. The *p*-value was calculated using multivariate regression.
(XLSX)

S12 Table **a**: Association of lipid metabolites with T2D genome-wide Polygenic Risk Score (PRS) using ancestry-specific (PRS AI) and European-derived (PRS EU) variants. **b**: Association of lipid metabolites with CAD genome-wide Polygenic Risk Score (PRS) using ancestry-specific (PRS AI) and European-derived (PRS EU) variants. The p-value was calculated using regression.
(XLSX)

S13 Table **a**: The association of genome-wide metabolite PRS with lipid metabolites. **b**: The association of genome-wide metabolite PRS with T2D and CAD. The p-value was calculated using regression.
(XLSX)

S14 Table Chromatin interaction mapping analysis on chromosome 1 showing chromatin interactions with the *PTPRC* region and their association with CAD and CAD-related disease traits. (XLSX)

S15 Table Chromatin interaction mapping analysis of chromosome 11 showing chromatin and eQTL interactions with *FADS* region and their association with CAD-related traits. (XLSX)

S16 Table Heritability estimates of top metabolites using gene-wide significant variants. The *p*-value was calculated using regression.
(XLSX)

S17 Table **a**: Fine mapping (PIP and z-scores) of mQTLs associated with LPC O-16:0 in AIDHS cohort. **b**: Fine mapping (PIP and z-scores) of mQTLs associated with PC 38:4 (C) in AIDHS cohort. The *p*-value was calculated using multivariate regression.
(XLSX)

S18 Table **a**: Association of genetic variants with serum lipid metabolite species in AIDHS/SDS. **b**: Association of genetic variants with plasma lipid metabolite species in AIDHS/SDS. The *p*-value was calculated using multivariate regression.
(XLSX)

S19 Table The association of LPC O-16:0 with the *SUGCT* gene region (identified by Cadby and colleagues, 2022) [42]. The *p*-value was calculated using multivariate regression.
(XLSX)

S20 Table **a**: The number of lipid metabolites sharing the same genetic locus in serum in AIDHS cohort. **b**: The number of lipid metabolites sharing the same genetic locus in plasma in the AIDHS cohort.
(XLSX)

S1 File IRB and FWA numbers of the participating institutes. (PDF)

S2 File PRS_PMID 38152657. (PDF)

S3 File PRS_PMID 38658478. (PDF)

S1 STROBE-MR Checklist Strengthening the Reporting of Observational Studies in Epidemiology using Mendelian Randomization (STROBE-MR) Statement. (DOCX)

S1 Text Inclusivity-in-global-research-questionnaire. (DOCX)

## Abbreviations

AIDHS/SDS: Asian Indian Diabetic Heart Study/Sikh Diabetes Study
ApoA1: apolipoprotein A1
ApoB: apolipoprotein B
BMI: body mass index
BP: blood pressure
CABG: coronary artery bypass graft
CAD: coronary artery disease
Cer: ceramides
CVD: cardiovascular disease
DBP: diastolic blood pressure
EU: European
FA: fatty acids
FBG: fasting blood glucose
GSA: Global Screening Arrays
GSEA: gene set enrichment analysis
GWAS: genome-wide association studies
HDL-C: high‐density lipoprotein cholesterol
HWE: Hardy–Weinberg equilibrium
IBD: inflammatory bowel disease
IVW: inverse-variance weighted
LC–MS/MS: liquid chromatography–tandem mass spectrometry
LD: linkage disequilibrium
LDL-C: low‐density lipoprotein cholesterol
LPC: lysophosphatidylcholines
MAF: minor allele frequency
MODY: maturity-onset diabetes of the young
MR: Mendelian randomization
PC: phosphatidylcholines
PC: principal components
PRS: polygenic risk score
QC: quality control
QTL: quantitative trait loci
SA: South Asians
SBP: systolic blood pressure
SM: sphingolipids
T2D: type 2 diabetes
TC: total cholesterol
TG: triglycerides
UKBB: UK Biobank
