# Supplementary material for: Identification of lipid quantitative trait loci linked with cardiometabolic disease in Asian Indians and Europeans: A genome-wide association study and Mendelian randomization
Source: PLoS Med. 2026 Apr 23;23(4):e1005039. doi: 10.1371/journal.pmed.1005039 (PMC13105358; doi:10.1371/journal.pmed.1005039)
Supplement: S2 Fig — The numbers of each lipid class that share the same genetic locus is tabulated in S20b Table. FA, fatty acids; CAR, carnitines; LPC, lysophosphatidylcholine; LPE, lysophosphatidylethanolamine; PC, phosphatidylcholine; PE, phosphoethanolamine; PI, phosphatidylinositol; Cer, ceramides; SM, sphingomyelin; DG, diacylglycerol; TG, triacylglycerol. (DOCX) [file pmed.1005039.s002.docx]

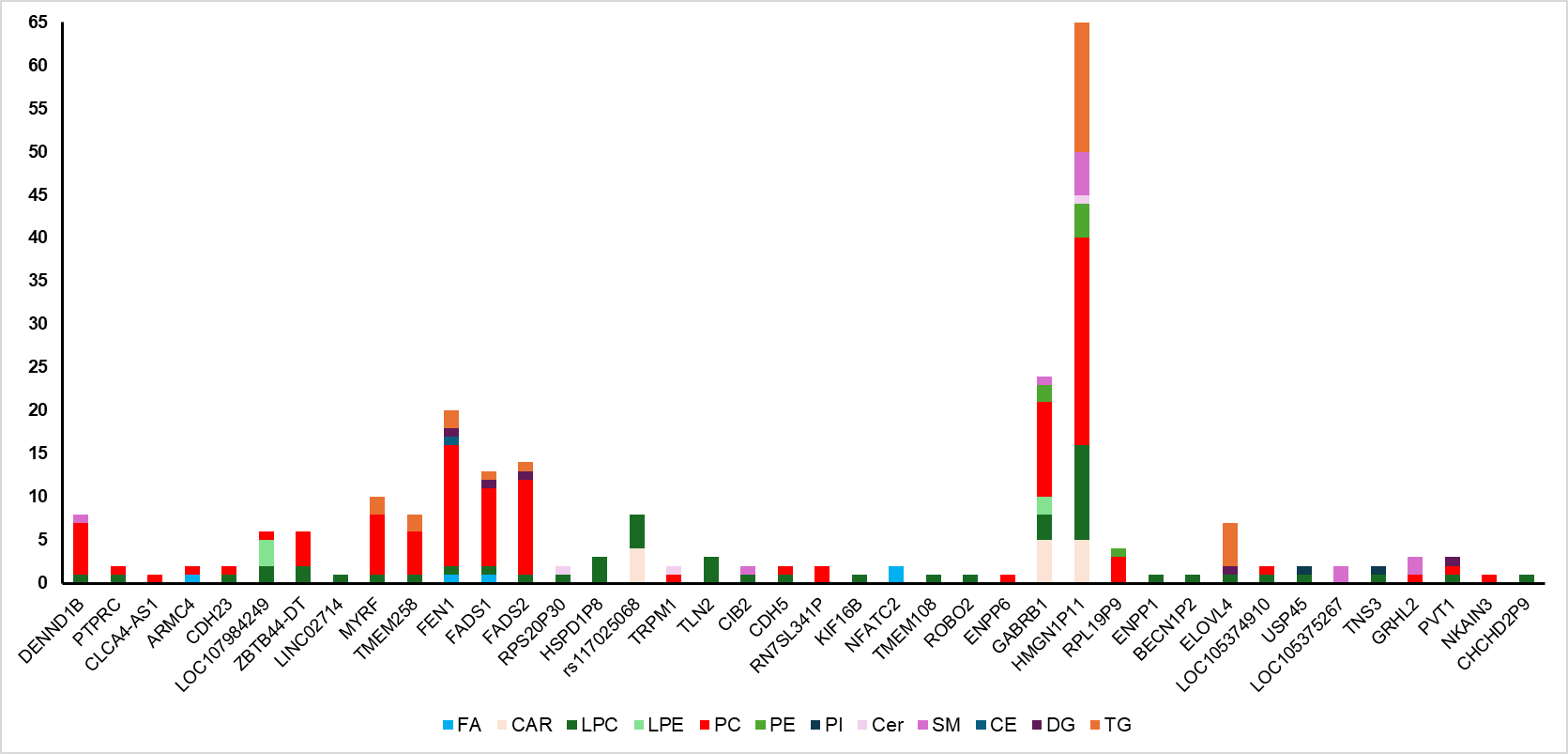


**Supplementary Figure 2**: Bar graph showing the number of lipid metabolites sharing the same genetic locus in plasma in the AIDHS cohort. The number of each lipid class that shares the same genetic locus is tabulated in S20bTable.

FA: fatty acids; CAR: carnitines; LPC: lysophosphatidylcholine; LPE: lysophosphatidylethanolamine; PC: phosphatidylcholine; PE: phosphoethanolamine; PI: phosphatidylinositol; Cer: Ceramides; SM: sphingomyelin; DG: diacylglycerol; TG: triacylglycerol
