# Supplementary material for: Identification of lipid quantitative trait loci linked with cardiometabolic disease in Asian Indians and Europeans: A genome-wide association study and Mendelian randomization
Source: PLoS Med. 2026 Apr 23;23(4):e1005039. doi: 10.1371/journal.pmed.1005039 (PMC13105358; doi:10.1371/journal.pmed.1005039)
Supplement: S3 Fig — FAs showed a strong positive correlation with FBG (p ranging from 2.7 × 10^−08^ to 1.7 × 10^−26^).Sphingolipids were negatively correlated with TG (p ranging from 4.2 × 10^−30^ to 5.0 × 10^−49^). FBG, fasting blood glucose; TG, triglycerides; SYSBP, systolic blood pressure; DYSBP, diastolic blood pressure; FA, fatty acids; LPC, lysophosphatidylcholine; PC, phosphatidylcholine; PE, phosphoethanolamine; Cer, ceramides; GlcCer, glucosylceramide; SM, sphingomyelin. (DOCX) [file pmed.1005039.s003.docx]

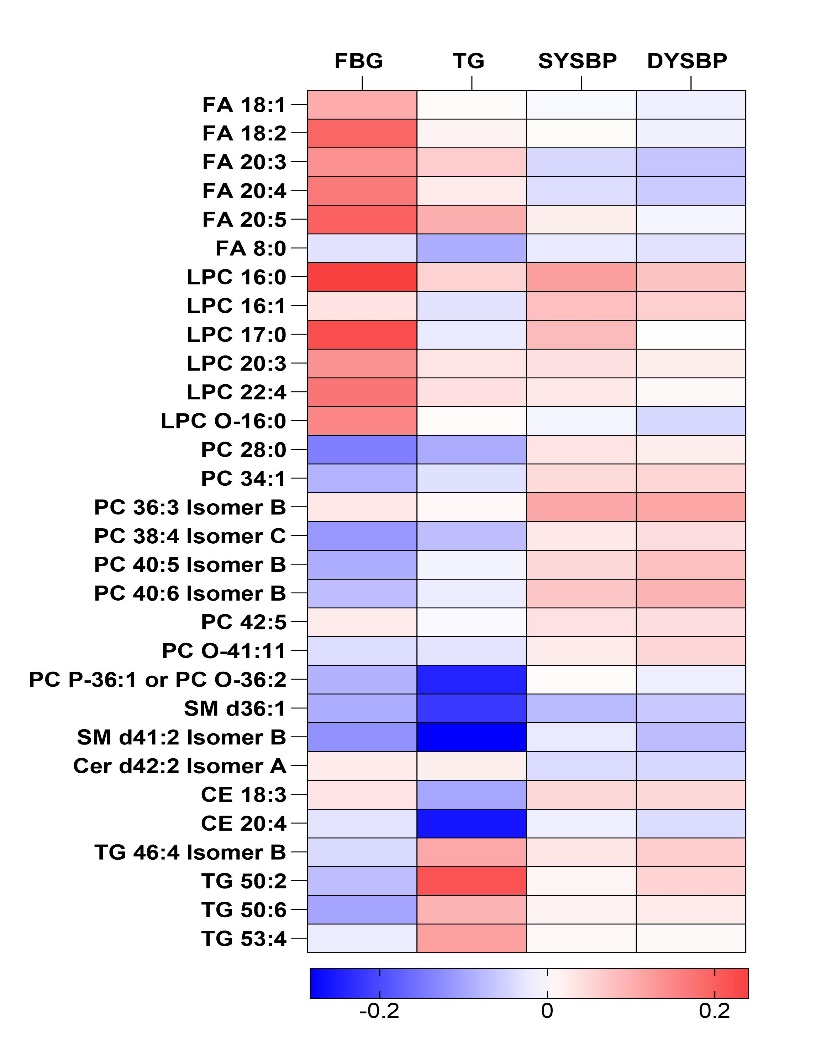


**Supplementary Figure 3:** Heat map showing the correlation of clinical traits with metabolites. FAs showed a strong positive correlation with FBG (p ranging from 2.7x10^-08^ to 1.7x10^-26^).Sphingolipids were negatively correlated with TG (p ranging from 4.2x10^-30^ to 5.0x10^-49^). FBG: Fasting blood glucose; TG: Triglycerides; SYSBP: Systolic blood pressure; DYSBP: Diastolic blood pressure; FA: fatty acids; LPC: lysophosphatidylcholine; PC: phosphatidylcholine; PE: phosphoethanolamine; Cer: Ceramides; GlcCer: glucosylceramide; SM: sphingomyelin
