# Supplementary material for: Identification of lipid quantitative trait loci linked with cardiometabolic disease in Asian Indians and Europeans: A genome-wide association study and Mendelian randomization
Source: PLoS Med. 2026 Apr 23;23(4):e1005039. doi: 10.1371/journal.pmed.1005039 (PMC13105358; doi:10.1371/journal.pmed.1005039)
Supplement: S4 Fig — The p-value was calculated using multivariate regression. (DOCX) [file pmed.1005039.s004.docx]

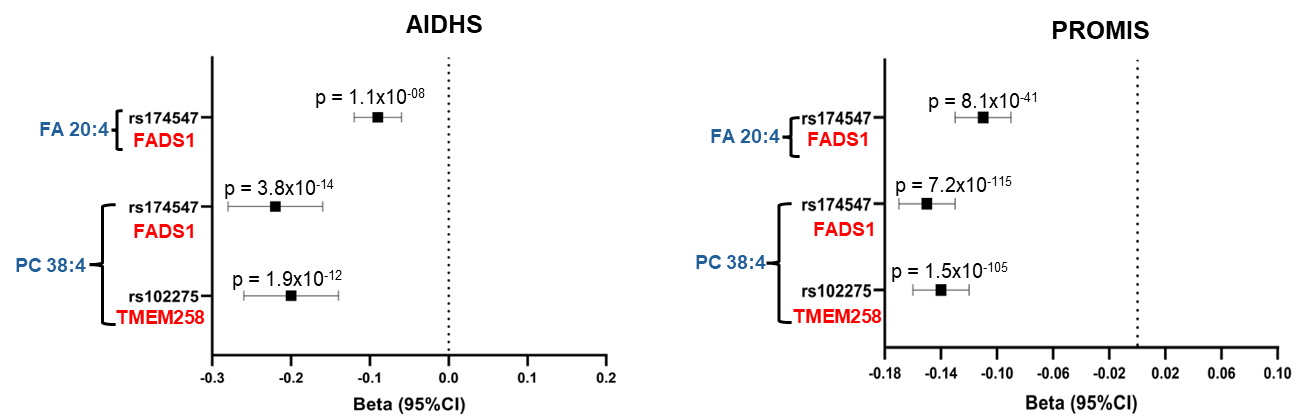


**Supplementary Figure 4**: Forest plot showing the effect size and confidence intervals of FA 20:4 and PC 38:4 (C) genetic association in AIDHS and PROMIS cohorts. The p-value was calculated using multivariate regression.
