# Supplementary material for: Identification of lipid quantitative trait loci linked with cardiometabolic disease in Asian Indians and Europeans: A genome-wide association study and Mendelian randomization
Source: PLoS Med. 2026 Apr 23;23(4):e1005039. doi: 10.1371/journal.pmed.1005039 (PMC13105358; doi:10.1371/journal.pmed.1005039)
Supplement: S5 Fig — The p-value was calculated using multivariate regression. (DOCX) [file pmed.1005039.s005.docx]

**
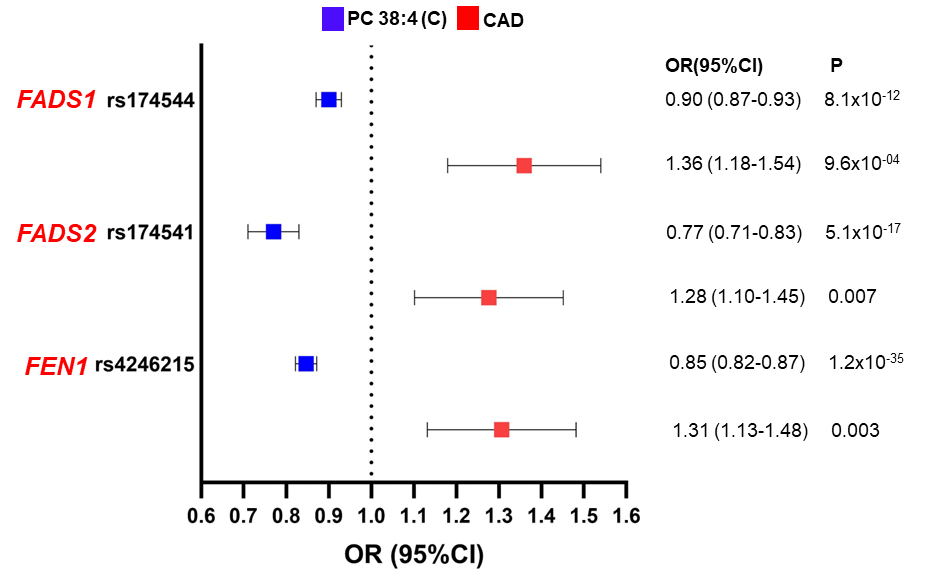
**

**Supplementary Figure 5:** Forest plot showing odds ratios (OR) and p values of the individual significant SNPs in the *FADS* region for their association with PC38:4 (C) and coronary artery disease (CAD). The p-value was calculated using multivariate regression.
