# Supplementary material for: Identification of lipid quantitative trait loci linked with cardiometabolic disease in Asian Indians and Europeans: A genome-wide association study and Mendelian randomization
Source: PLoS Med. 2026 Apr 23;23(4):e1005039. doi: 10.1371/journal.pmed.1005039 (PMC13105358; doi:10.1371/journal.pmed.1005039)
Supplement: S6 Fig — (DOCX) [file pmed.1005039.s006.docx]

**
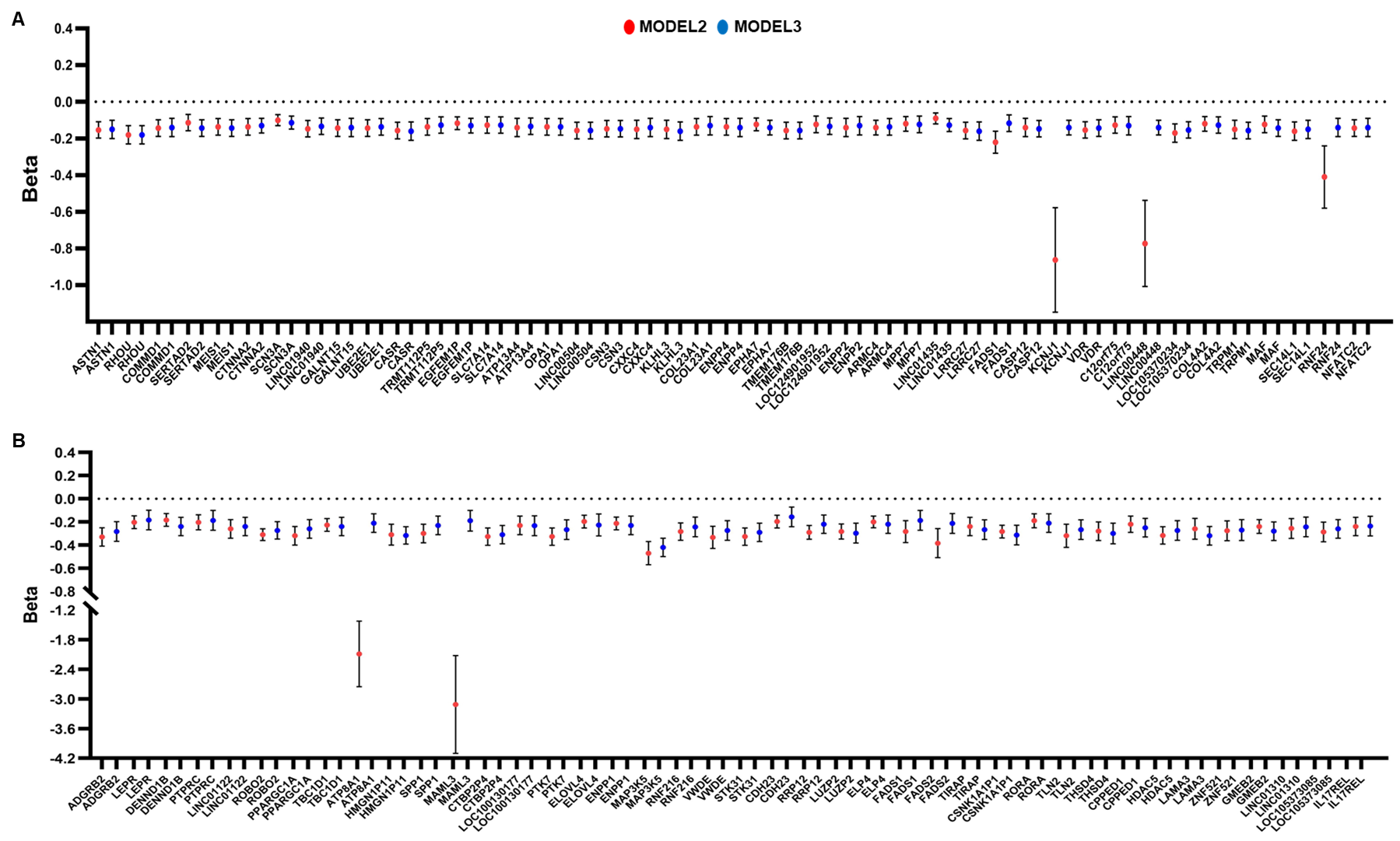
**

**Supplementary Figure 6:** Bar graphs showing differences in βeta-coefficients for mQTLs in (**A**) Serum and (**B**) Plasma lipids
