# Supplementary material for: Identification of lipid quantitative trait loci linked with cardiometabolic disease in Asian Indians and Europeans: A genome-wide association study and Mendelian randomization
Source: PLoS Med. 2026 Apr 23;23(4):e1005039. doi: 10.1371/journal.pmed.1005039 (PMC13105358; doi:10.1371/journal.pmed.1005039)
Supplement: S1 File — (PDF) [file pmed.1005039.s027.pdf]

IRB and FWA Numbers of the Participating Institutes for Sikh Diabetes Study/ Asian Indian Diabetic Heart Study

University of Pittsburgh, PA
IRB Registration # : 0000196
FWA # : 00006790

Hero DMC Heart Institute, Ludhiana, India
International IRB # : 00004177
FWA # : 00006903

Guru Nanak Dev University, Amritsar, India
International IRB # : 00004178
FWA # : 00006995

University of Oklahoma, OK
IRB Registration # IRB00000587/ IRB00000588

FWA # : 00007961
