# Supplementary material for: Identification of lipid quantitative trait loci linked with cardiometabolic disease in Asian Indians and Europeans: A genome-wide association study and Mendelian randomization
Source: PLoS Med. 2026 Apr 23;23(4):e1005039. doi: 10.1371/journal.pmed.1005039 (PMC13105358; doi:10.1371/journal.pmed.1005039)
Supplement: S2 File — (PDF) [file pmed.1005039.s028.pdf]

Supplementary Table 1: The pipeline used for constructing ancestry-derived PRS_{AI} and European PRS_{EU}

PRS_{AI} (**46,985,978 SNPs**)	PRS_{EU} (**33,122,978 SNPs**)
1) Regression adjusted for age, gender, body mass index (BMI), 5 principal components (PCs) in Discovery/Training set (n = 1616)	1) Present in Discovery/Training set (n = 1616) (**23,938,128 SNPs**)<br><br>Regression adjusted for age, gender, BMI, 5 PCs in Discovery/Training set
2) P<0.05 (**896,821 SNPs**)	2) P<0.05 (**725,430 SNPs**)
3)QC (MAF>0.01 to MAF<0.45) and Removing INS, DEL, MIX SNPs	3) QC (MAF>0.01 to MAF<0.45) and Removing INS, DEL, MIX SNPs
4) P<10-4 (**58,267 SNPs**)	4) P<10-4 (**22,602 SNPs**)
5) LD Pruning	5) LD Pruning
6) Rare variants = 1649<br>Common Variants = 1272<br>Total SNPs = **2921**	6) Rare variants = 1029<br>Common Variants = 818<br>Total SNPs = **1847**

Supplementary Table 2: The number of cases and controls distributed by deciles in discovery and validation datasets 1 and 2.

Model	Discovery (AIDHS/SDS) (N=1616)				Validation dataset-1 (AIDHS/SDS) (N=2986)				Validation dataset-2 (UKBB South Asians) (N=9372)			
	PRS_{AI}		PRS_{EU}		PRS_{AI}		PRS_{EU}		PRS_{AI}		PRS_{EU}	
	Cases	Controls	Cases	Controls	Cases	Controls	Cases	Controls	Cases	Controls	Cases	Controls
**D1**	0	162	0	162	4	295	8	291	46	891	45	892
**D2**	0	162	0	162	6	292	23	276	65	872	93	844
**D3**	0	162	0	162	61	237	69	228	82	857	106	831
**D4**	4	158	2	160	115	183	127	169	111	826	144	793
**D5**	42	120	39	123	187	108	194	101	143	794	159	778
**D6**	154	8	158	4	242	54	233	62	170	767	172	765
**D7**	162	0	162	0	282	14	260	38	238	699	216	721
**D8**	162	0	162	0	257	38	249	48	276	661	268	669
**D9**	161	1	162	0	284	14	277	20	318	619	303	634
**D10**	158	0	158	0	293	1	291	3	494	446	437	502

Supplementary Table 3: The PRS range distributed by deciles in discovery and validation datasets 1 and 2.

Model	Discovery (AIDHS/SDS) (N=1616)		Validation dataset-1 (AIDHS/SDS) (N=2986)		Validation dataset-2 (UKBB South Asians) (N=9372)	
	PRS_{AI}	PRS_{EU}	PRS_{AI}	PRS_{EU}	PRS_{AI}	PRS_{EU}
	PRS Range		PRS Range		PRS Range	
**D1**	-202.29 to -86.76	-94.16 to -34.10	-29.25 to -7.71	-21.11 to -5.08	-10.22 to -3.60	-9.35 to -2.77
**D2**	-86.60 to 0.20	-33.94 to -12.13	-7.70 to -4.49	-5.07 to -2.98	-3.59 to -2.45	-2.76 to -1.87
**D3**	0.21 to 9.65	-11.68 to -3.96	-4.48 to -1.91	-2.98 to -1.22	-2.44 to -1.80	-1.86 to -1.26
**D4**	9.83 to 18.43	-3.87 to 4.34	-1.90 to -0.38	-1.22 to -0.38	-1.75 to -1.11	-1.25 to -0.78
**D5**	18.50 to 36.39	4.40 to 25.10	-0.38 to 0.48	-0.37 to 0.17	-1.10 to -0.57	-0.77 to -0.30
**D6**	36.45 to 47.77	25.13 to 36.83	0.49 to 1.32	0.18 to 0.68	-0.56 to 0.01	-0.29 to 0.14
**D7**	47.78 to 56.29	36.84 to 44.65	1.32 to 2.23	0.69 to 1.46	0.02 to 0.63	0.14 to 0.64
**D8**	56.41 to 68.82	44.66 to 55.03	2.23 to 4.70	1.47 to 3.32	0.64 to 1.36	0.64 to 1.21
**D9**	68.94 to 404.17	55.20 to 212.33	4.71 to 9.32	3.33 to 6.70	1.37 to 2.29	1.21 to 1.95
**D10**	405.88 to 922.84	212.52 to 576.43	9.34 to 329.01	6.71 to 105.20	2.30 to 7.25	1.95 to 6.33

Supplementary Table 4: Controls and Cases in 1st and 5th Deciles (discovery and replication datasets)

Model	Discovery (AIDHS/SDS) (N=1616)				Validation dataset-1 (AIDHS/SDS) (N=2986)				Validation dataset-2 (UKBB South Asians) (N=9372)			
	PRS_{AI}		PRS_{EU}		PRS_{AI}		PRS_{EU}		PRS_{AI}		PRS_{EU}	
	Cases	Controls	Cases	Controls	Cases	Controls	Cases	Controls	Cases	Controls	Cases	Controls
**D1**	0	162	0	162	4	295	8	291	46	891	45	892
**D5**	42	120	39	123	187	108	194	101	143	794	159	778

Supplementary Table 5: The number of SNPs and genes associated with lower and upper deciles in Asian Indians and Europeans

Model	Asian Indians		Europeans	
	**SNPs**	**Genes**	**SNPs**	**Genes**
Lower deciles	537	247	346	98
Upper deciles	578	257	414	157
Total	1115	504	760	255
Common	0	58	0	60
Unique	1115	**446**	760	**195**

Supplementary Table 6: The SNPs and genes associated with lower and upper deciles in Asian Indians

			Lower deciles		Upper deciles	
**Predictor**	**Identifier**	**Gene Names**	**Minor Allele**	**Minor Allele Frequency**	**Minor Allele**	**Minor Allele Frequency**
22:44708987-SNV	rs28970381	SHISAL1	C	0.484	G	0.434
6:143880474-SNV	rs867461	PHACTR2-AS1	C	0.341	C	0.291
2:68101063-SNV	rs75726388		T	0.092	T	0.043
19:14862540-SNV	rs372051793	ADGRE2	A	0.053	A	0.003
11:79532067-SNV	rs58986687		T	0.071	T	0.022
1:152997483-SNV	rs11803079		C	0.064	C	0.014
22:17450538-SNV	rs16981911	GAB4	A	0.051	A	0.001
17:75114727-SNV	rs485018	SEC14L1	A	0.257	A	0.207
6:6325739-SNV	rs3844196		C	0.257	C	0.207
18:1818373-SNV	rs193133088		T	0.062	T	0.012
8:18501072-SNV	rs35229445	PSD3	G	0.236	G	0.186
10:123652162-SNV	rs73364463	ATE1	G	0.054	G	0.004
6:135007856-SNV	rs2327527		C	0.385	C	0.335
5:56269512-SNV	rs59536253		G	0.078	G	0.028
1:179769266-SNV	rs73039699	FAM163A	G	0.065	G	0.015
16:16122415-SNV	rs79314289	ABCC1	A	0.052	A	0.002
15:34507832-SNV	rs61054243		C	0.253	C	0.203
4:183797355-SNV	rs11132156		T	0.200	T	0.150
3:88860616-SNV	rs62271543		A	0.153	A	0.102

18:42326974-SNV	rs58306981	SETBP1	C	0.282	C	0.232
17:75535097-SNV	rs4788990		T	0.463	T	0.412
3:122685710-SNV	rs13093469	SEMA5B	T	0.307	T	0.256
20:55713899-SNV	rs13043200		T	0.098	T	0.047
12:71339058-SNV	rs11178498		G	0.370	G	0.320
1:230420601-SNV	rs80323865		T	0.130	T	0.079
18:6440339-SNV	rs7228477		T	0.193	T	0.143
16:4787267-SNV	rs17137214	DNAAF8	G	0.082	G	0.032
11:20157726-SNV	rs7927739		T	0.186	T	0.135
20:42190319-SNV	rs3135414	SGK2	G	0.101	G	0.050
5:109360135-SNV	rs2966775		A	0.405	A	0.355
9:29926323-SNV	rs113864552		A	0.054	A	0.003
5:33589017-SNV	rs4242083	ADAMTS12	A	0.163	A	0.112
3:112434931-SNV	rs7627205		A	0.136	A	0.085
2:205984232-SNV	rs12999205	PARD3B	A	0.155	A	0.104
3:134733516-SNV	rs40347	EPHB1	G	0.179	G	0.128
2:157630580-SNV	rs57672428		C	0.074	C	0.023
6:95275227-SNV	rs75854151		A	0.074	A	0.023
1:4714323-SNV	rs116065312	AJAP1	A	0.145	A	0.094
13:24989765-SNV	rs532735769		C	0.209	C	0.158
2:131045469-SNV	rs11901384		C	0.058	C	0.007
2:154475952-SNV	rs34313210		T	0.116	T	0.065

3:150622735-SNV	rs11717908	MINDY4B	C	0.135	C	0.084
5:129653571-SNV	rs78978431		G	0.064	G	0.012
4:176562144-SNV	rs729721	GPM6A	C	0.082	C	0.031
2:32096569-SNV	rs11901448	MEMO1	C	0.490	C	0.439
9:122039773-SNV	rs7028813	BRINP1	T	0.080	T	0.029
3:116454287-SNV	rs6783957		A	0.149	A	0.098
1:217879151-SNV	rs10863338	SPATA17	A	0.221	A	0.169
12:92437506-SNV	rs12367976	LINC01619	G	0.084	G	0.032
3:196672905-SNV	rs4916588	PIGZ	T	0.160	T	0.109
10:108656776-SNV	rs146371939	SORCS1	C	0.134	C	0.082
17:76555985-SNV	rs11867901	DNAH17	C	0.230	C	0.178
2:198832781-SNV	rs74994281	PLCL1	G	0.060	G	0.009
2:138651160-SNV	rs58175992	LOC101928273	T	0.053	T	0.001
14:77907481-SNV	rs76396354	VIPAS39	C	0.053	C	0.001
1:58933458-SNV	rs11207233		A	0.182	A	0.130
17:6656502-SNV	rs17804432		A	0.395	A	0.342
18:76387285-SNV	rs11081633		A	0.297	A	0.245
16:5408457-SNV	rs7196892		G	0.138	G	0.086
7:147118832-SNV	rs78355037	CNTNAP2	G	0.131	G	0.078
1:51463424-SNV	rs10888713		T	0.102	T	0.050
2:131032203-SNV	rs13398687		T	0.147	T	0.095
5:12379589-SNV	rs79849679		G	0.063	G	0.010

9:100819940-SNV	rs10818454	NANS	A	0.192	A	0.140
11:11657883-SNV	rs11021964		A	0.166	A	0.113
8:92808851-SNV	rs536467629		T	0.282	T	0.230
11:35301324-SNV	rs113142479	SLC1A2	G	0.055	G	0.002
15:92984094-SNV	rs17646850	ST8SIA2	T	0.079	T	0.026
1:7927808-SNV	rs503375		T	0.188	T	0.135
3:37380334-SNV	rs7612650	GOLGA4	T	0.376	T	0.323
18:71493690-SNV	rs9947454		G	0.252	G	0.199
17:78668487-SNV	rs113615547	RPTOR	A	0.054	A	0.001
17:30976624-SNV	rs112524500	MYO1D	T	0.144	T	0.091
7:100738224-SNV	rs28592167	TRIM56	G	0.091	G	0.038
10:106502005-SNV	rs1565423	SORCS3	A	0.292	A	0.239
1:34176366-SNV	rs56277472	CSMD2	A	0.149	A	0.096
19:48477694-SNV	rs113591198	BSPH1	T	0.078	T	0.025
13:53613331-SNV	rs3803259	OLFM4	C	0.493	G	0.440
7:143823833-SNV	rs11763619		C	0.087	C	0.033
9:136075549-SNV	rs11244030		T	0.230	T	0.176
7:130487407-SNV	rs6964345		C	0.151	C	0.097
2:239458607-SNV	rs11895813	LINC01107	T	0.495	C	0.441
3:31121907-SNV	rs73823853		G	0.064	G	0.010
19:1496718-SNV	rs10414511	REEP6	T	0.265	T	0.211
12:92972085-SNV	rs78868712		T	0.408	T	0.354

11:94186238-SNV	rs7128673	MRE11	G	0.302	G	0.248
10:80018020-SNV	rs1720300		C	0.120	C	0.066
11:111264216-SNV	rs7949617		T	0.067	T	0.013
2:80782338-SNV	rs1434059	CTNNA2	T	0.469	T	0.415
1:77547106-SNV	rs199674		T	0.305	T	0.252
2:9381827-SNV	rs2666206	ASAP2	C	0.081	C	0.027
9:83714924-SNV	rs4446802		G	0.346	G	0.292
11:51584835-SNV	rs11488821		A	0.105	A	0.051
8:89698427-SNV	rs7841589		G	0.092	G	0.038
20:15577716-SNV	rs6043384	MACROD2	A	0.066	A	0.012
1:100051969-SNV	rs12118270		T	0.111	T	0.057
3:67168040-SNV	rs560949868		A	0.111	A	0.057
9:121320145-SNV	rs60193421		A	0.175	A	0.120
6:134483116-SNV	rs2758148		A	0.220	A	0.165
6:25823379-SNV	rs2762352	SLC17A1	T	0.062	T	0.007
9:89257632-SNV	rs7854427		C	0.062	C	0.007
7:13894939-SNV	rs7801928		C	0.334	C	0.280
11:78490302-SNV	rs58422047	TENM4	A	0.067	A	0.012
12:115893633-SNV	rs73392274		T	0.059	T	0.005
6:62634314-SNV	rs1931809	KHDRBS2	C	0.274	C	0.219
7:153257417-SNV	rs13245723		A	0.166	A	0.111
5:32480028-SNV	rs111404023		T	0.269	T	0.214

4:81405188-SNV	rs143296487	CFAP299	G	0.066	G	0.011
1:192765377-SNV	rs74130477;rs11305607		A	0.056	A	0.001
2:194336549-SNV	rs73980770		C	0.056	C	0.001
7:153015399-SNV	rs394366		T	0.451	T	0.395
1:50534699-SNV	rs56185427	ELAVL4	C	0.067	C	0.012
6:63168672-SNV	rs592945		G	0.215	G	0.160
6:55629111-SNV	rs7745479	BMP5	T	0.123	T	0.068
12:105180145-SNV	rs2468083		A	0.473	A	0.417
2:216257347-SNV	rs2692230	FN1	C	0.108	C	0.052
6:148314247-SNV	rs3861450		T	0.452	T	0.396
22:50658494-SNV	rs5771241	TUBGCP6	A	0.211	A	0.155
11:133363179-SNV	rs4937781	OPCML	T	0.171	T	0.116
4:41533534-SNV	rs28513452	LIMCH1	C	0.301	C	0.245
21:42527504-SNV	rs880370		A	0.481	A	0.425
4:142947649-SNV	rs1828483	INPP4B	T	0.201	T	0.145
2:56947736-SNV	rs117062642		T	0.127	T	0.071
6:77262183-SNV	rs6925063	LINC02540	A	0.155	A	0.099
7:49728111-SNV	rs76706332		T	0.089	T	0.033
21:32764012-SNV	rs2833393	TIAM1	G	0.277	G	0.220
14:88352680-SNV	rs8014180		C	0.296	C	0.239
3:2969526-SNV	rs3864048	CNTN4	T	0.256	T	0.200

17:12865482-SNV	rs75245742	ARHGAP44	C	0.171	C	0.115
19:22081710-SNV	rs111526263		T	0.275	T	0.218
11:365867-SNV	rs6421980		T	0.248	T	0.192
6:9054497-SNV	rs4521642		G	0.463	G	0.406
2:40601179-SNV	rs981739	SLC8A1	C	0.481	T	0.425
9:35112486-SNV	rs56088457	FAM214B	T	0.299	T	0.242
3:102854133-SNV	rs6789627		G	0.156	G	0.099
3:195919984-SNV	rs3861957		G	0.286	G	0.229
14:30353414-SNV	rs8020469	PRKD1	A	0.101	A	0.044
5:161251644-SNV	rs72817481		T	0.093	T	0.036
8:28923898-SNV	rs936920	KIF13B	T	0.308	T	0.251
20:56040393-SNV	rs4811856		C	0.496	A	0.439
14:38326553-SNV	rs713226		A	0.313	A	0.256
14:77906337-SNV	rs115470193	VIPAS39	C	0.091	C	0.034
19:18505741-SNV	rs8106047	LRRC25	A	0.181	A	0.124
4:8517303-SNV	rs7699438		T	0.240	T	0.182
20:42553993-SNV	rs764673	TOX2	T	0.441	T	0.384
18:805150-SNV	rs3915598	YES1	C	0.459	C	0.402
3:98219089-SNV	rs1461160		T	0.269	T	0.212
20:25176101-SNV	rs4815392	LOC101926889	C	0.327	C	0.270
22:19285596-SNV	rs5993580		C	0.227	C	0.170
13:100911517-SNV	rs114464212	PCCA	G	0.064	G	0.006

5:168204114-SNV	rs7726981	SLIT3	C	0.323	C	0.266
2:105542184-SNV	rs1430088		G	0.498	A	0.440
7:120409658-SNV	rs73425863		A	0.127	A	0.070
5:112935406-SNV	rs4410636		T	0.138	T	0.081
4:32381905-SNV	rs72622747		T	0.163	T	0.105
8:97577207-SNV	rs12156240	SDC2	G	0.305	G	0.248
11:120074732-SNV	rs1287392		T	0.324	T	0.266
9:131670457-SNV	rs3750320	LRRC8A	C	0.298	C	0.240
7:128359751-SNV	rs571531223	FAM71F1	C	0.459	C	0.401
21:11180246-SNV	rs1029219		C	0.230	C	0.172
16:62542245-SNV	rs9936971		G	0.156	G	0.098
3:14581594-SNV	rs6442461	GRIP2	G	0.400	G	0.342
2:106419117-SNV	rs4851091	NCK2	T	0.405	T	0.347
3:40202194-SNV	rs2371143	MYRIP	A	0.236	A	0.178
11:13282979-SNV	rs10741614		C	0.093	C	0.035
6:33911135-SNV	rs2499739		T	0.165	T	0.106
4:39111211-SNV	rs11096979	KLHL5	A	0.303	A	0.245
2:20636133-SNV	rs342101		C	0.160	C	0.102
11:120078393-SNV	rs10790370		A	0.393	A	0.335
11:35271731-SNV	rs61882800		T	0.095	T	0.036
6:24951199-SNV	rs647297	RIPOR2	G	0.211	G	0.152
1:7179058-SNV	rs6689977	CAMTA1	G	0.378	G	0.319

4:25101397-SNV	rs6819784		T	0.347	T	0.288
3:109644325-SNV	rs607043		G	0.379	G	0.320
10:81919800-SNV	rs2784773	ANXA11	T	0.229	T	0.169
17:78406371-SNV	rs6565689	ENDOV	C	0.390	C	0.331
20:41772739-SNV	rs7262310	PTPRT	T	0.136	T	0.077
7:57116560-SNV	rs142960346		A	0.422	A	0.363
8:142202847-SNV	rs28446884	DENND3	A	0.121	A	0.061
7:105693349-SNV	rs6956061		A	0.121	A	0.061
6:109730848-SNV	rs4428538	PPIL6	T	0.192	T	0.133
8:1136087-SNV	rs62489002	DLGAP2	A	0.153	A	0.093
15:89361859-SNV	rs8033375	ACAN	C	0.230	C	0.170
10:3928528-SNV	rs7903926		G	0.359	G	0.300
13:61473511-SNV	rs79732044		C	0.074	C	0.014
1:81336493-SNV	rs10874179		G	0.477	A	0.417
17:42680216-SNV	rs9901510		G	0.271	G	0.211
8:28092874-SNV	rs36015978		G	0.198	G	0.137
3:125841370-SNV	rs57896987	ALDH1L1	C	0.113	C	0.053
3:114824513-SNV	rs10934281	ZBTB20	T	0.087	T	0.026
9:75113148-SNV	rs7039856		C	0.060		0.000
13:81126782-SNV	rs10162023		C	0.060		0.000
8:115347110-SNV	rs1553035		C	0.487	C	0.426
3:137211343-SNV	rs57016563		C	0.122	C	0.061

18:23690701-SNV	rs687193		T	0.252	T	0.191
1:190952175-SNV	rs111205663		C	0.381	C	0.321
7:8422093-SNV	rs17403463		C	0.141	C	0.080
13:74323682-SNV	rs28433830	KLF12	C	0.199	C	0.138
1:76222644-SNV	rs1694422	ACADM	G	0.125	G	0.064
20:55445384-SNV	rs13040152		C	0.229	C	0.168
6:123522777-SNV	rs72613283		T	0.215	T	0.155
1:185567987-SNV	rs1407270	LINC01350	T	0.416	T	0.356
11:7686160-SNV	rs10160547	CYB5R2	T	0.221	T	0.160
4:136235427-SNV	rs10026656		A	0.102	A	0.041
8:134211526-SNV	rs2929986	CCN4	T	0.484	T	0.422
16:87210174-SNV	rs12924174		C	0.301	C	0.240
11:29977241-SNV	rs11030909		A	0.222	A	0.161
1:17404950-SNV	rs10887995	PADI2	G	0.397	G	0.335
8:68918505-SNV	rs11991163	PREX2	G	0.397	G	0.335
6:70248464-SNV	rs6920218		A	0.111	A	0.050
8:122730564-SNV	rs724513		A	0.096	A	0.034
2:228518266-SNV	rs4296418		G	0.471	G	0.410
20:47177473-SNV	rs149105797		A	0.101	A	0.040
14:33212320-SNV	rs28815022	AKAP6	T	0.490	T	0.429
17:77531711-SNV	rs12944984		G	0.125	G	0.064
3:53632113-SNV	rs729839	CACNA1D	A	0.242	A	0.180

13:24913678-SNV	rs9507308		A	0.253	A	0.191
7:128364080-SNV	rs339072	FAM71F1	G	0.303	G	0.241
13:110250280-SNV	rs28390182		G	0.160	G	0.099
15:34872591-SNV	rs74205237	GOLGA8B	C	0.224	C	0.162
1:155083294-SNV	rs72702288		G	0.124	G	0.062
11:2299990-SNV	rs10831683		C	0.331	C	0.269
1:247937019-SNV	rs12747565		T	0.259	T	0.197
16:80011359-SNV	rs4417561		C	0.349	C	0.287
13:76110679-SNV	rs56084319	COMMD6	G	0.193	G	0.131
10:1916190-SNV	rs7098110		T	0.413	T	0.351
12:124143546-SNV	rs7303409	GTF2H3	G	0.133	G	0.071
2:196643395-SNV	rs569530316	DNAH7	C	0.281	C	0.219
11:1907610-SNV	rs112907808	LSP1	C	0.398	C	0.335
15:97158236-SNV	rs1521572		A	0.443	A	0.380
20:18863627-SNV	rs61602234		G	0.229	G	0.166
16:69664919-SNV	rs13337236	NFAT5	A	0.358	A	0.296
3:58219375-SNV	rs79586875		C	0.163	C	0.100
3:2519956-SNV	rs11129155	CNTN4	T	0.322	T	0.259
4:38908616-SNV	rs17583068	FAM114A1	G	0.497	G	0.434
2:109305329-SNV	rs2438921		T	0.137	T	0.075
17:39382557-SNV	rs9902692	KRTAP9-2	T	0.124	T	0.061
11:14936943-SNV	rs12416759		A	0.156	A	0.093

1:235443504-SNV	rs16832530	ARID4B	C	0.064	C	0.001
14:25834447-SNV	rs187851376		T	0.107	T	0.043
18:53975605-SNV	rs1380346		T	0.493	T	0.430
12:133032875-SNV	rs10781628		G	0.354	G	0.290
7:25905599-SNV	rs10235225		G	0.315	G	0.252
5:157837404-SNV	rs72492483	LINC02227	A	0.107	A	0.043
1:167759419-SNV	rs11807657	MPZL1	G	0.165	G	0.101
2:11302381-SNV	rs76262581	SLC66A3	G	0.091	G	0.027
5:108084178-SNV	rs3828655	FER	T	0.163	T	0.099
5:120147499-SNV	rs10072469		A	0.493	C	0.429
2:147728767-SNV	rs75179550		A	0.200	A	0.136
9:270321-SNV	rs17720310	DOCK8	G	0.174	G	0.109
2:34773131-SNV	rs10189096		C	0.282	C	0.218
3:64470292-SNV	rs76715463		T	0.068	T	0.004
15:61265306-SNV	rs12593927	RORA	G	0.489	C	0.425
6:112085431-SNV	rs202099841	FYN	G	0.119	G	0.054
5:108562405-SNV	rs34470299		C	0.463	C	0.398
20:39270524-SNV	rs61137306		T	0.092	T	0.028
19:58171059-SNV	rs2011144		C	0.384	C	0.319
7:25600891-SNV	rs11769634		G	0.363	G	0.298
8:21484457-SNV	rs1480997		A	0.154	A	0.089
6:29850684-SNV	rs2734957		G	0.297	G	0.232

9:3832812-SNV	rs9644834	GLIS3	T	0.212	T	0.148
7:19798870-SNV	rs61178120	TMEM196	C	0.078	C	0.013
12:22159336-SNV	rs4133074		A	0.240	A	0.175
17:4671566-SNV	rs8080014		T	0.486	C	0.421
3:153096465-SNV	rs111290807		T	0.367	T	0.302
4:32434212-SNV	rs575368561		A	0.068	A	0.003
17:78714760-SNV	rs35434628	RPTOR	T	0.090	T	0.025
16:71552741-SNV	rs9935288	LOC105371335	C	0.167	C	0.102
4:70400439-SNV	rs10028725		C	0.437	C	0.372
10:82038779-SNV	rs4934028	MAT1A	G	0.462	G	0.396
4:181354281-SNV	rs4627905		G	0.369	G	0.304
9:136560771-SNV	rs2502738	SARDH	T	0.343	T	0.277
11:63157412-SNV	rs526686	SLC22A9	A	0.431	A	0.365
9:137652615-SNV	rs12000090	COL5A1	G	0.293	G	0.227
3:190539152-SNV	rs1501599		T	0.209	T	0.143
7:128342209-SNV	rs61544090		C	0.291	C	0.225
1:243637748-SNV	rs2994323	SDCCAG8	A	0.349	A	0.283
8:15228556-SNV	rs62501673		T	0.238	T	0.172
2:57751815-SNV	rs75237784		C	0.114	C	0.048
5:38723422-SNV	rs6883199	OSMR-AS1	G	0.236	G	0.170
7:61737044-SNV	rs57100574		C	0.353	C	0.286
17:4952934-SNV	rs389855		A	0.195	A	0.128

21:15966421-SNV	rs460852	LOC388813,SAMSN1-AS1	T	0.324	T	0.258
10:10797288-SNV	rs7894758	CELF2	C	0.499	A	0.432
9:75087741-SNV	rs11143307		G	0.102	G	0.036
11:44383668-SNV	rs73545816		G	0.081	G	0.015
9:73366481-SNV	rs11142567	TRPM3	A	0.203	A	0.137
3:187854201-SNV	rs237662		G	0.248	G	0.182
16:5274623-SNV	rs8059035		A	0.477	A	0.410
10:15400865-SNV	rs45479998	FAM171A1	C	0.086	C	0.019
7:62021693-SNV	rs10950104		A	0.104	A	0.037
17:12923987-SNV	rs8077923		G	0.230	G	0.162
7:24078597-SNV	rs9639468		T	0.220	T	0.152
1:213834643-SNV	rs4655303		T	0.395	T	0.327
8:3594438-SNV	rs2720820	CSMD1	C	0.315	C	0.248
8:102684829-SNV	rs146103162		A	0.244	A	0.176
10:134303665-SNV	rs28437050		T	0.107	T	0.039
8:113371942-SNV	rs1492676	CSMD3	C	0.287	C	0.219
7:156751073-SNV	rs80067883	NOM1	T	0.166	T	0.098
8:123865573-SNV	rs75320558	ZHX2	G	0.224	G	0.156
8:10484604-SNV	rs10102098	RP1L1	G	0.153	G	0.085
3:114815659-SNV	rs80264330	ZBTB20	G	0.119	G	0.050
18:13184825-SNV	rs12961288		A	0.071	A	0.003
3:187866554-SNV	rs237663		C	0.381	C	0.313

11:87330974-SNV	rs28748043		A	0.212	A	0.144
6:132940293-SNV	rs4451148	TAAR2	T	0.405	T	0.337
10:89779324-SNV	rs2673824		C	0.405	C	0.337
11:57116707-SNV	rs2003065	P2RX3	C	0.170	C	0.102
12:17026908-SNV	rs181883606		T	0.073	T	0.004
6:166917937-SNV	rs2071940	RPS6KA2	C	0.123	C	0.054
7:7795679-SNV	rs13234192	UMAD1	A	0.470	A	0.401
14:24115521-SNV	rs35270753	DHRS2	T	0.256	T	0.187
6:14576566-SNV	rs61413382		G	0.092	G	0.023
12:103561692-SNV	rs5800568	LOC101929058	G	0.167	G	0.098
4:94367364-SNV	rs11097364	GRID2	A	0.427	A	0.358
16:78863998-SNV	rs61185547	WWOX	C	0.264	C	0.194
5:79836192-SNV	rs32855	FAM151B	G	0.322	G	0.252
2:64745773-SNV	rs80069828		C	0.098	C	0.028
13:67130889-SNV	rs17081523	PCDH9	C	0.071	C	0.002
3:189303939-SNV	rs1920246		C	0.207	C	0.137
10:95747526-SNV	rs116366905		C	0.082	C	0.012
6:3406785-SNV	rs62391777	SLC22A23	T	0.270	T	0.200
8:136231301-SNV	rs12114816		G	0.146	G	0.076
5:61988803-SNV	rs4700015		A	0.493	G	0.423
12:49262834-SNV	rs12581864		A	0.195	A	0.124
12:42824484-SNV	rs1839935	PPHLN1	C	0.486	T	0.415

2:181158448-SNV	rs4893904		T	0.076	T	0.005
21:11040938-SNV	rs2537266	BAGE2	C	0.380	C	0.310
1:239857992-SNV	rs4145784	CHRM3	G	0.126	G	0.056
10:6534728-SNV	rs647934	PRKCQ	G	0.164	G	0.093
7:24467256-SNV	rs10278322		C	0.182	C	0.112
9:12340116-SNV	rs7036725		T	0.156	T	0.085
10:101677488-SNV	rs12780060	DNMBP	T	0.395	T	0.324
16:78096226-SNV	rs2344492		T	0.159	T	0.089
6:11230402-SNV	rs1475345	NEDD9	G	0.377	G	0.306
5:148826915-SNV	rs353244		C	0.491	C	0.420
11:21532918-SNV	rs4074587	NELL1	A	0.251	A	0.179
3:36801218-SNV	rs1396032		G	0.465	G	0.394
22:44704797-SNV	rs11347258	SHISAL1	G	0.452	G	0.380
7:27512437-SNV	rs9639550		T	0.380	T	0.309
1:193039381-SNV	rs10921307	RO60	G	0.171	G	0.100
1:190980629-SNV	rs61818104		A	0.132	A	0.061
14:90304702-SNV	rs8006866	EFCAB11	A	0.476	A	0.405
5:39261435-SNV	rs181799	FYB1	C	0.423	C	0.352
18:74937461-SNV	rs11151012		T	0.220	T	0.148
4:118452507-SNV	rs10008335	LINC01378	C	0.109	C	0.037
6:25836257-SNV	rs1165184		G	0.173	G	0.101
19:14847661-SNV	rs183122826	ADGRE2	C	0.146	C	0.075

11:24698345-SNV	rs147999980	LUZP2	T	0.073	T	0.001
8:81158941-SNV	rs73257743		T	0.168	T	0.096
16:4766993-SNV	rs8059549	ANKS3	G	0.105	G	0.033
8:134122237-SNV	rs6984052	TG	G	0.222	G	0.150
10:76107179-SNV	rs10824154	ADK	C	0.408	C	0.335
8:29397736-SNV	rs10113675		T	0.368	T	0.296
4:168104162-SNV	rs4859973	SPOCK3	A	0.485	A	0.412
16:90011255-SNV	rs13338472		C	0.140	C	0.067
6:89015989-SNV	rs1408703		A	0.291	A	0.218
6:27573536-SNV	rs6456799	TRL-CAA2-1	A	0.499	A	0.425
5:110979158-SNV	rs1379552	STARD4-AS1	C	0.285	C	0.211
1:13709340-SNV	rs61781185		T	0.330	T	0.256
20:53874547-SNV	rs2426559		A	0.407	A	0.333
6:35912918-SNV	rs9470177	SLC26A8	C	0.185	C	0.111
2:70830411-SNV	rs11678699		C	0.287	C	0.213
12:133164584-SNV	rs57470468		C	0.319	C	0.245
19:22443261-SNV	rs2023338		T	0.279	T	0.205
16:84422162-SNV	rs4782946	ATP2C2	T	0.479	T	0.405
14:90313466-SNV	rs61998707	EFCAB11	C	0.297	C	0.222
17:77637659-SNV	rs1507710		G	0.236	G	0.161
9:14457483-SNV	rs149135564	NFIB	A	0.223	A	0.148
10:59333310-SNV	rs6481297		T	0.462	T	0.387

8:14921828-SNV	rs9657250	SGCZ	T	0.319	T	0.244
4:37922307-SNV	rs2925957	TBC1D1	G	0.493	C	0.418
11:21748082-SNV	rs77665513		C	0.076	C	0.001
2:240571827-SNV	rs4852100		G	0.386	G	0.311
10:15369706-SNV	rs1955343	FAM171A1	A	0.280	A	0.205
1:203606490-SNV	rs1970962	ATP2B4	G	0.397	G	0.321
18:828613-SNV	rs145462650		C	0.395	C	0.319
5:61962291-SNV	rs12152765		C	0.426	C	0.351
16:58325623-SNV	rs72786177	PRSS54	A	0.173	A	0.097
9:7000769-SNV	rs2792238	KDM4C	C	0.334	C	0.259
7:77412489-SNV	rs3944103	RSBN1L	C	0.195	C	0.119
13:114840932-SNV	rs61973926	RASA3	G	0.322	G	0.246
5:79680373-SNV	rs12655561		T	0.312	T	0.236
1:200188235-SNV	rs10919852		G	0.376	G	0.300
17:36117058-SNV	rs12603084		G	0.360	G	0.284
13:111744192-SNV	rs4773318		G	0.084	G	0.007
8:146063308-SNV	rs2722491	ZNF7	G	0.102	G	0.026
14:47644925-SNV	rs146261046	MDGA2	C	0.354	C	0.277
11:62852211-SNV	rs61285056	SLC22A24	T	0.105	T	0.029
22:40063424-SNV	rs136856	CACNA1I	C	0.429	C	0.352
18:10454279-SNV	rs77168889	APCDD1	A	0.265	A	0.188
3:157290688-SNV	rs12696049	SLC66A1L	A	0.238	A	0.161

11:105369119-SNV	rs10895827		C	0.284	C	0.207
5:66979313-SNV	rs146030645		T	0.080	T	0.003
14:90922304-SNV	rs12433543	LINC00642	T	0.396	T	0.318
1:112371990-SNV	rs78027663	KCND3	G	0.134	G	0.057
10:116719543-SNV	rs7099565	TRUB1	G	0.484	A	0.406
5:143062910-SNV	rs60265992		C	0.201	C	0.123
8:10485154-SNV	rs6986884	RP1L1	G	0.175	G	0.097
14:90902728-SNV	rs4904692		A	0.498	A	0.420
12:5843821-SNV	rs7310966	ANO2	C	0.469	C	0.391
9:4354854-SNV	rs7467861		T	0.463	T	0.384
9:138340971-SNV	rs10858110		C	0.423	C	0.345
4:132407583-SNV	rs1280701		G	0.474	G	0.395
3:2869449-SNV	rs6793237	CNTN4	G	0.342	G	0.263
5:174074955-SNV	rs6888000		C	0.313	C	0.234
11:111263745-SNV	rs17112736		T	0.129	T	0.050
13:92149048-SNV	rs553945	GPC5	G	0.438	G	0.359
18:33291393-SNV	rs1573367	GALNT1	G	0.124	G	0.045
8:16175982-SNV	rs1366956		G	0.308	G	0.228
12:10876805-SNV	rs11053915	YBX3	G	0.496	C	0.416
5:164600444-SNV	rs28532271		C	0.377	C	0.297
13:45413500-SNV	rs11147957		A	0.473	A	0.393
4:144285200-SNV	rs28989215	GAB1	A	0.120	A	0.040

6:45738031-SNV	rs9395116		G	0.379	G	0.299
13:73131694-SNV	rs17245822		C	0.353	C	0.273
9:11957111-SNV	rs62551962		A	0.488	A	0.408
14:21062542-SNV	rs61995622	LOC254028	T	0.396	T	0.315
11:111012458-SNV	rs1356412		T	0.485	T	0.404
1:34308305-SNV	rs6682867	CSMD2	A	0.090	A	0.009
8:10481016-SNV	rs75015898	RP1L1	C	0.313	C	0.231
2:80778639-SNV	rs12479376	CTNNA2	G	0.247	G	0.165
5:5539266-SNV	rs17770324		A	0.474	A	0.391
3:34543046-SNV	rs4678690		A	0.390	A	0.307
2:169586651-SNV	rs6738607	CERS6	G	0.108	G	0.025
12:53743041-SNV	rs140016012		A	0.153	A	0.070
5:35868538-SNV	rs75399741	IL7R	C	0.140	C	0.057
17:22057069-SNV	rs73982952		G	0.124	G	0.041
12:71614739-SNV	rs28463310		C	0.477	C	0.394
9:101053587-SNV	rs1435254	GABBR2	G	0.464	G	0.380
18:10097536-SNV	rs72959474		T	0.255	T	0.172
2:14750547-SNV	rs35806587		A	0.300	A	0.217
7:56235504-SNV	rs201710419		C	0.488	C	0.405
5:89409314-SNV	rs173952		T	0.399	T	0.315
17:42720107-SNV	rs75868405		G	0.256	G	0.172
10:26458452-SNV	rs11014982	MYO3A	A	0.111	A	0.027

5:56275949-SNV	rs7719846		A	0.085	A	0.001
9:90267803-SNV	rs10780868	DAPK1	G	0.5	C	0.416
4:140915940-SNV	rs13148756	MAML3	T	0.487	T	0.403
17:67395222-SNV	rs2592204		A	0.300	A	0.216
4:125791733-SNV	rs11730077		A	0.116	A	0.032
7:105734508-SNV	rs1024561	SYPL1	A	0.268	A	0.183
8:19670881-SNV	rs13258148		A	0.202	A	0.117
19:5367684-SNV	rs11878760		C	0.468	C	0.383
14:59950013-SNV	rs10147234	L3HYPDH	A	0.429	A	0.343
6:151115550-SNV	rs62432670	PLEKHG1	T	0.259	T	0.174
1:220523521-SNV	rs9803782		G	0.376	G	0.290
4:43330795-SNV	rs10027757		A	0.291	A	0.206
1:190979336-SNV	rs7543180		G	0.492	A	0.407
21:14704126-SNV	rs3117265		T	0.448	T	0.363
17:70843544-SNV	rs1807802	SLC39A11	A	0.240	A	0.154
18:35304062-SNV	rs12606007		G	0.367	G	0.281
11:80592567-SNV	rs10897692		G	0.438	G	0.352
12:42619524-SNV	rs34289722	YAF2	A	0.310	A	0.224
1:37899315-SNV	rs35537303		A	0.340	A	0.253
22:49338289-SNV	rs17177978		C	0.384	C	0.297
6:3410247-SNV	rs2277076	SLC22A23	C	0.455	C	0.367
19:9548698-SNV	rs55661628		C	0.455	C	0.367

4:144216957-SNV	rs60442595		T	0.090	T	0.002
20:2629188-SNV	rs6050878		G	0.218	G	0.130
5:20041233-SNV	rs1369759	CDH18	A	0.264	A	0.175
22:49759260-SNV	rs2007314		T	0.434	T	0.345
13:22182573-SNV	rs60074257		A	0.424	A	0.335
12:76232239-SNV	rs7970739		A	0.309	A	0.220
6:109728441-SNV	rs9480949	PPIL6	C	0.497	C	0.408
11:111274876-SNV	rs7121760		G	0.302	G	0.213
16:90007590-SNV	rs4785749		C	0.499	C	0.409
5:73376219-SNV	rs1818123		T	0.446	T	0.356
12:111719060-SNV	rs6490042	CUX2	A	0.465	A	0.375
4:136304810-SNV	rs6833472		T	0.092	T	0.002
1:199167839-SNV	rs10800614	LINC02789	T	0.440	T	0.349
16:64099629-SNV	rs17396307		A	0.493	T	0.402
4:45763629-SNV	rs10805139		A	0.438	A	0.347
4:8527968-SNV	rs3103079		C	0.471	C	0.380
20:2619879-SNV	rs6515677	TMC2	A	0.244	A	0.152
16:4784040-SNV	rs8049395	ANKS3	C	0.113	C	0.020
5:160173130-SNV	rs7737399	ATP10B	G	0.207	G	0.113
5:160168196-SNV	rs17058180	ATP10B	C	0.121	C	0.027
2:36381495-SNV	rs1730586		C	0.481	C	0.387
7:125447381-SNV	rs7804031		G	0.130	G	0.036

3:1499836-SNV	rs1979157		C	0.360	C	0.266
10:130433590-SNV	rs1999048		T	0.379	T	0.285
7:122774603-SNV	rs2470983	SLC13A1	A	0.284	A	0.189
1:94496446-SNV	rs56253197	ABCA4	C	0.168	C	0.073
5:49918197-SNV	rs111800549		G	0.466	G	0.370
6:9066121-SNV	rs9505600		A	0.345	A	0.249
7:122778478-SNV	rs2462143	SLC13A1	A	0.097	A	0.001
14:77930592-SNV	rs11624190	AHSA1	C	0.433	C	0.337
3:125399463-SNV	rs2976710		T	0.331	T	0.234
14:81319796-SNV	rs10129919	CEP128	C	0.219	C	0.122
6:64467096-SNV	rs35734316	EYS	G	0.140	G	0.043
14:36558266-SNV	rs7145211	LINC00609	T	0.378	T	0.281
10:36472589-SNV	rs55675860		C	0.151	C	0.054
7:102232303-SNV	rs200850414	RASA4	T	0.486	T	0.387
4:144218374-SNV	rs11941277		G	0.274	G	0.175
1:18812316-SNV	rs708088	KLHDC7A	T	0.409	T	0.310
5:22255211-SNV	rs7714242	CDH12	T	0.158	T	0.058
10:134328867-SNV	rs4880406	LOC107984282	G	0.199	G	0.099
1:112375325-SNV	rs6663764	KCND3	C	0.249	C	0.149
13:100912738-SNV	rs9557403	PCCA	C	0.470	C	0.370
9:89258560-SNV	rs13300698		C	0.253	C	0.151
5:129213060-SNV	rs244454		T	0.499	T	0.398

14:41151226-SNV	rs55831256		C	0.214	C	0.113
1:147379609-SNV	rs28498866	GJA8	T	0.208	T	0.106
1:147831280-SNV	rs502668		C	0.244	C	0.141
5:12376328-SNV	rs7701646		A	0.351	A	0.248
20:50301563-SNV	rs71338430	ATP9A	T	0.429	T	0.325
10:134325696-SNV	rs114493219	LOC107984282	C	0.131	C	0.027
8:1131480-SNV	rs7818243	DLGAP2	C	0.359	C	0.255
11:111282629-SNV	rs11213878		A	0.344	A	0.240
6:3973673-SNV	rs6597020		C	0.171	C	0.066
7:25747652-SNV	rs34820910	LOC646588	A	0.265	A	0.159
14:54465189-SNV	rs12890848		A	0.351	A	0.245
20:2606637-SNV	rs73572292	TMC2	C	0.114	C	0.008
10:36472891-SNV	rs55886049		C	0.210	C	0.103
10:21468925-SNV	rs7067884		A	0.126	A	0.019
8:75327897-SNV	rs6472847	GDAP1	A	0.447	A	0.340
4:144295025-SNV	rs56109066	GAB1	A	0.134	A	0.026
7:105800187-SNV	rs176551		C	0.407	C	0.299
12:53718446-SNV	rs17751196		G	0.307	G	0.199
11:62918915-SNV	rs7117048		G	0.484	G	0.373
2:240574274-SNV	rs183855386		A	0.119	A	0.008
2:149935828-SNV	rs4667359	LYPD6B	C	0.176	C	0.064
10:21410241-SNV	rs7909462	NEBL	C	0.478	C	0.366

7:105826102-SNV	rs10261981		G	0.113		0.000
5:112951922-SNV	rs2914197		G	0.377	G	0.263
6:150158194-SNV	rs4870049	LRP11	T	0.456	T	0.342
5:66862890-SNV	rs7710443		G	0.312	G	0.193
17:42713311-SNV	rs148242655		A	0.232	A	0.112
11:134769411-SNV	rs10894900		G	0.299	G	0.179
7:102168079-SNV	rs113088277		T	0.415	T	0.295
11:51585713-SNV	rs147532289		A	0.446	A	0.324
11:101993898-SNV	rs11225138	YAP1	C	0.243	C	0.117
17:77643452-SNV	rs4493094		G	0.415	G	0.286
10:21400606-SNV	rs12358463	NEBL	T	0.268	T	0.137
10:36469962-SNV	rs315633		T	0.478	T	0.304
14:54463354-SNV	rs17127137		A	0.141	A	0.293
5:20901312-SNV	rs10941866	LINC02241	G	0.334	G	0.485
5:83650273-SNV	rs7722323	EDIL3	A	0.352	G	0.493
12:42671923-SNV	rs12322512		G	0.200	G	0.340
5:46113314-SNV	rs56214607		A	0.241	A	0.377
10:36473044-SNV	rs899865		C	0.329	C	0.464
21:11132295-SNV	rs2770477		A	0.127	A	0.255
16:6716709-SNV	rs8054766	RBFOX1	A	0.278	A	0.405
2:241364552-SNV	rs7587308		A	0.368	A	0.490
5:35858721-SNV	rs1389832	IL7R	G	0.364	A	0.484

16:3984034-SNV	rs62036894		G	0.222	G	0.342
21:11124199-SNV	rs12626867		G	0.188	G	0.306
7:57085315-SNV	rs374748208		A	0.255	A	0.370
2:14878577-SNV	rs2571625		T	0.324	T	0.439
7:57084299-SNV	rs62463108		G	0.380	G	0.495
19:24578299-SNV	rs147074605		T	0.322	T	0.434
18:21250699-SNV	rs12960692		T	0.329	T	0.440
2:240577962-SNV	rs2083113		T	0.371	T	0.483
7:57019228-SNV	rs62463997		C	0.180	C	0.290
17:22013372-SNV	rs12943568		A	0.351	A	0.460
4:32439916-SNV	rs9683954		A	0.358	A	0.467
2:101213932-SNV	rs78374412		T	0.288	T	0.396
1:162225278-SNV	rs10918998	NOS1AP	A	0.207	A	0.314
9:98209594-SNV	rs357564	PTCH1	A	0.342	A	0.447
8:1140892-SNV	rs13277918	DLGAP2	T	0.323	T	0.428
11:102083695-SNV	rs10895276	YAP1	T	0.223	T	0.328
12:90704974-SNV	rs10858971		T	0.352	T	0.454
20:41762995-SNV	rs6130299	PTPRT	A	0.311	A	0.413
9:20859847-SNV	rs10757152	FOCAD	T	0.396	T	0.495
7:137113962-SNV	rs10272842	DGKI	C	0.067	C	0.166
6:90989125-SNV	rs7754251	BACH2	G	0.340	G	0.439
2:207107801-SNV	rs6744145	GPR1-AS	C	0.253	C	0.352

17:77471383-SNV	rs34470475	RBFOX3	T	0.203	T	0.302
9:97672169-SNV	rs16911824	AOPEP	C	0.363	C	0.461
12:42538847-SNV	rs10880254	GXYLT1	C	0.263	C	0.361
11:59955357-SNV	rs1834557		A	0.369	A	0.467
14:46666423-SNV	rs72684686	LINC00871	G	0.251	G	0.349
2:81450976-SNV	rs1379389		G	0.386	G	0.484
12:133020683-SNV	rs12314373		A	0.254	A	0.352
21:9543680-SNV	rs371609647		C	0.331	C	0.429
21:10820880-SNV	rs28848777		C	0.363	C	0.460
22:24109462-SNV	rs131443	CHCHD10	C	0.336	C	0.434
9:205252-SNV	rs12554654		C	0.189	C	0.286
4:70466280-SNV	rs10028954	UGT2A1,UGT2A2	T	0.355	T	0.452
7:57246259-SNV	rs4870688	GUSBP10	T	0.268	T	0.365
1:40560458-SNV	rs6671113	PPT1	G	0.189	G	0.286
14:46706676-SNV	rs858912	LINC00871	A	0.262	A	0.358
5:56144016-SNV	rs62358076	MAP3K1	C	0.159	C	0.255
11:51583409-SNV	rs4881779		C	0.386	C	0.481
1:217869432-SNV	rs141377903	SPATA17	A	0.230	A	0.324
13:80759894-SNV	rs144643045		T	0.182	T	0.276
6:62817919-SNV	rs9454321	KHDRBS2	T	0.376	T	0.470
11:99204233-SNV	rs11600851	CNTN5	T	0.268	T	0.362
1:14460133-SNV	rs10803458		C	0.358	C	0.452

5:80263865-SNV	rs6874731	RASGRF2	G	0.378	G	0.471
1:42285039-SNV	rs1837562	HIVEP3	A	0.247	A	0.340
6:3967385-SNV	rs9392538		A	0.369	A	0.462
8:146009176-SNV	rs6993407	ZNF34	C	0.275	C	0.367
4:136243221-SNV	rs11727438		T	0.179	T	0.271
21:23747453-SNV	rs8128244		G	0.286	G	0.377
6:146384950-SNV	rs9322045	GRM1	C	0.400	G	0.491
4:88482482-SNV	rs62315972		G	0.407	A	0.498
7:56275072-SNV	rs553090179		C	0.240	C	0.330
12:42801652-SNV	rs1669899	PPHLN1	C	0.218	C	0.307
6:88667123-SNV	rs663370		C	0.326	C	0.416
4:78951728-SNV	rs11936798		A	0.367	A	0.457
14:46705074-SNV	rs11850151	LINC00871	T	0.124	T	0.214
7:57122473-SNV	rs9690157		A	0.286	A	0.375
8:12930854-SNV	rs496443		G	0.259	G	0.349
1:163282358-SNV	rs3010370	RGS5	A	0.224	A	0.313
3:149114762-SNV	rs34119842		C	0.296	C	0.384
11:17942541-SNV	rs757410	SERGEF	C	0.404	A	0.493
6:150161242-SNV	rs11968879	LRP11	A	0.084	A	0.172
4:125804197-SNV	rs12374334		G	0.405	A	0.493
2:230500992-SNV	rs58887285	DNER	C	0.236	C	0.324
8:75268937-SNV	rs13281425	GDAP1	G	0.134	G	0.221

1:16509029-SNV	rs1007887		T	0.404	C	0.491
11:37154797-SNV	rs2704920		T	0.182	T	0.269
17:79611410-SNV	rs62075724	TSPAN10	C	0.312	C	0.399
15:66445779-SNV	rs7176258	MEGF11	C	0.191	C	0.278
11:119651489-SNV	rs377656976		C	0.249	C	0.335
13:95411450-SNV	rs9516470		G	0.255	G	0.341
5:2821733-SNV	rs13182538		A	0.403	G	0.489
2:121580431-SNV	rs7561607	GLI2	C	0.337	C	0.423
20:32265747-SNV	rs2071055	E2F1	A	0.192	A	0.278
16:76895693-SNV	rs9673839		G	0.322	G	0.408
8:3806794-SNV	rs2740868	CSMD1	T	0.185	T	0.270
8:62891786-SNV	rs344303		C	0.243	C	0.328
1:32438234-SNV	rs680328		G	0.275	G	0.360
11:94064679-SNV	rs560774		C	0.212	C	0.297
4:8515739-SNV	rs56075972			0	T	0.085
19:46217026-SNV	rs3760842	FBXO46	A	0.225	A	0.310
10:80709254-SNV	rs6480913	ZMIZ1-AS1	G	0.321	G	0.405
7:57076705-SNV	rs200985245		T	0.095	T	0.179
6:32986573-SNV	rs6457701		G	0.410	G	0.494
6:109727026-SNV	rs57919077	PPIL6	G	0.026	G	0.110
13:92130295-SNV	rs9589247	GPC5	C	0.127	C	0.211
4:45743536-SNV	rs62306269		G	0.257	G	0.341

3:37453112-SNV	rs13066950	C3orf35	T	0.302	T	0.386
1:208762066-SNV	rs12728670		A	0.302	A	0.386
4:103014305-SNV	rs72688520		A	0.392	A	0.476
19:20789047-SNV	rs7255372		A	0.170	A	0.254
6:8494201-SNV	rs9505460	LOC100506207	A	0.385	A	0.468
6:32490925-SNV	rs116670844	HLA-DRB5	T	0.176	T	0.259
7:95769863-SNV	rs12704835	SLC25A13	T	0.179	T	0.262
8:140972770-SNV	rs7817647	TRAPPC9	C	0.327	C	0.411
13:70635545-SNV	rs4883854	KLHL1	T	0.080	T	0.163
6:64542625-SNV	rs4710443	EYS	T	0.230	T	0.312
2:236916259-SNV	rs66880965	AGAP1	T	0.320	T	0.402
4:154254516-SNV	rs72729647	TRIM2	A	0.280	A	0.363
10:10886848-SNV	rs10795827	CELF2	G	0.331	G	0.413
18:74906179-SNV	rs11151008		C	0.302	C	0.384
1:49060263-SNV	rs319964	AGBL4	A	0.316	A	0.398
7:77597913-SNV	rs6977199		C	0.192	C	0.274
20:2663309-SNV	rs4813603		A	0.111	A	0.193
21:43446474-SNV	rs75967781		A	0.188	A	0.269
3:189481894-SNV	rs4075773	TP63	A	0.225	A	0.307
8:125408304-SNV	rs62530048		G	0.114	G	0.196
7:101671793-SNV	rs12671456	CUX1	T	0.197	T	0.278
9:1735425-SNV	rs514282		T	0.211	T	0.292

7:101173010-SNV	rs13238548	COL26A1	A	0.243	A	0.324
18:53965799-SNV	rs2058494		C	0.074	C	0.155
8:16293973-SNV	rs79168220		G	0.203	G	0.284
9:83795146-SNV	rs7020372		A	0.344	A	0.425
10:80760771-SNV	rs34706430	ZMIZ1-AS1	G	0.077	G	0.158
9:8935185-SNV	rs66867787	PTPRD	A	0.360	A	0.441
9:7537343-SNV	rs10815632		G	0.236	G	0.317
3:47547037-SNV	rs117768043	ELP6	C	0.041	C	0.121
13:53321391-SNV	rs1812735		C	0.287	C	0.367
3:59410065-SNV	rs4073471		C	0.414	C	0.495
3:185905876-SNV	rs13087397	DGKG	A	0.407	A	0.487
2:43115502-SNV	rs35927294		A	0.209	A	0.289
18:8657546-SNV	rs365631		T	0.349	T	0.429
13:53326437-SNV	rs12146944		A	0.088	A	0.168
16:80007084-SNV	rs565361605		A	0.326	A	0.406
8:125418855-SNV	rs4440618		G	0.414	A	0.494
3:73021369-SNV	rs62249930	GXYLT2	T	0.341	T	0.420
1:4638061-SNV	rs12724503	LINC01646	A	0.386	A	0.465
10:106488882-SNV	rs1387831	SORCS3	C	0.265	C	0.344
17:71544306-SNV	rs1078439	SDK2	C	0.229	C	0.307
2:192592113-SNV	rs4334444		C	0.316	C	0.395
17:42719228-SNV	rs7502334		A	0.192	A	0.271

4:98168696-SNV	rs35640761		A	0.254	A	0.332
10:106499611-SNV	rs1565415	SORCS3	C	0.415	T	0.494
2:61897554-SNV	rs11682878		A	0.308	A	0.386
10:30098732-SNV	rs10826704		T	0.213	T	0.291
2:231168900-SNV	rs6708227	SP140	G	0.241	G	0.318
7:13896259-SNV	rs740263		T	0.304	T	0.382
8:12307315-SNV	rs2719579	LOC100506990	A	0.336	A	0.414
20:41774870-SNV	rs6030669	PTPRT	T	0.405	T	0.483
11:102003827-SNV	rs61918362	YAP1	A	0.163	A	0.240
19:20847824-SNV	rs7259377		T	0.266	T	0.343
16:13012979-SNV	rs11646217	SHISA9	A	0.224	A	0.301
10:100188106-SNV	rs10883094	HPS1	A	0.352	A	0.429
15:26616418-SNV	rs67624821		G	0.175	G	0.252
11:62922731-SNV	rs72922333		A	0.109	A	0.186
9:220070-SNV	rs663013	DOCK8	T	0.236	T	0.313
5:120491841-SNV	rs35049166		T	0.268	T	0.345
10:72576882-SNV	rs41315008	SGPL1	C	0.242	C	0.318
2:236549409-SNV	rs10194516	AGAP1	G	0.385	G	0.461
13:81229280-SNV	rs1176323		G	0.390	G	0.467
10:11166027-SNV	rs1750732	CELF2	G	0.359	G	0.436
4:106022992-SNV	rs4141270		T	0.137	T	0.214
7:145363652-SNV	rs12703731		T	0.415	T	0.491

5:169573192-SNV	rs4867582		G	0.184	G	0.259
17:66773961-SNV	rs7217811		A	0.345	A	0.421
6:62843610-SNV	rs209006	KHDRBS2	A	0.176	A	0.252
8:146068846-SNV	rs1735172	ZNF7	A	0.234	A	0.310
2:121579604-SNV	rs4848638	GLI2	A	0.224	A	0.300
8:78230550-SNV	rs72665605		G	0.190	G	0.266
7:20082997-SNV	rs112211835	LOC101927668	C	0.092	C	0.168
12:46480442-SNV	rs77334042		G	0.013	G	0.089
2:237396935-SNV	rs6751722	IQCA1	A	0.243	A	0.318
18:45717664-SNV	rs1893835	ZBTB7C	G	0.408	G	0.482
7:20122204-SNV	rs10232067	LOC101927668	C	0.252	C	0.326
5:56332053-SNV	rs1526888		G	0.167	G	0.241
13:113705260-SNV	rs66526251	MCF2L	A	0.424	A	0.498
11:21528562-SNV	rs11026115	NELL1	T	0.197	T	0.271
12:120859188-SNV	rs2516079		G	0.382	G	0.457
11:95780451-SNV	rs1939483	MAML2	A	0.200	A	0.274
19:41711251-SNV	rs79871235	CYP2S1	T	0.179	T	0.253
2:10069327-SNV	rs56241824	TAF1B	A	0.243	A	0.317
7:130633423-SNV	rs6953084	LINC-PINT	G	0.418	G	0.491
9:20869345-SNV	rs79660000	FOCAD	A	0.227	A	0.301
9:83790397-SNV	rs2777678		C	0.246	C	0.320
12:117633362-SNV	rs11068408		G	0.109	G	0.182

6:134329997-SNV	rs2811675	SLC2A12	C	0.411	C	0.484
4:18756789-SNV	rs1464189		A	0.398	A	0.471
12:55272683-SNV	rs10876641		A	0.416	C	0.490
13:92112473-SNV	rs35345458	GPC5	T	0.247	T	0.321
6:78293135-SNV	rs75815955		G	0.176	G	0.249
5:61974663-SNV	rs1306283		T	0.155	T	0.228
10:21438409-SNV	rs12261588	NEBL	A	0.142	A	0.215
8:134221502-SNV	rs4330674	CCN4	T	0.343	T	0.416
1:111016261-SNV	rs12142066		A	0.420	A	0.493
7:34281928-SNV	rs4720156		G	0.151	G	0.224
10:59353746-SNV	rs10826090		A	0.241	A	0.314
1:113645629-SNV	rs200523711	LRIG2	T	0.299	T	0.372
8:5828855-SNV	rs2816434		C	0.148	C	0.221
2:34643475-SNV	rs4670362		G	0.349	G	0.422
2:43099956-SNV	rs4299380		T	0.225	T	0.298
15:39559675-SNV	rs12907606		T	0.419	C	0.491
2:119939028-SNV	rs4849765		T	0.364	T	0.436
1:116567296-SNV	rs1775711	SLC22A15	T	0.279	T	0.352
3:42185562-SNV	rs9311300	TRAK1	G	0.354	G	0.426
4:77843690-SNV	rs12648505		T	0.113	T	0.186
14:24217467-SNV	rs11850283		C	0.066	C	0.138
7:95808541-SNV	rs11761193	SLC25A13	A	0.227	A	0.300

21:14707364-SNV	rs557899449;rs2982814		T	0.085	T	0.157
2:139189898-SNV	rs144351178		T	0.032	T	0.104
1:96946785-SNV	rs12097450		C	0.193	C	0.266
13:37746363-SNV	rs4054540		T	0.127	T	0.200
7:114119430-SNV	rs2189010	FOXP2	G	0.244	G	0.316
4:67938655-SNV	rs6830546		T	0.236	T	0.308
1:151518486-SNV	rs572303788	TUFT1	C	0.160	C	0.232
4:12188297-SNV	rs13114953		C	0.256	C	0.328
12:33745118-SNV	rs74072858			0	A	0.071
21:16168714-SNV	rs9975420		C	0.426	A	0.498
8:8043191-SNV	rs793758	FAM85B	T	0.260	T	0.332
2:62708186-SNV	rs6545944		G	0.253	G	0.324
7:40612227-SNV	rs6978599	SUGCT	G	0.240	G	0.311
7:153015547-SNV	rs10246446		A	0.343	A	0.414
2:31428904-SNV	rs9752146	CAPN14	A	0.256	A	0.327
6:70266902-SNV	rs9346308		A	0.391	A	0.462
21:14596767-SNV	rs2801236		A	0.085	A	0.155
19:51328736-SNV	rs3212810	KLK15	T	0.201	T	0.272
3:45459287-SNV	rs2286907	LARS2	G	0.426	T	0.497
1:10484416-SNV	rs663364		G	0.340	G	0.410
7:24406124-SNV	rs4722350		T	0.416	T	0.487

6:5378009-SNV	rs12525845	FARS2	G	0.345	G	0.415
7:101095919-SNV	rs6943584	COL26A1	C	0.189	C	0.259
10:116663513-SNV	rs11196977		A	0.068	A	0.138
2:107016431-SNV	rs142537870		T	0.235	T	0.305
7:3588188-SNV	rs6966652	SDK1	A	0.365	A	0.435
1:167143281-SNV	rs12029632		T	0.286	T	0.356
13:93821033-SNV	rs12867908		A	0.415	A	0.485
15:29042581-SNV	rs2880910	PDCD6IPP2	A	0.253	A	0.322
12:3214998-SNV	rs2878394	TSPAN9	T	0.401	T	0.470
22:27382132-SNV	rs2157466		G	0.290	G	0.359
3:1512068-SNV	rs13095097		A	0.095	A	0.164
1:72508357-SNV	rs1486088	NEGR1	A	0.198	A	0.267
14:104513043-SNV	rs12878767	TDRD9	T	0.327	T	0.397
18:49197333-SNV	rs9947314		A	0.301	A	0.370
17:67400254-SNV	rs817575		T	0.060	T	0.130
15:78797463-SNV	rs9672608		A	0.320	A	0.389
7:128153667-SNV	rs10241631		A	0.357	A	0.426
10:8120493-SNV	rs417777		G	0.201	G	0.270
1:199228114-SNV	rs6684788	LINC02789	T	0.381	T	0.450
2:36376478-SNV	rs74926123		C	0.030	C	0.099
6:107137833-SNV	rs2354551		T	0.260	T	0.329
11:95775422-SNV	rs7934969	MAML2	A	0.142	A	0.210

3:26559499-SNV	rs13063974		A	0.089	A	0.158
8:20281811-SNV	rs35490893		T	0.333	T	0.401
17:15607383-SNV	rs163375	ZNF286A	A	0.098	A	0.166
2:81591163-SNV	rs6547367		A	0.344	A	0.412
3:196379993-SNV	rs965140	NRROS	T	0.127	T	0.196
2:25140527-SNV	rs73920610	ADCY3	A	0.231	A	0.299
5:165048301-SNV	rs262749		C	0.249	C	0.318
8:81836812-SNV	rs895271		G	0.366	G	0.434
2:104984019-SNV	rs2376160		C	0.138	C	0.207
7:77526992-SNV	rs12537101	PHTF2	T	0.086	T	0.154
4:32252530-SNV	rs12503077		T	0.142	T	0.210
7:57128425-SNV	rs9719133		A	0.044	A	0.112
16:76845390-SNV	rs62049263		T	0.205	T	0.273
2:48889446-SNV	rs11125178	GTF2A1L,STON1-GTF2A1L	A	0.393	A	0.461
13:92778177-SNV	rs7492094	GPC5	G	0.235	G	0.303
1:86637665-SNV	rs12567202		A	0.124	A	0.192
6:3405558-SNV	rs11242856	SLC22A23	A	0.173	A	0.240
15:97666441-SNV	rs1398964		C	0.424	C	0.491
17:41435192-SNV	rs1842147		T	0.411	C	0.478
3:75974251-SNV	rs7611923	ROBO2	C	0.131	C	0.198
2:34012974-SNV	rs56310158	LINC01317	G	0.110	G	0.177

16:65276353-SNV	rs12920405		A	0.097	A	0.164
1:95547679-SNV	rs9437687		A	0.427	A	0.495
21:20419551-SNV	rs2825318		G	0.200	G	0.267
7:96399197-SNV	rs17167790		T	0.102	T	0.169
3:65078405-SNV	rs1842800		C	0.232	C	0.299
12:111807499-SNV	rs933399	PHETA1	G	0.433	G	0.500
13:92072346-SNV	rs12561118	GPC5	C	0.140	C	0.207
6:40253685-SNV	rs79321845		A	0.140	A	0.207
2:18275620-SNV	rs13395500		C	0.327	C	0.394
1:1378173-SNV	rs711180	VWA1	A	0.275	A	0.342
19:39615797-SNV	rs774906	PAK4	T	0.384	T	0.450
18:3724602-SNV	rs13381043	DLGAP1	A	0.395	A	0.461
3:71673581-SNV	rs830631		G	0.426	G	0.493
10:10874236-SNV	rs28677866	CELF2	T	0.127	T	0.194
21:11028552-SNV	rs633366	BAGE2	T	0.022	T	0.089
5:65789729-SNV	rs4379144		G	0.281	G	0.348
17:25691296-SNV	rs11080143		C	0.279	C	0.345
16:17746427-SNV	rs34342618		C	0.174	C	0.240
9:89246924-SNV	rs7851559		T	0.327	T	0.394
2:120399882-SNV	rs34187712	CFAP221	C	0.185	C	0.251
13:65391510-SNV	rs57465948		A	0.162	A	0.227
2:37999909-SNV	rs9973704		G	0.278	G	0.344

8:23194928-SNV	rs4556093	LOC100507156,LOXL2	A	0.336	A	0.402
10:51735345-SNV	rs67491774		G	0.225	G	0.291
12:10029521-SNV	rs11609546		T	0.263	T	0.328
11:51491426-SNV	rs4881761		A	0.178	A	0.244
6:33553677-SNV	rs395671	GGNBP1	C	0.424	G	0.490
12:33749794-SNV	rs6488173		C	0.110	C	0.175
11:68889182-SNV	rs11228506		A	0.343	A	0.408
6:151122573-SNV	rs9383849	PLEKHG1	T	0.322	T	0.387
3:172974531-SNV	rs10936746		A	0.140	A	0.205
4:162906128-SNV	rs59190885	FSTL5	C	0.185	C	0.250
8:63494447-SNV	rs74209385	NKAIN3	C	0.333	C	0.398
1:109672711-SNV	rs6694052	ELAPOR1	C	0.177	C	0.242
9:82174027-SNV	rs10746592		C	0.299	C	0.364
1:59572439-SNV	rs2989867		A	0.357	A	0.422
21:41600188-SNV	rs445018	DSCAM	C	0.429	T	0.494
1:190948513-SNV	rs6663211		C	0.263	C	0.328
9:136534694-SNV	rs9409878	SARDH	C	0.321	C	0.386
18:11374161-SNV	rs34256218		A	0.210	A	0.275
1:111849349-SNV	rs12403961	CHIA	G	0.144	G	0.209
12:92827016-SNV	rs12578375	CLLU1-AS1	T	0.118	T	0.182
14:59943177-SNV	rs1270493	L3HYPDH	A	0.020	A	0.085
16:31783668-SNV	rs2078924		A	0.258	A	0.323

11:82584464-SNV	rs7121090	PRCP	G	0.063	G	0.127
9:97667934-SNV	rs12684823	AOPEP	A	0.153	A	0.217
7:142435691-SNV	rs7810050		A	0.386	A	0.450
13:113328525-SNV	rs7337905	ATP11AUN	G	0.262	G	0.326
11:82573788-SNV	rs3793981	PRCP	C	0.151	C	0.215
12:69367332-SNV	rs6581841		T	0.338	T	0.403
6:36100732-SNV	rs2071864	MAPK13	C	0.193	C	0.258
1:147364430-SNV	rs2352835		A	0.424	G	0.488
16:66183496-SNV	rs74673283		T	0.012	T	0.076
21:18817530-SNV	rs9305816	LINC01549	A	0.264	A	0.328
13:69791693-SNV	rs9634953		T	0.074	T	0.137
11:44521492-SNV	rs4755258		C	0.418	C	0.481
7:16977090-SNV	rs860196		T	0.299	T	0.363
10:127883810-SNV	rs11244878	ADAM12	C	0.154	C	0.217
9:83352214-SNV	rs10780461		A	0.355	A	0.418
18:21902331-SNV	rs112021519	OSBPL1A	A	0.186	A	0.249
10:103702763-SNV	rs11191156	ARMH3	G	0.244	G	0.307
1:42253680-SNV	rs2813898	HIVEP3	C	0.075	C	0.138
9:90320714-SNV	rs36220119	DAPK1	G	0.035	G	0.099
4:40700135-SNV	rs11722714		A	0.300	A	0.363
14:33371543-SNV	rs7142315		T	0.311	T	0.374
3:60611316-SNV	rs2156975	FHIT	T	0.031	T	0.094

8:122444919-SNV	rs1027035		G	0.388	G	0.451
2:23227574-SNV	rs4665190		G	0.160	G	0.224
9:83404085-SNV	rs12346044		A	0.362	A	0.425
20:18957393-SNV	rs11905621		C	0.243	C	0.306
4:159219687-SNV	rs2346378		C	0.365	C	0.428
9:26630105-SNV	rs114445294		A	0.079	A	0.142
11:24602653-SNV	rs7935768	LUZP2	A	0.280	A	0.343
21:9491645-SNV	rs371747243		T	0.124	T	0.187
4:57602972-SNV	rs146924265		G	0.323	G	0.386
11:4265161-SNV	rs437350		C	0.231	C	0.293
3:178527651-SNV	rs4493412	KCNMB2,KCNMB2-AS1	T	0.432	T	0.495
7:57047572-SNV	rs78664580		G	0.035	G	0.098
8:99210175-SNV	rs13439613	NIPAL2	T	0.197	T	0.259
7:155886219-SNV	rs113403524		C	0.004	C	0.067
13:79980609-SNV	rs2296286	RBM26-AS1	A	0.076	A	0.138
5:19024578-SNV	rs7705600		A	0.134	A	0.196
8:15989207-SNV	rs9657212	MSR1	A	0.166	A	0.228
3:190520710-SNV	rs7649452		C	0.425	T	0.488
1:95394831-SNV	rs1265168	CNN3-DT	G	0.148	G	0.210
4:109848103-SNV	rs17596971	COL25A1	G	0.421	G	0.483
3:132720447-SNV	rs199850233		T	0.310	T	0.372
5:104529305-SNV	rs112415042		C	0.127	C	0.189

1:224847209-SNV	rs199773364	CNIH3	A	0.107	A	0.168
2:40573214-SNV	rs576221341	SLC8A1	A	0.266	A	0.328
4:161410867-SNV	rs1595665		T	0.110	T	0.172
14:59583319-SNV	rs4901896		G	0.226	G	0.288
5:22685567-SNV	rs2126963	CDH12	C	0.102	C	0.164
13:26700180-SNV	rs9507671		G	0.245	G	0.307
10:122580645-SNV	rs12219908	WDR11-AS1	T	0.147	T	0.209
14:105784927-SNV	rs10134192	PACS2	T	0.166	T	0.227
11:86632288-SNV	rs7119097		T	0.346	T	0.408
20:47500404-SNV	rs6125486		T	0.338	T	0.400
5:122958354-SNV	rs77704754		A	0.299	A	0.360
6:131128662-SNV	rs34525579		C	0.199	C	0.260
21:9524068-SNV	rs376837409		A	0.075	A	0.136
2:15405663-SNV	rs6738994	NBAS	G	0.385	G	0.446
21:45899244-SNV	rs2096861		T	0.377	T	0.438
6:134465900-SNV	rs9321428		C	0.142	C	0.203
1:214519615-SNV	rs7528860		A	0.433	A	0.494
11:119727452-SNV	rs6589781		A	0.393	A	0.454
9:120407672-SNV	rs10818057		G	0.296	G	0.356
5:10185745-SNV	rs7724835		A	0.341	A	0.401
4:94384868-SNV	rs7669747	GRID2	C	0.344	C	0.405
2:181961158-SNV	rs35706726		A	0.148	A	0.209

8:143226798-SNV	rs7461193		T	0.291	T	0.352
2:105591795-SNV	rs11124011	MRPS9-AS2	C	0.167	C	0.227
2:109316789-SNV	rs7575335		A	0.387	A	0.447
3:98290543-SNV	rs56028800		G	0.353	G	0.413
3:2876612-SNV	rs4685568	CNTN4	C	0.260	C	0.321
1:97783868-SNV	rs7415016	DPYD,DPYD-AS1	T	0.187	T	0.247
15:74462429-SNV	rs199736118		T	0.316	T	0.377
5:16085065-SNV	rs16868089	MARCHF11	G	0.031	G	0.091
19:54540218-SNV	rs2448286		C	0.354	C	0.414
4:88346510-SNV	rs6816224	NUDT9	A	0.318	A	0.377
18:54889808-SNV	rs17063255		C	0.318	C	0.377
6:78746361-SNV	rs142599727		A	0.082	A	0.142
3:183407065-SNV	rs9816948		T	0.154	T	0.214
16:65276310-SNV	rs111255807		A	0.003	A	0.063
10:94066971-SNV	rs1928352	MARCHF5	C	0.133	C	0.193
21:41328086-SNV	rs996127		A	0.281	A	0.341
19:51333674-SNV	rs7246740	KLK15	G	0.054	G	0.113
14:85114514-SNV	rs72694481		T	0.125	T	0.185
9:138324992-SNV	rs7860057		C	0.385	C	0.444
2:38008668-SNV	rs62135780		C	0.215	C	0.275
3:194318472-SNV	rs2794667	TMEM44	A	0.435	A	0.495
3:71421314-SNV	rs13314303	FOXP1	T	0.123	T	0.182

12:85591217-SNV	rs370833022	LRRIQ1	A	0.012	A	0.071
15:27464356-SNV	rs12911451	GABRG3	A	0.433	A	0.492
3:95627438-SNV	rs35469482		T	0.198	T	0.257
3:196554952-SNV	rs6789365	PAK2	A	0.185	A	0.244
10:32046865-SNV	rs2797135		G	0.243	G	0.302
13:87758181-SNV	rs1522017		G	0.373	G	0.432
7:57010310-SNV	rs142206645		G	0.074	G	0.133
20:23027621-SNV	rs1042580	THBD	C	0.378	C	0.437
5:56188803-SNV	rs7724841	MAP3K1	T	0.085	T	0.144
11:34314986-SNV	rs2093325	ABTB2	G	0.273	G	0.332
20:129635-SNV	rs12480202		C	0.273	C	0.332
20:1342865-SNV	rs912114;rs558332405	FKBP1A-SDCBP2,SDCBP2-AS1	C	0.434	C	0.493
2:78313224-SNV	rs34268871	LOC101927967	C	0.180	C	0.239
7:151457046-SNV	rs62478212	PRKAG2	T	0.146	T	0.205
9:18218500-SNV	rs4961628		C	0.062	C	0.120
2:215072878-SNV	rs62196174	SPAG16	T	0.178	T	0.237
18:69346061-SNV	rs7232078		T	0.295	T	0.353
16:9154424-SNV	rs7198738		G	0.131	G	0.189
12:109635277-SNV	rs2268390	ACACB	T	0.174	T	0.232
6:170967926-SNV	rs371723056		T	0.147	T	0.206
14:97520929-SNV	rs17094887		T	0.049	T	0.108

1:86616819-SNV	rs571326026	COL24A1	C	0.015	C	0.074
4:65420044-SNV	rs17085060		T	0.034	T	0.092
20:56307546-SNV	rs1556179		G	0.254	G	0.312
11:21652727-SNV	rs990339		A	0.214	A	0.273
4:105845011-SNV	rs9637619		A	0.167	A	0.225
3:156009614-SNV	rs2290165	KCNAB1	G	0.432	G	0.490
5:168175040-SNV	rs4502845	SLIT3	G	0.104	G	0.162
1:156556321-SNV	rs10796961	TTC24	G	0.091	G	0.149
8:46981563-SNV	rs4461909		T	0.078	T	0.136
6:133452425-SNV	rs3904628		G	0.224	G	0.282
14:56501133-SNV	rs12432868		T	0.254	T	0.311
9:4329354-SNV	rs10814941		A	0.098	A	0.155
14:47644144-SNV	rs59466831	MDGA2	C	0.227	C	0.285
8:20359790-SNV	rs2046072		A	0.188	A	0.245
22:47506133-SNV	rs5767504	TBC1D22A	C	0.265	C	0.322
15:60287773-SNV	rs4485289		C	0.270	C	0.328
10:80771478-SNV	rs56812308	ZMIZ1-AS1	A	0.127	A	0.185
7:571464-SNV	rs28710432		C	0.107	C	0.164
10:76101042-SNV	rs75140991	ADK	A	0.009	A	0.066
21:33786483-SNV	rs8134043	EVA1C	C	0.197	C	0.254
7:147094863-SNV	rs7778767	CNTNAP2	A	0.020	A	0.077
11:120091272-SNV	rs12786658	OAF	A	0.213	A	0.270

1:217760416-SNV	rs10779299	GPATCH2	G	0.431	G	0.488
16:34628784-SNV	rs1212862		T	0.105	T	0.162
7:57972858-SNV	rs12672878		T	0.053	T	0.109
4:154257511-SNV	rs73854659	TRIM2	A	0.085	A	0.141
7:17650401-SNV	rs115083514			0	C	0.057
11:2300181-SNV	rs10741561		A	0.429	G	0.485
7:156016756-SNV	rs1922087		C	0.342	C	0.398
2:61883923-SNV	rs6738285		T	0.400	T	0.457
11:134945710-SNV	rs11823417		G	0.107	G	0.163
16:66187557-SNV	rs59030397		T	0.009	T	0.065
1:243046821-SNV	rs79709831		T	0.086	T	0.142
9:74270956-SNV	rs77886456		T	0.073	T	0.129
13:90634051-SNV	rs7335472		C	0.240	C	0.296
4:105067193-SNV	rs11509416		C	0.070	C	0.127
14:85859884-SNV	rs7141524	LINC00911	C	0.076	C	0.132
4:182025964-SNV	rs28407777	LINC00290	C	0.068	C	0.124
21:27663757-SNV	rs528196487		T	0.074	T	0.130
1:88472025-SNV	rs6672529		G	0.145	G	0.201
20:712185-SNV	rs6054380		T	0.132	T	0.188
2:102069748-SNV	rs6744377	RFX8	T	0.218	T	0.273
16:66167073-SNV	rs28493960		A	0.062	A	0.117
19:19260808-SNV	rs28672836	BORCS8-MEF2B,MEF2B	T	0.170	T	0.226

19:11158047-SNV	rs10420325	SMARCA4	A	0.229	A	0.284
17:7645326-SNV	rs12938680	DNAH2	G	0.367	G	0.422
7:20106806-SNV	rs17142371	LOC101927668	C	0.224	C	0.280
17:7659832-SNV	rs62062620	DNAH2	A	0.211	A	0.266
6:6720009-SNV	rs201049		A	0.211	A	0.266
21:28244976-SNV	rs113533189		T	0.042	T	0.097
15:63047778-SNV	rs8033767	TLN2	C	0.288	C	0.343
5:16734775-SNV	rs74934137	MYO10	A	0.119	A	0.174
9:27418638-SNV	rs10812589	MOB3B	G	0.436	A	0.491
2:82344278-SNV	rs7571388		C	0.436	T	0.491
1:148017424-SNV	rs200095021		C	0.116	C	0.172
21:43459172-SNV	rs220245		T	0.167	T	0.222
9:130480322-SNV	rs11792868	TTC16	C	0.075	C	0.130
20:55599379-SNV	rs1033677		C	0.133	C	0.188
6:96008211-SNV	rs34463787	MANEA-DT	T	0.236	T	0.291
17:7220697-SNV	rs62059198	NEURL4	T	0.210	T	0.265
8:78995713-SNV	rs10957882		C	0.311	C	0.366
8:136687535-SNV	rs56099413		T	0.084	T	0.138
6:88676604-SNV	rs75985024		A	0.160	A	0.215
15:25404297-SNV	rs12899489	SNHG14	C	0.076	C	0.130
19:53843284-SNV	rs140874614	ZNF845	T	0.055	T	0.109
11:38914113-SNV	rs1155099		G	0.262	G	0.316

11:63157889-SNV	rs113967277	SLC22A9	G	0.026	G	0.081
10:45897520-SNV	rs11239513	ALOX5		0	T	0.054
9:77886314-SNV	rs12235300		G	0.201	G	0.255
17:16813471-SNV	rs4578698		G	0.408	G	0.462
11:122833646-SNV	rs10892929	JHY	C	0.037	C	0.092
7:57125837-SNV	rs11769751		C	0.043	C	0.097
17:8574552-SNV	rs7502586		C	0.405	C	0.460
1:47643047-SNV	rs12135406		A	0.118	A	0.172
2:181283447-SNV	rs62180922		C	0.149	C	0.203
16:56681543-SNV	rs75135683		T	0.052	T	0.106
22:47490282-SNV	rs2337229	TBC1D22A	G	0.311	G	0.365
6:30297505-SNV	rs1264581	TRIM39,TRIM39-RPP21	T	0.097	T	0.151
4:96786478-SNV	rs72671138		A	0.142	A	0.196
1:185428891-SNV	rs7544994		G	0.132	G	0.186
18:75268493-SNV	rs7228382		A	0.111	A	0.165
11:117062158-SNV	rs139685157	SIDT2	A	0.071	A	0.125
4:144296249-SNV	rs3792654	GAB1	T	0.193	T	0.247
21:15452911-SNV	rs4453762		T	0.109	T	0.162
3:125226199-SNV	rs115728900	SNX4	A	0.037	A	0.091
1:41862914-SNV	rs560630161		A	0.096	A	0.149
3:145663771-SNV	rs1996112		G	0.368	G	0.422
20:14100766-SNV	rs6110171	MACROD2	G	0.225	G	0.279

1:43759733-SNV	rs1981080		T	0.440	T	0.493
17:54265906-SNV	rs59177695	ANKFN1	G	0.173	G	0.226
1:109384512-SNV	rs66518242	AKNAD1	A	0.062	A	0.115
11:85433442-SNV	rs142563341	SYTL2	C	0.041	C	0.094
16:84048540-SNV	rs6563993	SLC38A8	G	0.073	G	0.126
3:125322412-SNV	rs59890508		C	0.073	C	0.126
15:95097543-SNV	rs11635156		C	0.104	C	0.158
3:32163144-SNV	rs35642355	GPD1L	A	0.319	A	0.372
4:133154882-SNV	rs67935670		A	0.065	A	0.118
13:94145827-SNV	rs7399687	GPC6	T	0.298	T	0.351
12:9270408-SNV	ss1388095087		G	0.298	G	0.351
4:167126339-SNV	rs28463090		A	0.084	A	0.137
10:83606306-SNV	rs11191529		A	0.264	A	0.317
3:155033234-SNV	rs73007812		A	0.055	A	0.108
20:15069096-SNV	rs56964792	MACROD2	A	0.145	A	0.198
19:53284186-SNV	rs8101566	ZNF600	G	0.182	G	0.235
14:33564876-SNV	rs12432639	NPAS3	A	0.254	A	0.307
5:17162428-SNV	rs186739936	BASP1-AS1		0	T	0.053
5:49867497-SNV	rs80256493			0	G	0.053
12:6343123-SNV	rs11064098	CD9	T	0.058	T	0.111
11:55030984-SNV	rs115233128	TRIM48	G	0.058	G	0.111
2:87917635-SNV	rs112505234		T	0.231	T	0.283

2:81267220-SNV	rs35404608		A	0.067	A	0.120
14:33255611-SNV	rs4981161	AKAP6	G	0.242	G	0.294
13:71960706-SNV	rs148625842		T	0.110	T	0.162
4:159921928-SNV	rs71609024	C4orf45	G	0.055	G	0.107
8:102146644-SNV	rs28583389		G	0.188	G	0.240
3:104251956-SNV	rs1828948		T	0.109	T	0.161
14:98679169-SNV	rs1257906		A	0.154	A	0.206
22:20502771-SNV	rs1659630		T	0.114	T	0.166
15:58528746-SNV	rs7181221		A	0.136	A	0.188
3:162265108-SNV	rs1382198		C	0.123	C	0.175
11:126288762-SNV	rs4935966		T	0.200	T	0.252
20:9648401-SNV	rs6039542	PAK5	T	0.192	T	0.244
1:47405581-SNV	rs9332985	CYP4A11	T	0.113	T	0.165
4:105221940-SNV	rs115215212			0	G	0.051
15:24194457-SNV	rs1870544		G	0.045	G	0.096
7:129265800-SNV	rs11768624	NRF1	A	0.233	A	0.284
3:32272979-SNV	rs11710979		T	0.297	T	0.348
21:10513240-SNV	rs79407097		C	0.011	C	0.062
6:151786521-SNV	rs9383571	ARMT1	A	0.088	A	0.139
8:61625240-SNV	rs60155805	CHD7	A	0.178	A	0.229
11:55810147-SNV	rs2086561		T	0.059	T	0.110
6:94839309-SNV	rs683098		C	0.396	C	0.446

11:105136462-SNV	rs11606277		A	0.089	A	0.140
3:151186206-SNV	rs73869090		C	0.063	C	0.113
19:33250683-SNV	rs17755379	TDRD12	G	0.074	G	0.124
10:83090436-SNV	rs10883012		T	0.090	T	0.141
3:112900031-SNV	rs73229013		C	0.064	C	0.114
9:20599189-SNV	rs637029	MLLT3	C	0.193	C	0.244
20:22022091-SNV	rs56658024		T	0.024	T	0.075
2:135202455-SNV	rs535900	MGAT5	G	0.440	A	0.490
11:44918572-SNV	rs835864	TSPAN18	G	0.284	G	0.334
6:145194688-SNV	rs73007465		C	0.056	C	0.106
2:234123010-SNV	rs7580869		A	0.152	A	0.202
20:23626648-SNV	rs911123		C	0.007	C	0.057
1:226953246-SNV	rs835665		A	0.110	A	0.160
9:90316751-SNV	rs57634888	DAPK1	C	0.012	C	0.062
15:86122875-SNV	rs34434221	AKAP13	C	0.044	C	0.094
14:91627536-SNV	rs12432858	DGLUCY	T	0.444	T	0.494
2:222882489-SNV	rs6752645		T	0.066	T	0.116
3:130233515-SNV	rs2132827		A	0.410	A	0.460
1:90513479-SNV	rs12045708		T	0.071	T	0.121
12:96468929-SNV	rs34074403		T	0.273	T	0.322
12:24512352-SNV	rs11047393	SOX5	T	0.103	T	0.153
18:34010998-SNV	rs1708693	FHOD3	A	0.199	A	0.248

Supplementary Table 7: The SNPs and genes associated with lower and upper deciles in Europeans

			Lower deciles		Upper deciles	
Predictor	Identifier	Gene Names	Minor Allele	Minor Allele Frequency	Minor Allele	Minor Allele Frequency
15:69596677-SNV	rs12440717	PAQR5	A	0.069	A	0.019
17:41442419-SNV	rs117681499		T	0.101	T	0.051
8:128424633-SNV	rs76503658	CASC8	C	0.123	C	0.074
13:79801863-SNV	rs9318616		G	0.373	G	0.324
14:48297319-SNV	rs144165157		G	0.165	G	0.115
13:63640972-SNV	rs17319124		C	0.070	C	0.020
14:90011594-SNV	rs8009828	FOXN3	T	0.067	T	0.017
11:92261049-SNV	rs4753409	FAT3	T	0.105	T	0.055
21:29989055-SNV	rs8130464		T	0.128	T	0.078
16:30455458-SNV	rs1133238	SEPHS2	A	0.460	A	0.410
9:103988006-SNV	rs61188842	PLPPR1	T	0.153	T	0.103
5:144588235-SNV	rs7725692		A	0.138	A	0.088
2:123166454-SNV	rs62168353		G	0.302	G	0.252
21:18011330-SNV	rs8131576		C	0.094	C	0.043
12:30207397-SNV	rs28427143		G	0.129	G	0.078
14:39967531-SNV	rs17696574		A	0.293	A	0.243
7:51520397-SNV	rs1917292		T	0.081	T	0.031
2:7296659-SNV	rs16865979		A	0.243	A	0.193
6:68445577-SNV	rs13219398		A	0.224	A	0.174

2:129452522-SNV	rs6734940		G	0.206	G	0.155
13:61665117-SNV	rs9570456		A	0.193	A	0.143
22:29166461-SNV	rs80086076		G	0.064	G	0.013
1:17628832-SNV	rs112597506		G	0.105	G	0.054
12:57789359-SNV	rs68147365	R3HDM2	A	0.137	A	0.086
12:94156150-SNV	rs7962567	CRADD	T	0.068	T	0.017
13:63583989-SNV	rs7999289		T	0.065	T	0.014
3:134743312-SNV	rs9843311	EPHB1	A	0.144	A	0.093
10:9598942-SNV	rs12777981		G	0.167	G	0.116
2:207058522-SNV	rs12478085	GPR1	C	0.160	C	0.109
10:20847349-SNV	rs572323260		A	0.325	A	0.274
8:102673483-SNV	rs4339613	GRHL2	C	0.113	C	0.062
7:81403969-SNV	rs35642091		T	0.104	T	0.053
6:128194473-SNV	rs12194062	THEMIS	T	0.200	T	0.148
18:47831904-SNV	rs74957066		C	0.051		0.000
13:99331612-SNV	rs9554494		A	0.258	A	0.207
2:103572929-SNV	rs2310351		G	0.208	G	0.156
4:172005983-SNV	rs13133786		G	0.192	G	0.141
7:73309777-SNV	rs75383256		T	0.234	T	0.182
21:41155357-SNV	rs116996665	IGSF5	G	0.136	G	0.084
3:167688697-SNV	rs13081021		T	0.215	T	0.163
1:188626294-SNV	rs10798236		T	0.370	T	0.318

1:82747972-SNV	rs12060896		A	0.447	A	0.394
1:213815045-SNV	rs58204067		T	0.086	T	0.034
9:19756526-SNV	rs10811237	SLC24A2	A	0.336	A	0.284
10:82201116-SNV	rs4387297		T	0.166	T	0.113
8:22238888-SNV	rs6990847	SLC39A14	G	0.056	G	0.003
11:101915848-SNV	rs112804364		T	0.076	T	0.023
9:13813046-SNV	rs7869330		G	0.254	G	0.201
11:26598844-SNV	rs293984	ANO3	T	0.169	T	0.116
4:63296955-SNV	rs1528375		T	0.129	T	0.075
9:15000126-SNV	rs7029010	LOC389705	C	0.407	C	0.354
11:5773051-SNV	rs1498557		A	0.281	A	0.227
6:153227783-SNV	rs34097134		T	0.209	T	0.155
13:110021208-SNV	rs35146697		A	0.162	A	0.108
18:2508148-SNV	rs59464959		C	0.405	C	0.352
4:65402099-SNV	rs1017276		T	0.434	T	0.380
5:148825837-SNV	rs353247		T	0.475	T	0.422
14:86670937-SNV	rs1959751		T	0.077	T	0.023
1:101207899-SNV	rs79284256		A	0.112	A	0.058
3:36576628-SNV	rs7627024	STAC	C	0.176	C	0.122
8:11218648-SNV	rs75923184	TDH	G	0.164	G	0.109
12:96184417-SNV	rs180702233	NTN4	T	0.119	T	0.065
7:81458916-SNV	rs35275069		A	0.160	A	0.106

3:106446085-SNV	rs4536860		C	0.221	C	0.167
4:155395680-SNV	rs72725350	DCHS2	C	0.370	C	0.316
3:72633303-SNV	rs6549471		T	0.254	T	0.200
12:16547410-SNV	rs11056918		T	0.441	T	0.387
6:95130171-SNV	rs9363200		C	0.249	C	0.194
5:4661512-SNV	rs12513900		C	0.272	C	0.217
5:177739628-SNV	rs2913774	COL23A1	C	0.386	C	0.331
7:5191334-SNV	rs10251398		C	0.256	C	0.201
18:74929214-SNV	rs7234261		A	0.228	A	0.173
2:239447550-SNV	rs11887099	LINC01107	C	0.201	C	0.145
6:129969911-SNV	rs12661601	ARHGAP18	C	0.449	C	0.393
17:13500604-SNV	rs726800	HS3ST3A1	G	0.101	G	0.045
1:170917638-SNV	rs1033481	MROH9	A	0.338	A	0.283
2:106980294-SNV	rs10180742		A	0.209	A	0.153
1:222048876-SNV	rs4846357		C	0.440	C	0.384
7:24245418-SNV	rs62451496		G	0.213	G	0.157
3:15204856-SNV	rs1287457		T	0.163	T	0.106
19:38584785-SNV	rs12971962	SIPA1L3	G	0.312	G	0.255
5:80619949-SNV	rs138571846		A	0.059	A	0.002
9:139035609-SNV	rs11103350		G	0.429	G	0.373
14:104072793-SNV	rs4525427		A	0.220	A	0.164
17:9874387-SNV	rs16959128	GAS7	G	0.113	G	0.057

15:63777614-SNV	rs4984291		A	0.278	A	0.221
1:230421319-SNV	rs75434142		G	0.139	G	0.082
17:6094133-SNV	rs7406684		A	0.259	A	0.203
3:30652788-SNV	rs6550006	TGFBR2	G	0.206	G	0.149
10:29203987-SNV	rs703031		T	0.197	T	0.140
20:53866731-SNV	rs6023980		G	0.090	G	0.033
9:29717566-SNV	rs7860347		T	0.131	T	0.074
13:63616358-SNV	rs17090495		T	0.077	T	0.020
16:2340603-SNV	rs161424	ABCA3	G	0.103	G	0.046
15:70121894-SNV	rs17676727		G	0.290	G	0.233
1:242202601-SNV	rs4658581		C	0.171	C	0.113
3:55184534-SNV	rs13069457		C	0.200	C	0.142
2:239901835-SNV	rs10210888		T	0.172	T	0.114
9:98518654-SNV	rs62558997		C	0.207	C	0.149
18:9970076-SNV	rs29186		T	0.132	T	0.074
18:13186765-SNV	rs12953436		C	0.490	C	0.432
3:58332912-SNV	rs4681681	PXK	T	0.313	T	0.255
10:58623046-SNV	rs12255115		A	0.098	A	0.040
10:122010902-SNV	rs12218469		A	0.174	A	0.116
1:5536722-SNV	rs12023817		A	0.228	A	0.170
4:184251673-SNV	rs6552658		C	0.263	C	0.205
9:87450224-SNV	rs12057045	NTRK2	T	0.257	T	0.199

12:49273215-SNV	rs146437037		T	0.150	T	0.092
13:113280906-SNV	rs6577099		G	0.337	G	0.279
8:127495576-SNV	rs6998726		G	0.325	G	0.266
13:114809157-SNV	rs61973889	RASA3	A	0.224	A	0.165
6:162664803-SNV	rs80346353	PRKN	A	0.254	A	0.195
16:57574961-SNV	rs73545075		A	0.118	A	0.059
18:707560-SNV	rs2612106	ENOSF1	A	0.473	A	0.414
1:22943799-SNV	rs66653724		T	0.218	T	0.158
7:128727855-SNV	rs13242417		A	0.123	A	0.064
9:139443847-SNV	rs2282183	NALT1	C	0.415	C	0.355
4:57035112-SNV	rs984672		A	0.361	A	0.301
10:109316960-SNV	rs61866367		T	0.349	T	0.289
10:81422066-SNV	rs2559764	LINC02679	C	0.222	C	0.162
18:14715920-SNV	rs467052		C	0.257	C	0.197
12:78218954-SNV	rs34216258		G	0.213	G	0.153
7:153011134-SNV	rs4725495		G	0.251	G	0.191
4:48420410-SNV	rs73246023	SLAIN2	C	0.286	C	0.226
13:83786771-SNV	rs1606406		A	0.156	A	0.096
11:47280762-SNV	rs11039155	NR1H3	A	0.199	A	0.138
11:72463435-SNV	rs7109575	ARAP1	A	0.192	A	0.132
4:15381482-SNV	rs62289262	C1QTNF7,C1QTNF7-AS1	A	0.142	A	0.082

1:181872787-SNV	rs11577986		C	0.181	C	0.120
14:59525598-SNV	rs137994406		A	0.346	A	0.285
11:130436641-SNV	rs11608220		T	0.251	T	0.190
8:123883273-SNV	rs11786424	ZHX2	C	0.327	C	0.266
13:45389004-SNV	rs965086		T	0.397	T	0.336
8:9818872-SNV	rs1973560		A	0.164	A	0.102
18:34657148-SNV	rs145193602	KIAA1328	T	0.287	T	0.226
22:33872468-SNV	rs2032475	LARGE1	G	0.279	G	0.217
10:29250426-SNV	rs2453618		A	0.166	A	0.104
5:3697286-SNV	rs6555218		C	0.309	C	0.247
4:63254161-SNV	rs10029837		C	0.341	C	0.279
11:119841515-SNV	rs656382		T	0.296	T	0.234
9:116708896-SNV	rs11788091	ZNF618	G	0.116	G	0.054
10:59256573-SNV	rs11005819		T	0.436	T	0.374
19:6173146-SNV	rs972856	ACSBG2	A	0.323	A	0.261
11:1916653-SNV	rs528626		A	0.333	A	0.271
9:123351121-SNV	rs7861226		A	0.245	A	0.182
4:8475226-SNV	rs11737745	TRMT44	T	0.170	T	0.107
10:107354505-SNV	rs10884190		G	0.396	G	0.333
2:177507262-SNV	rs2710028	LINC01117	A	0.473	A	0.410
5:155610046-SNV	rs1835920		C	0.376	C	0.312
6:10441311-SNV	rs303068	MIR5689HG	A	0.367	A	0.304

3:68708559-SNV	rs1553485		C	0.228	C	0.165
20:23000009-SNV	rs6113897		A	0.454	A	0.390
4:32392708-SNV	rs73117718		G	0.068	G	0.004
10:36699564-SNV	rs2384601		T	0.309	T	0.245
14:99178143-SNV	rs7161699	C14orf177	A	0.360	A	0.296
9:22800940-SNV	rs73653809	LINC01239	C	0.066	C	0.002
6:135395548-SNV	rs13220662		A	0.307	A	0.242
1:3657759-SNV	rs12117836	TP73-AS1	A	0.450	A	0.385
10:10040163-SNV	rs71485659		A	0.134	A	0.069
3:36139631-SNV	rs1450014		T	0.156	T	0.092
8:134196849-SNV	rs4527850		C	0.280	C	0.215
10:9447147-SNV	rs12779342		T	0.142	T	0.077
2:48591607-SNV	rs7559431	FOXN2	A	0.224	A	0.159
17:66702344-SNV	rs872387		C	0.300	C	0.235
6:170468361-SNV	rs3012384		T	0.415	T	0.349
2:124012480-SNV	rs13003552		T	0.210	T	0.144
15:33438052-SNV	rs2468758	FMN1	G	0.286	G	0.220
2:77049269-SNV	rs4852419	LRRTM4	A	0.400	A	0.335
9:7451850-SNV	rs2997557		G	0.438	G	0.373
4:43792147-SNV	rs1713525		G	0.467	G	0.401
5:163341866-SNV	rs35588991		T	0.439	T	0.373
9:99055991-SNV	rs13284535	HSD17B3	A	0.186	A	0.120

14:81410653-SNV	rs67231034		T	0.474	T	0.408
21:39681036-SNV	rs2211865		C	0.338	C	0.273
4:136140611-SNV	rs13121015		G	0.393	G	0.327
7:57171362-SNV	rs6960194		G	0.223	G	0.157
2:239871408-SNV	rs4073004		T	0.179	T	0.113
11:361574-SNV	rs11246120		A	0.173	A	0.106
2:177814839-SNV	rs12613997		T	0.420	T	0.353
19:22114704-SNV	rs17769372		C	0.417	C	0.350
15:78202948-SNV	rs71398986		A	0.348	A	0.281
2:122039514-SNV	rs1975378	TFCP2L1	G	0.345	G	0.278
6:107502748-SNV	rs9480749	PDSS2	G	0.241	G	0.174
11:97140778-SNV	rs61894281		T	0.266	T	0.200
1:38093277-SNV	rs61776211	RSPO1	A	0.242	A	0.175
17:4950175-SNV	rs147044784		T	0.103	T	0.036
20:7288268-SNV	rs2224050	LINC01751	C	0.220	C	0.153
18:28147942-SNV	rs3095761		C	0.300	C	0.233
7:129416820-SNV	rs57560217		C	0.399	C	0.332
15:60286020-SNV	rs57809386		C	0.312	C	0.244
20:51282552-SNV	rs6063858		G	0.476	G	0.408
7:157070800-SNV	rs28709519		T	0.296	T	0.228
8:113114520-SNV	rs9297463		C	0.398	C	0.330
14:90883818-SNV	rs759061		A	0.471	A	0.403

15:98115496-SNV	rs12594126		A	0.418	A	0.349
1:28466221-SNV	rs12065041		A	0.203	A	0.134
4:9886860-SNV	rs10939599	SLC2A9	A	0.263	A	0.195
10:124089514-SNV	rs9663185	BTBD16	A	0.469	A	0.401
1:221676494-SNV	rs1342090		C	0.403	C	0.335
16:80133691-SNV	rs2126997		G	0.337	G	0.269
17:79585611-SNV	rs147114956	NPLOC4	A	0.404	A	0.335
6:145335208-SNV	rs4368827		C	0.433	C	0.364
22:17394989-SNV	rs1860945		C	0.303	C	0.234
13:100040979-SNV	rs7992229		A	0.424	A	0.355
10:4778393-SNV	rs11252692		T	0.370	T	0.301
2:16937318-SNV	rs798369		G	0.478	G	0.409
10:36467829-SNV	rs55650985		G	0.122	G	0.053
20:18958105-SNV	rs2224614		A	0.363	A	0.293
6:133021084-SNV	rs3798794	VNN1	C	0.357	C	0.287
8:8089335-SNV	rs60998183	FAM86B3P	G	0.313	G	0.243
12:97501280-SNV	rs61941672		A	0.283	A	0.213
3:62399917-SNV	rs56389750	CADPS	T	0.403	T	0.333
3:65184395-SNV	rs577772449		T	0.189	T	0.119
6:153566247-SNV	rs9479570		T	0.459	T	0.388
13:98431518-SNV	rs9554421		C	0.376	C	0.304
8:55120970-SNV	rs170165		C	0.436	C	0.365

4:139017219-SNV	rs1975373	LINC00616,SLC7A11-AS1	G	0.462	G	0.391
11:117283808-SNV	rs9163	CEP164	T	0.270	T	0.198
1:190603308-SNV	rs35089183	LINC01720	A	0.393	A	0.321
1:196984679-SNV	rs1332666		G	0.469	G	0.398
2:57853921-SNV	rs2169168		T	0.384	T	0.312
9:88977839-SNV	rs12003569		G	0.229	G	0.158
7:89056432-SNV	rs9649127		A	0.391	A	0.319
5:66961928-SNV	rs4615263		C	0.407	C	0.335
13:68154660-SNV	rs7332419		C	0.392	C	0.320
22:18994209-SNV	rs7286229	DGCR5	G	0.108	G	0.036
2:61973870-SNV	rs6718032		T	0.460	T	0.387
9:76321504-SNV	rs11143667		G	0.327	G	0.255
9:120390282-SNV	rs1887904		A	0.409	A	0.337
15:35453290-SNV	rs4924581		A	0.420	A	0.347
22:49408394-SNV	rs738609		G	0.420	G	0.347
14:52274858-SNV	rs56329637		T	0.401	T	0.328
8:114698755-SNV	rs75699914		G	0.206	G	0.133
18:18712214-SNV	rs111667085		G	0.456	G	0.383
18:1818382-SNV	rs4525568		G	0.485	G	0.411
3:195506473-SNV	rs201922637	MUC4	G	0.276	G	0.203
16:5806640-SNV	rs7201928		C	0.437	C	0.364

10:1736305-SNV	rs7923745	ADARB2	T	0.264	T	0.191
3:149166067-SNV	rs7631053		C	0.414	C	0.340
1:108650760-SNV	rs12140469		C	0.186	C	0.113
22:45455489-SNV	rs35006348		G	0.259	G	0.186
1:80631231-SNV	rs4330881		A	0.424	A	0.350
6:95126381-SNV	rs9345481		A	0.405	A	0.332
13:37136557-SNV	rs9315430		C	0.488	C	0.414
9:123349511-SNV	rs10984962		G	0.352	G	0.278
7:27866358-SNV	rs2051830	TAX1BP1	G	0.321	G	0.247
7:25936614-SNV	rs12536040		A	0.315	A	0.241
20:184956-SNV	rs6109479		C	0.203	C	0.128
6:147344448-SNV	rs1997340	STXBP5-AS1	T	0.131	T	0.056
8:5818671-SNV	rs7015774		C	0.462	C	0.387
8:80745551-SNV	rs10109063	LOC101927040	T	0.453	T	0.377
12:55268160-SNV	rs11171137		G	0.308	G	0.232
19:28490151-SNV	rs12974171	LOC101927151	G	0.466	G	0.391
7:47571488-SNV	rs334516	TNS3	A	0.378	A	0.302
12:130757395-SNV	rs10848060		C	0.435	C	0.359
7:17815402-SNV	rs2723512		T	0.297	T	0.221
11:3371184-SNV	rs12785333		T	0.396	T	0.320
15:62483875-SNV	rs56252148		T	0.225	T	0.149
5:64526677-SNV	rs62369578	ADAMTS6	G	0.463	G	0.387

4:104949606-SNV	rs11097839		G	0.258	G	0.182
16:61123708-SNV	rs1812551		C	0.341	C	0.264
13:42099080-SNV	rs61962983		T	0.399	T	0.322
4:53023881-SNV	rs13116983		G	0.460	G	0.383
5:66996304-SNV	rs4976023		G	0.460	G	0.383
10:111523759-SNV	rs2488930		C	0.409	C	0.332
6:107151522-SNV	rs910528		A	0.455	A	0.377
6:32443223-SNV	rs9269108		A	0.300	A	0.223
9:101008541-SNV	rs4743193	TBC1D2	T	0.489	T	0.411
8:92531120-SNV	rs2657197		T	0.477	T	0.399
6:43826627-SNV	rs943080		C	0.408	C	0.330
2:49417863-SNV	rs11685850		T	0.384	T	0.305
21:47797806-SNV	rs73907471	PCNT	C	0.274	C	0.195
2:112056562-SNV	rs1519518	MIR4435-2HG	T	0.290	T	0.211
5:66834961-SNV	rs2888227		C	0.436	C	0.357
4:105132790-SNV	rs7668819		A	0.448	A	0.368
2:139873568-SNV	rs905995		T	0.236	T	0.156
3:65207266-SNV	rs9827817		C	0.309	C	0.229
8:1146075-SNV	rs7009856	DLGAP2	C	0.407	C	0.328
2:181185422-SNV	rs1850676		A	0.348	A	0.268
4:136241123-SNV	rs1580246		G	0.494	A	0.414
14:36654831-SNV	rs113809595		C	0.416	C	0.335

18:41976886-SNV	rs1456601	LINC01478	G	0.451	G	0.370
15:26615762-SNV	rs4906874		G	0.220	G	0.140
5:66822058-SNV	rs113021423		T	0.259	T	0.179
3:2090801-SNV	rs2729028		G	0.290	G	0.209
2:55427470-SNV	rs6545479	CLHC1	C	0.195	C	0.114
16:66159066-SNV	rs1308841		A	0.485	A	0.403
9:78488545-SNV	rs7866631		G	0.314	G	0.232
12:2116369-SNV	rs2159095		A	0.414	A	0.332
7:57016137-SNV	rs62461011		T	0.278	T	0.196
1:168285844-SNV	rs2268568		T	0.360	T	0.278
12:20918765-SNV	rs12818167		T	0.498	C	0.415
5:49881858-SNV	rs11744698		G	0.473	G	0.391
6:32423355-SNV	rs9268804		G	0.499	C	0.416
2:121910733-SNV	rs2677536		C	0.451	C	0.367
12:24965640-SNV	rs11047651	BCAT1	C	0.432	C	0.349
12:46472606-SNV	rs6582609		T	0.414	T	0.330
7:16761125-SNV	rs7793943		G	0.401	G	0.318
6:169196350-SNV	rs56756239		A	0.491	A	0.407
10:98695375-SNV	rs10882857	LCOR	G	0.454	G	0.370
8:56323434-SNV	rs2622537	XKR4	A	0.406	A	0.322
3:194663479-SNV	rs4476454		C	0.470	C	0.385
7:25889153-SNV	rs12700666		A	0.463	A	0.377

14:24214218-SNV	rs8011524		T	0.470	T	0.384
6:115170399-SNV	rs12190938		G	0.491	G	0.405
21:15988057-SNV	rs1380450	LOC388813	G	0.491	G	0.405
15:60283211-SNV	rs8025697		A	0.481	A	0.395
4:8512250-SNV	rs3103098		G	0.473	G	0.387
1:73664563-SNV	rs7522520		T	0.493	T	0.406
11:114200602-SNV	rs60237448		A	0.480	A	0.394
5:161265211-SNV	rs79118357		C	0.204	C	0.116
12:17019550-SNV	rs12230060		A	0.475	A	0.387
18:14721886-SNV	rs56000333		G	0.370	G	0.281
18:4675411-SNV	rs7231733		C	0.496	C	0.406
2:133148988-SNV	rs72997289		C	0.383	C	0.293
22:44928102-SNV	rs5765762		T	0.429	T	0.339
6:97440350-SNV	rs761129	KLHL32	C	0.361	C	0.270
6:115977588-SNV	rs9400866		C	0.486	C	0.394
5:169534259-SNV	rs4315935	FOXI1	A	0.404	A	0.313
11:105391884-SNV	rs10895830		A	0.471	A	0.380
11:2259087-SNV	rs4506651		G	0.478	G	0.385
13:69826493-SNV	rs1336182	LINC00383	G	0.473	G	0.379
14:52797860-SNV	rs12147805		G	0.423	G	0.328
4:72385194-SNV	rs74909297	SLC4A4	G	0.246	G	0.151
12:76237736-SNV	rs12819031		C	0.165	C	0.070

18:21210377-SNV	rs12457684	ANKRD29	A	0.491	A	0.396
4:60531469-SNV	rs11725848		T	0.479	T	0.384
9:137927576-SNV	rs7864039		A	0.463	A	0.366
13:81112717-SNV	rs4360823		A	0.181	A	0.084
13:90659602-SNV	rs17664303		A	0.426	A	0.328
5:61766977-SNV	rs35120	IPO11	A	0.477	A	0.380
16:5843139-SNV	rs56142463		T	0.371	T	0.273
8:91656967-SNV	rs6471243	TMEM64	C	0.452	C	0.353
6:115575266-SNV	rs141896008		G	0.316	G	0.217
9:106926693-SNV	rs1889285		G	0.466	G	0.367
2:133173065-SNV	rs7592134		G	0.419	G	0.320
2:241364613-SNV	rs73119527		C	0.121	C	0.021
16:64075157-SNV	rs36025325		T	0.448	T	0.345
13:97057492-SNV	rs7995859	HS6ST3	A	0.489	A	0.387
19:9635882-SNV	rs9305091	ZNF426	A	0.392	A	0.290
7:57020930-SNV	rs1402889		A	0.451	A	0.348
21:37437566-SNV	rs62229260		G	0.455	G	0.350
11:97079764-SNV	rs7119782		C	0.492	C	0.386
8:62156656-SNV	rs2931357		C	0.493	C	0.386
11:63130311-SNV	rs12283500		G	0.496	A	0.385
2:169782481-SNV	rs484066	ABCB11	A	0.386	A	0.273
3:114822794-SNV	rs12171287	ZBTB20	T	0.336	T	0.487

1:190993925-SNV	rs6660833		G	0.164	G	0.311
5:45879434-SNV	rs6414908		G	0.319	G	0.446
4:149442440-SNV	rs59109853		C	0.369	C	0.485
5:73377132-SNV	rs10036719		G	0.385	G	0.500
13:81067147-SNV	rs7322230		A	0.331	A	0.443
4:133172326-SNV	rs13115227		T	0.176	T	0.286
1:155082298-SNV	rs7548955		T	0.354	T	0.462
11:55198664-SNV	rs4090717		A	0.153	A	0.261
4:136305642-SNV	rs6818696		A	0.352	A	0.459
8:125414083-SNV	rs4457313		T	0.330	T	0.436
1:71748282-SNV	rs12737582		G	0.373	G	0.478
6:64260279-SNV	rs10943869		C	0.247	C	0.352
16:78094058-SNV	rs7195021		C	0.338	C	0.443
21:23670078-SNV	rs2827367		A	0.305	A	0.408
1:95396932-SNV	rs767015	CNN3-DT	T	0.319	T	0.422
4:144244716-SNV	rs300899		G	0.369	G	0.471
13:44988662-SNV	rs9533844		T	0.367	T	0.469
2:229349458-SNV	rs11890080	LINC01807	C	0.300	C	0.401
12:92807007-SNV	rs10859314		T	0.377	T	0.477
11:121527052-SNV	rs519185		C	0.308	C	0.408
21:33782652-SNV	rs2833800		T	0.368	T	0.469
9:106917770-SNV	rs10991125		G	0.284	G	0.384

5:83633197-SNV	rs12697733	EDIL3	C	0.378	C	0.477
11:14606058-SNV	rs2278805	PSMA1	G	0.324	G	0.423
2:49384560-SNV	rs4971679		A	0.262	A	0.361
4:186152733-SNV	rs3108274	SNX25	T	0.399	T	0.498
20:47224787-SNV	rs4325821		T	0.310	T	0.408
1:184085324-SNV	rs12409700		A	0.291	A	0.389
14:55158424-SNV	rs2236296	SAMD4A	G	0.320	G	0.417
1:16505320-SNV	rs1497406		G	0.356	G	0.453
17:4951663-SNV	rs34548580		G	0.379	G	0.475
2:103571066-SNV	rs2310350		G	0.322	G	0.418
4:109795293-SNV	rs62314575	COL25A1	A	0.372	A	0.468
6:32509919-SNV	rs72853970		A	0.343	A	0.438
11:4273104-SNV	rs2923794		C	0.144	C	0.239
4:162887162-SNV	rs17640064	FSTL5	C	0.385	C	0.480
6:151729053-SNV	rs7775083	RMND1	C	0.331	C	0.426
9:31937788-SNV	rs4395978		T	0.142	T	0.236
5:49886200-SNV	rs60226866		A	0.075	A	0.168
17:66860583-SNV	rs16973376		T	0.386	T	0.479
4:133080569-SNV	rs13137993		G	0.241	G	0.334
18:6900243-SNV	rs4797264	ARHGAP28	G	0.301	G	0.394
20:55595500-SNV	rs1276372		A	0.262	A	0.355
14:74094475-SNV	rs11621630		T	0.366	T	0.458

1:147369425-SNV	rs7512708		C	0.227	C	0.319
3:155949082-SNV	rs7373565	KCNAB1	G	0.338	G	0.430
13:113328201-SNV	rs4907739	ATP11AUN	C	0.311	C	0.402
3:53522314-SNV	rs1264098		G	0.225	G	0.316
8:84566741-SNV	rs1157610		G	0.371	G	0.462
4:186072110-SNV	rs11729626	SLC25A4	A	0.382	A	0.472
16:53498655-SNV	rs8054299	RBL2	G	0.191	G	0.281
6:48746893-SNV	rs2490133		A	0.394	A	0.484
14:36590826-SNV	rs201236909	LINC00609	A	0.319	A	0.408
17:48793230-SNV	rs4794179		T	0.395	T	0.484
3:173245378-SNV	rs35788354	NLGN1	G	0.370	G	0.459
18:6469841-SNV	rs9964334		A	0.384	A	0.472
4:190194335-SNV	rs62342929		C	0.316	C	0.404
14:51201652-SNV	rs7154318	NIN	T	0.272	T	0.359
1:70258866-SNV	rs1361494	LRRC7	T	0.342	T	0.429
7:20614895-SNV	rs16872575		T	0.166	T	0.253
1:191010239-SNV	rs12025411		A	0.248	A	0.335
14:88529291-SNV	rs12884100	LINC01146	C	0.385	C	0.472
15:89553293-SNV	rs997831		T	0.155	T	0.242
14:39567494-SNV	rs8011075	SEC23A	A	0.399	A	0.486
17:12865263-SNV	rs12103542	ARHGAP44	G	0.309	G	0.395
7:73241386-SNV	rs7455225		T	0.338	T	0.425

6:136021092-SNV	rs55789102	LINC00271	T	0.364	T	0.450
6:24942222-SNV	rs414243	RIPOR2	T	0.197	T	0.283
10:113022313-SNV	rs4287275		C	0.145	C	0.231
4:8518470-SNV	rs139005026			0.000	C	0.085
11:4263923-SNV	rs61898885		T	0.184	T	0.269
11:97137260-SNV	rs4754927		T	0.381	T	0.466
5:77386900-SNV	rs2545424	AP3B1	A	0.148	A	0.233
14:41073476-SNV	rs61986792		A	0.279	A	0.363
4:83415849-SNV	rs4544702	TMEM150C	A	0.350	A	0.434
2:133141152-SNV	rs12052350		A	0.328	A	0.412
22:19060412-SNV	rs7286581	DGCR2	A	0.281	A	0.365
18:13565750-SNV	rs1785163	LDLRAD4	T	0.224	T	0.308
4:44570965-SNV	rs16857122		C	0.193	C	0.277
13:90663132-SNV	rs2794492		G	0.308	G	0.391
12:111715197-SNV	rs1265565	CUX2	T	0.101	T	0.184
14:52245887-SNV	rs8006707		A	0.392	A	0.475
6:135352648-SNV	rs9321481	HBS1L	G	0.396	G	0.479
16:59615655-SNV	rs12709032		T	0.251	T	0.334
13:34012002-SNV	rs1198628	STARD13	C	0.385	C	0.467
4:57623051-SNV	rs59964404		G	0.369	G	0.452
7:56442594-SNV	rs79524533		C	0.281	C	0.363
16:62562592-SNV	rs12447892		C	0.345	C	0.427

10:91416084-SNV	rs6586213		A	0.228	A	0.311
14:81420774-SNV	rs2371462		T	0.324	T	0.406
22:49825954-SNV	rs7288342		A	0.318	A	0.400
12:58522096-SNV	rs34586018		A	0.267	A	0.349
16:75539518-SNV	rs34852438		A	0.154	A	0.236
3:5725927-SNV	rs62245849		G	0.174	G	0.255
3:155348417-SNV	rs10936013	PLCH1	A	0.215	A	0.297
6:14542733-SNV	rs1014964		T	0.381	T	0.462
11:117410682-SNV	rs2510853	DSCAML1	G	0.356	G	0.437
12:125648922-SNV	rs10846837		T	0.347	T	0.428
7:56308138-SNV	rs62460103		T	0.388	T	0.469
15:81339871-SNV	rs1320322		A	0.087	A	0.168
21:25412481-SNV	rs8134281		A	0.190	A	0.271
1:43496093-SNV	rs61775700		A	0.251	A	0.332
4:88458825-SNV	rs4693836		A	0.368	A	0.449
4:141829615-SNV	rs1364887	RNF150	A	0.302	A	0.383
2:216975588-SNV	rs13002401	XRCC5	G	0.170	G	0.250
8:62185084-SNV	rs62524885		C	0.210	C	0.290
7:125434707-SNV	rs1362040		A	0.385	A	0.464
13:112862732-SNV	rs1888250		T	0.294	T	0.373
8:11496207-SNV	rs6601600		C	0.308	C	0.387
2:55395807-SNV	rs6545475		T	0.342	T	0.420

12:45733422-SNV	rs12579771	ANO6	T	0.367	T	0.446
8:13475278-SNV	rs12155866		A	0.311	A	0.389
3:23017533-SNV	rs7432693		G	0.248	G	0.326
4:39002185-SNV	rs11727608	TMEM156	A	0.120	A	0.198
7:142429237-SNV	rs10238724		G	0.399	G	0.477
11:60860889-SNV	rs440879	CD5	A	0.352	A	0.429
5:180104905-SNV	rs6601131		T	0.337	T	0.415
3:145837379-SNV	rs10935602	PLOD2	A	0.206	A	0.283
8:62133820-SNV	rs2978540		T	0.172	T	0.248
3:100452573-SNV	rs4928085	TFG	C	0.309	C	0.385
22:44928733-SNV	rs12484057		A	0.085	A	0.161
18:21315697-SNV	rs12970254	LAMA3	G	0.346	G	0.422
20:58727296-SNV	rs8116055	MIR646HG	G	0.309	G	0.384
18:63252877-SNV	rs12964418		G	0.223	G	0.299
4:138916879-SNV	rs11931168		G	0.404	G	0.480
7:133395492-SNV	rs2016876	EXOC4	G	0.256	G	0.332
12:99492650-SNV	rs74388702	ANKS1B	C	0.038	C	0.113
20:184221-SNV	rs6041402		G	0.276	G	0.351
18:659958-SNV	rs2847613	TYMS	C	0.394	C	0.469
3:192760805-SNV	rs2886923		A	0.337	A	0.412
19:19279437-SNV	rs12974661	BORCS8-MEF2B,MEF2B	G	0.230	G	0.305

3:189309808-SNV	rs9864888		T	0.342	T	0.416
10:42646082-SNV	rs28408671		T	0.085	T	0.160
2:228912194-SNV	rs4294998	SPHKAP	C	0.266	C	0.341
19:19248107-SNV	rs2040562	TMEM161A	C	0.343	C	0.417
12:5265091-SNV	rs1351265		C	0.210	C	0.284
11:22435202-SNV	rs2593670		C	0.267	C	0.342
10:116512624-SNV	rs808302	ABLIM1	C	0.164	C	0.238
2:30075307-SNV	rs62129766	ALK	T	0.316	T	0.390
2:216872462-SNV	rs71351629	MREG	A	0.338	A	0.412
10:105052976-SNV	rs4293063		G	0.412	G	0.485
12:82600267-SNV	rs12831678		A	0.355	A	0.429
1:217747965-SNV	rs10863324	GPATCH2	C	0.409	C	0.483
2:181288322-SNV	rs1349720		C	0.400	C	0.474
3:88891337-SNV	rs2046326		C	0.191	C	0.265
15:51652661-SNV	rs4775940	GLDN	G	0.271	G	0.344
21:37130888-SNV	rs7277012		A	0.059	A	0.132
6:29874910-SNV	rs9259616		T	0.322	T	0.395
11:2024451-SNV	rs2525886		T	0.195	T	0.269
6:166186011-SNV	rs7748044		A	0.320	A	0.393
7:101438978-SNV	rs12535452		A	0.105	A	0.178
4:105796781-SNV	rs6842065		C	0.365	C	0.438
17:28841318-SNV	rs4794874	GOSR1	C	0.176	C	0.248

11:97916052-SNV	rs2508525		A	0.173	A	0.245
20:38248603-SNV	rs6065145		A	0.269	A	0.341
10:79297978-SNV	rs56816832	KCNMA1	G	0.142	G	0.214
10:128770620-SNV	rs61875502	DOCK1	A	0.224	A	0.297
4:52971878-SNV	rs34565134		C	0.291	C	0.363
10:5971452-SNV	rs12774886	FBH1	A	0.120	A	0.193
3:34530889-SNV	rs7624958		C	0.263	C	0.335
17:17255640-SNV	rs12602578		T	0.280	T	0.352
14:56514050-SNV	rs12587734		G	0.312	G	0.384
1:47424594-SNV	rs4660983		G	0.223	G	0.295
20:21813336-SNV	rs11087364		T	0.344	T	0.415
5:24961311-SNV	rs112372608		C	0.310	C	0.381
14:52790922-SNV	rs59623061	PTGER2	T	0.067	T	0.138
18:76390238-SNV	rs1975336		T	0.396	T	0.467
3:36179510-SNV	rs10222618		C	0.349	C	0.420
12:71897924-SNV	rs6582037	LGR5	G	0.191	G	0.262
17:32256079-SNV	rs2881844	ASIC2	C	0.271	C	0.342
12:56564811-SNV	rs7960225	SMARCC2	A	0.237	A	0.307
18:72848494-SNV	rs1542068		C	0.148	C	0.219
3:116665321-SNV	rs73147831		A	0.200	A	0.270
4:180418475-SNV	rs2254490		T	0.187	T	0.258
5:29814433-SNV	rs1456644		C	0.381	C	0.451

9:1382843-SNV	rs2183843		A	0.327	A	0.398
7:129407615-SNV	rs6965780		C	0.400	C	0.470
17:20944549-SNV	rs62058993	USP22	C	0.180	C	0.250
18:6476724-SNV	rs7241002		A	0.155	A	0.225
8:134301771-SNV	rs2930005	NDRG1	A	0.298	A	0.368
8:124732424-SNV	rs4595110	ANXA13	G	0.397	G	0.467
10:114791490-SNV	rs12260037	TCF7L2	T	0.261	T	0.331
18:74299131-SNV	rs55662509		A	0.369	A	0.439
19:42278288-SNV	rs6508997		T	0.364	T	0.433
14:98758033-SNV	rs55762616		G	0.263	G	0.332
1:159748886-SNV	rs3806185		T	0.248	T	0.317
4:23761719-SNV	rs13435356		T	0.368	T	0.437
5:9154659-SNV	rs1806151	SEMA5A	C	0.316	C	0.384
9:20532434-SNV	rs10964597	MLLT3	T	0.148	T	0.217
12:9044157-SNV	ss1388088831		A	0.396	A	0.464
11:1679232-SNV	rs11038542		T	0.390	T	0.458
7:16978430-SNV	rs4538783		A	0.140	A	0.208
13:110596927-SNV	rs4773104		T	0.222	T	0.290
3:62941112-SNV	rs2367425		T	0.289	T	0.357
4:138596210-SNV	rs150529846		T	0.163	T	0.231
13:103987550-SNV	rs4772549		T	0.194	T	0.262
19:21024556-SNV	rs2547173		A	0.188	A	0.256

2:1101708-SNV	rs140174155	SNTG2	T	0.312	T	0.380
11:55229763-SNV	rs76243490		C	0.084	C	0.152
5:172352600-SNV	rs4868230	ERGIC1	A	0.192	A	0.260
20:18076982-SNV	rs1819356		T	0.338	T	0.406
4:79248194-SNV	rs391641	FRAS1	A	0.044	A	0.112
7:127284269-SNV	rs35297615		A	0.168	A	0.235
5:105335580-SNV	rs12517228		A	0.285	A	0.352
13:64807499-SNV	rs9571147		C	0.399	C	0.467
8:118907971-SNV	rs7841914	EXT1	A	0.393	A	0.460
6:68024858-SNV	rs9363740		T	0.349	T	0.416
20:55236558-SNV	rs6014787		G	0.194	G	0.262
6:48755527-SNV	rs2490136		G	0.264	G	0.332
18:31316708-SNV	rs4392137	ASXL3	C	0.344	C	0.411
4:142817111-SNV	rs12504128		G	0.337	G	0.405
5:92485295-SNV	rs7718530		A	0.249	A	0.316
12:41375828-SNV	rs712133	CNTN1	T	0.398	T	0.465
13:114010154-SNV	rs2295960	GRTP1,GRTP1-AS1	A	0.345	A	0.411
5:105498693-SNV	rs11747060		A	0.320	A	0.387
13:55173063-SNV	rs7999254		C	0.152	C	0.219
9:54021-SNV	rs79371206		C	0.149	C	0.216
3:148362574-SNV	rs35251486		C	0.083	C	0.150
7:113116798-SNV	rs34498116		T	0.156	T	0.223

2:186756214-SNV	rs12616304		C	0.068	C	0.134
20:56299718-SNV	rs55720455		A	0.173	A	0.239
14:21500455-SNV	rs3818615	NDRG2,TPPP2	A	0.208	A	0.274
17:71836616-SNV	rs2620046		G	0.202	G	0.268
22:47919132-SNV	rs1297381		C	0.221	C	0.287
15:68676110-SNV	rs11637375	ITGA11	A	0.174	A	0.240
11:9737755-SNV	rs56235379	SWAP70	A	0.171	A	0.237
3:1803562-SNV	rs12486838		T	0.291	T	0.357
15:33632639-SNV	rs8042460	RYR3	T	0.247	T	0.313
12:1160615-SNV	rs12423543	ERC1	A	0.402	A	0.468
15:28264501-SNV	rs62007485	OCA2	T	0.346	T	0.411
15:65191448-SNV	rs1684053		T	0.169	T	0.234
7:17640425-SNV	rs182202591		C	0.001	C	0.067
13:99889529-SNV	rs76081348	UBAC2	T	0.124	T	0.190
10:18660892-SNV	rs201072938	CACNB2	A	0.132	A	0.197
3:70351567-SNV	rs67418210	MDFIC2	T	0.084	T	0.150
16:1536499-SNV	rs2745097	PTX4	T	0.167	T	0.232
12:81432884-SNV	rs6539544		A	0.201	A	0.266
6:36955064-SNV	rs1554721	MTCH1	A	0.226	A	0.291
4:14117951-SNV	rs11944910	LINC01085	G	0.167	G	0.231
1:224819839-SNV	rs12077707	CNIH3	C	0.199	C	0.263
8:15125504-SNV	rs78122452		A	0.069	A	0.134

2:37992910-SNV	rs12471513		T	0.389	T	0.453
22:50496179-SNV	rs73183363	TTLL8	C	0.180	C	0.245
19:5359314-SNV	rs2082464		A	0.342	A	0.406
11:4268837-SNV	rs35721509		T	0.092	T	0.156
12:50361100-SNV	rs296762		A	0.320	A	0.384
21:27051285-SNV	rs3787616	JAM2	T	0.257	T	0.321
12:132349736-SNV	rs4964941		A	0.131	A	0.195
8:31774687-SNV	rs4263721	NRG1	C	0.286	C	0.350
16:10731554-SNV	rs2430645	TEKT5	G	0.065	G	0.129
3:32264058-SNV	rs4370060		T	0.391	T	0.455
2:227166119-SNV	rs10201609		A	0.244	A	0.307
3:55177886-SNV	rs1688334		C	0.361	C	0.425
16:25560440-SNV	rs711200		T	0.152	T	0.216
3:58429908-SNV	rs11925533		A	0.228	A	0.292
19:49465960-SNV	rs918546	BAX	T	0.422	T	0.485
6:9048796-SNV	rs73720945		A	0.052	A	0.116
15:90286894-SNV	rs72754536	WDR93	T	0.202	T	0.265
16:20441215-SNV	rs8062563	ACSM5	G	0.212	G	0.275
8:134068748-SNV	rs2702967	PTCSC1,SLA,TG	A	0.408	A	0.471
2:36409250-SNV	rs148627697		C	0.090	C	0.152
6:45852249-SNV	rs12214996		T	0.295	T	0.358
5:126156222-SNV	rs62391741	LMNB1	C	0.374	C	0.437

2:61859248-SNV	rs12472568		T	0.327	T	0.390
10:43449749-SNV	rs2506031		A	0.118	A	0.181
8:16058995-SNV	rs1484754		C	0.198	C	0.260
2:124763222-SNV	rs55767348		A	0.188	A	0.251
1:86616137-SNV	rs529321527	COL24A1	C	0.014	C	0.077
9:120410454-SNV	rs4240445	LOC101928797	G	0.138	G	0.200
7:5186487-SNV	rs57342527		T	0.195	T	0.258
19:52106238-SNV	rs2864139		A	0.250	A	0.312
9:136113834-SNV	rs4357365		C	0.326	C	0.388
17:12976937-SNV	rs11659037		G	0.225	G	0.287
2:79278036-SNV	rs1522860		G	0.096	G	0.158
3:42766284-SNV	rs59478814	CCDC13	G	0.216	G	0.278
13:22428118-SNV	rs1570731		C	0.239	C	0.300
18:14670335-SNV	rs62082703		C	0.233	C	0.294
5:117184769-SNV	rs73258914	LINC02147	C	0.056	C	0.117
2:43125754-SNV	rs4953680		G	0.429	G	0.491
3:196515151-SNV	rs6773348	PAK2	A	0.084	A	0.146
4:161831811-SNV	rs4383576		A	0.202	A	0.263
3:94196764-SNV	rs9840682		G	0.095	G	0.156
9:26275493-SNV	rs693087		G	0.250	G	0.311
5:165033915-SNV	rs1592767		A	0.067	A	0.128
6:30486679-SNV	rs2534803	LINC02569	T	0.140	T	0.201

7:25410265-SNV	rs73691564		C	0.001	C	0.062
3:120002295-SNV	rs1849933		A	0.124	A	0.186
5:120299204-SNV	rs12520288		C	0.077	C	0.138
11:101676886-SNV	rs3920965		T	0.409	T	0.470
16:20663135-SNV	rs151310	ACSM1	G	0.356	G	0.417
8:102685133-SNV	rs12547409		C	0.226	C	0.287
6:53437710-SNV	rs13210774	LOC101927136	T	0.240	T	0.300
8:68918088-SNV	rs4737252	PREX2	C	0.265	C	0.326
20:56783204-SNV	rs62204343		T	0.127	T	0.187
12:104709864-SNV	rs7958103	TXNRD1	G	0.079	G	0.140
4:186073859-SNV	rs36005131		G	0.244	G	0.304
3:185536223-SNV	rs6444082	IGF2BP2	T	0.298	T	0.359
10:59184403-SNV	rs11005795		G	0.213	G	0.273
11:25927674-SNV	rs56098435		G	0.163	G	0.223
16:20607747-SNV	rs145821719		A	0.167	A	0.227
8:15854002-SNV	rs78822488		T	0.145	T	0.205
1:19014865-SNV	rs7527312	PAX7	G	0.142	G	0.202
12:69345715-SNV	rs12817847	CPM	G	0.224	G	0.284
7:111960866-SNV	rs12113149	ZNF277	G	0.342	G	0.401
6:127477441-SNV	rs1936808	RSPO3	C	0.032	C	0.092
8:90823817-SNV	rs392827		C	0.220	C	0.280
1:163847340-SNV	rs17374983		T	0.167	T	0.226

14:28522253-SNV	rs2775285		A	0.417	A	0.476
1:227815948-SNV	rs10799430	ZNF678	T	0.246	T	0.305
22:45264599-SNV	rs2349861		T	0.227	T	0.286
12:46348859-SNV	rs17096361	SCAF11	C	0.285	C	0.344
15:59544159-SNV	rs7180541	MYO1E	T	0.263	T	0.322
6:131622476-SNV	rs2104208		T	0.159	T	0.218
14:88363469-SNV	rs2401727		G	0.397	G	0.456
10:43337851-SNV	rs72782057		A	0.097	A	0.155
4:157580626-SNV	rs75954206		A	0.180	A	0.238
6:30826904-SNV	rs2535327		C	0.215	C	0.273
4:184428347-SNV	rs4302494	ING2	A	0.123	A	0.182
13:51282422-SNV	rs797509		G	0.155	G	0.214
10:4518720-SNV	rs11252555		G	0.146	G	0.204
7:57972572-SNV	rs8185472		A	0.051	A	0.109
2:113880947-SNV	rs315936	IL1RN	T	0.257	T	0.315
4:161419315-SNV	rs13143739		A	0.030	A	0.088
7:25919337-SNV	rs4722533		C	0.280	C	0.338
9:1383919-SNV	rs998573		G	0.176	G	0.234
7:48903385-SNV	rs10234909		C	0.281	C	0.339
4:88374763-SNV	rs148332014	NUDT9	T	0.022	T	0.079
7:29783027-SNV	rs62458277	DPY19L2P3	A	0.418	A	0.475
14:52881618-SNV	rs1902864		A	0.114	A	0.172

13:70920352-SNV	rs9572436		A	0.146	A	0.203
12:30347152-SNV	rs7971144		C	0.245	C	0.302
1:222364313-SNV	rs35325312		T	0.351	T	0.408
2:148270253-SNV	rs6755641		A	0.402	A	0.459
20:4614201-SNV	rs73601218		C	0.064	C	0.120
15:51909555-SNV	rs12708427	DMXL2	C	0.140	C	0.196
18:13185383-SNV	rs66718605		T	0.090	T	0.146
18:39537513-SNV	rs73454598	PIK3C3	G	0.045	G	0.102
12:115088985-SNV	rs7486620		G	0.086	G	0.143
15:67002309-SNV	rs62005625	SMAD6	G	0.147	G	0.203
17:53374198-SNV	rs73990640	HLF	T	0.087	T	0.144
17:79604264-SNV	rs62074750	TSPAN10	A	0.075	A	0.131
8:13469748-SNV	rs71522362		G	0.183	G	0.239
11:46346150-SNV	rs61882672		A	0.136	A	0.192
10:76861920-SNV	rs10824273	DUSP13	T	0.120	T	0.176
3:112901722-SNV	rs73229016		G	0.076	G	0.132
10:27155643-SNV	rs12414672		C	0.117	C	0.173
19:54187161-SNV	rs28730960		C	0.105	C	0.161
5:77218488-SNV	rs6453360	LOC101929154	C	0.240	C	0.295
2:170639989-SNV	rs4668164		G	0.060	G	0.115
2:127881367-SNV	rs11674775		T	0.064	T	0.119
2:164630062-SNV	rs4667702		C	0.099	C	0.154

8:72313853-SNV	rs60177768	EYA1	C	0.137	C	0.192
12:101565791-SNV	rs73156677	SLC5A8	T	0.039	T	0.094
9:27409288-SNV	rs10812587	MOB3B	A	0.362	A	0.417
9:78497748-SNV	rs4483223		C	0.150	C	0.205
1:4592382-SNV	rs1515673		A	0.267	A	0.322
12:9086898-SNV	ss1388090056	PHC1	G	0.386	G	0.440
5:80262946-SNV	rs35766440	RASGRF2	A	0.241	A	0.295
3:65004205-SNV	rs17072075		C	0.143	C	0.197
14:32776302-SNV	rs8005191		T	0.124	T	0.179
18:11675940-SNV	rs35150497		T	0.379	T	0.432
13:106675179-SNV	rs7986962		C	0.252	C	0.306
19:56340369-SNV	rs7248235	NLRP11	T	0.290	T	0.344
1:219787413-SNV	rs10779364	ZC3H11B	G	0.357	G	0.411
9:15045554-SNV	rs57114160		T	0.148	T	0.202
2:161827204-SNV	rs1722656		G	0.177	G	0.231
19:17863321-SNV	rs12978944	FCHO1	G	0.064	G	0.117
19:31129469-SNV	rs7359965	ZNF536	A	0.020	A	0.073
6:116974423-SNV	rs9320575	ZUP1	A	0.381	A	0.434
16:50340027-SNV	rs56342387	ADCY7	A	0.106	A	0.159
12:42697155-SNV	rs78720876			0.000	T	0.053
5:108611792-SNV	rs10075278	LOC285638	A	0.067	A	0.120
3:30592802-SNV	rs79697821		T	0.093	T	0.145

12:92863382-SNV	rs74480110	LINC02397	G	0.086	G	0.139
1:32347703-SNV	rs610798		C	0.255	C	0.307
14:31673994-SNV	rs59933112	HECTD1	C	0.167	C	0.219
15:42168400-SNV	rs2290559	SPTBN5	T	0.224	T	0.276
2:9617951-SNV	rs4545978	IAH1	A	0.057	A	0.109
11:6624181-SNV	rs4758438	RRP8	C	0.120	C	0.172
6:54651629-SNV	rs62412581		G	0.143	G	0.195
7:8169009-SNV	rs539800133	ICA1	T	0.093	T	0.144
3:132551940-SNV	rs112549627	NPHP3-AS1	G	0.083	G	0.135
6:54711100-SNV	rs10428844	FAM83B	T	0.239	T	0.290
6:94560440-SNV	rs9354053		T	0.258	T	0.310
10:16152340-SNV	rs60713519		A	0.078	A	0.130
8:41580906-SNV	rs2304874	ANK1	A	0.221	A	0.273
16:87942200-SNV	rs12050979	CA5A	C	0.133	C	0.184
1:147223200-SNV	rs1891499		T	0.044	T	0.095
3:17065116-SNV	rs6789316	PLCL2	T	0.162	T	0.213
13:74388061-SNV	rs74095758	KLF12	T	0.048	T	0.099
13:71873267-SNV	rs2325358		C	0.172	C	0.223
2:181783595-SNV	rs12619976		T	0.419	T	0.470
4:181595575-SNV	rs965376		C	0.374	C	0.425
21:40280034-SNV	rs60559455	LOC400867	A	0.039	A	0.090
20:56041510-SNV	rs6070114		A	0.283	A	0.334

9:116483862-SNV	rs60220613		G	0.049	G	0.100
1:82169124-SNV	rs66932869	ADGRL2	T	0.116	T	0.167
10:84559237-SNV	rs1813838	NRG3	A	0.107	A	0.158
4:120546659-SNV	rs4833622	PDE5A	G	0.162	G	0.212
7:101452693-SNV	rs12668982		C	0.048	C	0.099
2:103575834-SNV	rs9677209		T	0.080	T	0.130
16:86707142-SNV	rs377145		A	0.356	A	0.406
1:53582306-SNV	rs3766785	SLC1A7	T	0.100	T	0.150
12:108155353-SNV	rs73189581	PRDM4	A	0.049	A	0.099
12:53104942-SNV	rs938177		T	0.078	T	0.128
4:55709941-SNV	rs716918		G	0.195	G	0.245
4:120579973-SNV	rs7676589		C	0.066	C	0.116
11:118500963-SNV	rs680465	PHLDB1	A	0.127	A	0.176
8:65472343-SNV	rs78873350		A	0.165	A	0.214
18:73031809-SNV	rs7242281		A	0.026	A	0.075
2:198658661-SNV	rs75521970		A	0.067	A	0.116
10:79410291-SNV	rs145832559		A	0.016	A	0.066
19:29031439-SNV	rs6508934	LOC100420587	T	0.140	T	0.189
