# Supplementary material for: Identification of lipid quantitative trait loci linked with cardiometabolic disease in Asian Indians and Europeans: A genome-wide association study and Mendelian randomization
Source: PLoS Med. 2026 Apr 23;23(4):e1005039. doi: 10.1371/journal.pmed.1005039 (PMC13105358; doi:10.1371/journal.pmed.1005039)
Supplement: S3 File — (PDF) [file pmed.1005039.s029.pdf]

Supplementary Table 1. Analysis pipeline and quality control steps in the construction of PRS using European, South Asian, and Ancestry-specific PRS models in the training set

EU_PRS	SA_PRS	AI_PRS
113,320 Variants	6,630,150 variants	46,985,978 variants
LD Pruning- 67,500 variants	LD Pruning- 1,231,725 variants	Training set Regression adjusted for age, gender, BMI, 5PCs
Regression adjusted for age, gender, BMI, 5PCs	Regression adjusted for age, gender, BMI, 5PCs	P<0.05 = 742,199 variants
P<0.05 = 1,820 variants	P<0.05 = 53,384 variants	LD Pruning – 199,259 variants
QC (MAF>0.01 to MAF≤0.5) and Removing INS, DEL, MIX SNPs	QC (MAF>0.01 to MAF<0.45) and Removing INS, DEL, MIX SNPs	QC (MAF>0.01 to MAF<0.45) and Removing INS, DEL, MIX SNPs
$p<10^{-3}$ 405 variants	$p<10^{-4}$ 918 variants	$p<10^{-4}$ 925 variants
Rare variants= 150<br>Common variants= 255	Rare variants=499<br>Common variants=408	Rare variants=525<br>Common variants=400

AI- Asian Indians; BMI- Body mass index; DEL- Deletions; EU- Europeans; INS- Insertions; LD- Linkage Disequilibrium; MAF- Minor allele frequency; PCs- Principal components; PRS: Polygenic risk score; SA- South Asians; SNPs- Single nucleotide polymorphisms QC- Quality control

Supplementary Table 2. CAD Cases and Controls in each Training set, Test set and UKBB Validation set categorized by Quartiles

Cohort		Q1		Q2		Q3		Q4	
		CASE	CONTROL	CASE	CONTROL	CASE	CONTROL	CASE	CONTROL
Training Set (N =1616)	EU_PRS	0	404	1	403	2	402	175	227
	SA_PRS	1	403	3	401	0	404	174	228
	AI_PRS	2	402	1	403	0	404	175	227
	EU+AI_PRS	2	402	1	403	0	404	175	227
Test Set (N=2986)	EU_PRS	29	718	88	658	132	614	364	382
	SA_PRS	12	734	87	659	79	667	435	312
	AI_PRS	11	736	79	667	76	670	447	301
	EU+AI_PRS	5	741	60	686	66	680	482	265
UKBB Validation Set (N=9372)	EU_PRS	510	1833	670	1673	799	1544	962	1381
	SA_PRS	415	1928	628	1715	812	1531	1086	1257
	AI_PRS	394	1949	638	1705	825	1518	1084	1259
	EU+AI_PRS	373	1970	590	1753	834	1509	1144	1199

Q1- 1stquartile; Q2- 2nd quartile; Q3- 3rd quartile; Q4- 4th quartile

Supplementary Table 3. Association of extreme PRS Quartiles (Q4 vs. Q1) with CAD using European, South Asian and Ancestry-specific GWAS variants

Cohort	EU_PRS* (Q4 vs. Q1)	SA_PRS* (Q4 vs. Q1)	AI_PRS* (Q4 vs. Q1)	EU+AI_PRS* (Q4 vs. Q1)
**Test Set**<br>**OR (95% CI)**<br>**p-value**	30.81 (20.26-46.84)<br>$7.6x10^{-58}$	143.18 (75.79-270.51)<br>$8.5x10^{-53}$	202.76 (103.44-397.51)<br>$5.8x10^{-54}$	622.20 (224.92-1721.25)<br>$2.9x10^{-35}$
**UKBB Validation Set**<br>**OR (95% CI)**<br>**p-value**	3.15 (2.73-3.64)<br>$2.9x10^{-55}$	5.97 (5.11-6.97)<br>$1.3x10^{-112}$	6.51 (5.56-7.63)<br>$1.7x10^{-119}$	8.27 (7.02-9.73)<br>$1.8x10^{-72}$

*Model adjusted for covariates (Age, Gender, BMI, and T2D)
AI: Asian Indians; CI: Confidence interval; EU: Europeans; OR: Odds ratio; PRS: Polygenic risk score; SA: South Asians; Q1: 1st quartile; Q4: 4th quartile

Supplementary Table 4. Predictive efficacy of polygenic risk score (PRS) for CAD adjusted for medications

Cohort	EU_PRS*<br>OR (95% CI)<br>p-value	SA_PRS*<br>OR (95% CI)<br>p-value	AI_PRS*<br>OR (95% CI)<br>p-value	EU+AI_PRS*<br>OR (95% CI)<br>p-value
**Test Set (N=2986)**	1.79 (1.74-1.85)<br>$7.9x10^{-101}$	1.93 (1.84-2.03)<br>$7.0X10^{-42}$	1.97 (1.88-2.06)<br>$2.9x10^{-49}$	2.08 (1.97-2.19)<br>$5.1x10^{-40}$
**UKBB Validation Set (N=9372)**	1.22 (1.19-1.25)<br>$8.6x10^{-61}$	1.34 (1.29-1.39)<br>$3.0X10^{-127}$	1.35 (1.31-1.39)<br>$4.2x10^{-138}$	1.40 (1.35-1.46)<br>$3.6x10^{-175}$

*Model adjusted for covariates (Age, Gender, BMI, T2D, and Medications)
AI: Asian Indians; CI: Confidence interval; EU: Europeans; OR: Odds ratio; PRS: Polygenic risk score; SA: South Asians; *p values were very high and exact numbers could not be computed with SVS or R

Supplementary Table 5a. Polygenic risk score (PRS) predicts CAD risk independent of T2D.

Cohort	EU_PRS<br>OR (95% CI)<br>p-value			SA_PRS<br>OR (95% CI)<br>p-value			AI_PRS<br>OR (95% CI)<br>p-value			EU+AI_PRS<br>OR (95% CI)<br>p-value		
	**Model 1**	**Model 2 Adjusting for SGCG locus**	**Model 3 Non-Diabetics only**	**Model 1**	**Model 2 Adjusting for SGCG locus**	**Model 3 Non-Diabetics only**	**Model 1**	**Model 2 Adjusting for SGCG locus**	**Model 3 Non-Diabetics only**	**Model 1**	**Model 2 Adjusting for SGCG locus**	**Model 3 Non-Diabetics only**
**Training Set (N=1616)**	1.94 (1.76-2.12) $6.3x10^{-104}$	2.04 (0.51-3.57) $7.9x10^{-229}$	**1.98 (-0.65-4.63) $4.3x10^{-98}$**	2.43 (2.17-2.68) $1.1x10^{-52}$	2.46 (0.29-4.63) <0.000*	**2.46 (0.29-4.63) $8.3x10^{-231}$**	2.47 (2.24-2.70) $1.3x10^{-64}$	2.48 (0.39-4.57) <0.000*	**2.47 (-0.71-5.65) $4.0x10^{-240}$**	2.49 (2.26-2.73) $1.7x10^{-77}$	2.50 (-0.21-5.21) <0.000*	**2.48 (-1.85-6.81) $5.5x10^{-244}$**
**Test Set (N=2986)**	1.48 (1.33-1.63) $6.5x10^{-7}$	1.77 (1.72-1.82) $2.70x10^{-116}$	**1.66 (1.55-1.77) $5.7x10^{-19}$**	1.50 (1.34-1.65) $6.0X10^{-7}$	1.95 (1.86-2.03) $1.41x10^{-52}$	**1.83 (1.63-2.02) $2.0X10^{-9}$**	1.60 (1.46-1.73) $7.4x10^{-12}$	2.0 (1.92-2.08) $5.4x10^{-65}$	**1.79 (1.61-1.97) 2.1x10-10**	1.72 (1.57-1.87) $1.6x10^{-12}$	2.10 (2.00-2.19) $7.8X10^{-51}$	**1.91 (1.69-2.13) $7.6x10^{-9}$**
**UKBB Validation Set (N=9372)**	1.13 (1.07-1.20) $1.1x10^{-18}$	-	**1.20 (1.17-1.23) $2.8x10^{-50}$**	1.13 (1.05-1.21) $4.5X10^{-13}$	-	**1.32 (1.27-1.38) $1.1X10^{-114}$**	1.23 (1.16-1.29) $4.1x10^{-46}$	-	**1.35 (1.29-1.39) $6.8x10^{-131}$**	1.24 (1.17-1.30) $2.0x10^{-47}$	-	**1.38 (1.32-1.44) $6.7x10^{-154}$**

Model 1 adjusted for covariates (Age, Gender, BMI, and T2D)
Model 2 adjusted for covariates (Age, Gender, BMI, T2D, T2D SGCG locus- rs9552911)
Model 3 adjusted for covariates (Age, Gender, BMI) in Non-Diabetics
AI: Asian Indians; CI: Confidence interval; EU: Europeans; OR: Odds ratio; PRS: Polygenic risk score; SA: South Asians
*p values were very high and exact numbers could not be computed with SVS or R.

Supplementary Table 5b. Receiver characteristic curve analysis for CAD PRS derived using European, South Asian, and Ancestry-specific GWAS variants in non-diabetics

Cohort	Test set (N = 1235)		UKBB Validation Set (N = 7429)	
	**Unadjusted**	**Adjusted**	**Unadjusted**	**Adjusted**
**EU_PRS**	0.78 (0.75-0.81)<br>$9.7x10^{-40}$	0.84 (0.81-0.86)<br>$1.3x10^{-55}$	0.59 (0.58-0.61)<br>$5.8x10^{-34}$	0.72 (0.70-0.73)<br>$3.4x10^{-177}$
**SA_PRS**	0.86 (0.84-0.89)<br>$4.0x10^{-64}$	0.90 (0.88-0.92)<br>$1.5x10^{-79}$	0.63 (0.62-0.65)<br>$9.2x10^{-66}$	0.73 (0.72-0.75)<br>$1.2x10^{-202}$
**AI_PRS**	0.88 (0.86-0.90)<br>$2.9x10^{-70}$	0.92 (0.90-0.93)<br>$1.5x10^{-84}$	0.61 (0.60-0.62)<br>$4.2x10^{-46}$	0.72 (0.71-0.74)<br>$2.8x10^{-185}$
**AI+EU_PRS**	0.90 (0.88-0.92)<br>$1.6x10^{-77}$	0.93 (0.91-0.95)<br>$1.1x10^{-89}$	0.64 (0.63-0.66)<br>$1.7x10^{-76}$	0.74 (0.73-0.75)<br>$3.9x10^{-214}$

Adjusted for age, gender, BMI

Supplementary Table 6. The predictive efficacy of polygenic risk score (PRS) for CAD remains strong even after using stringent QC protocols

Cohort	EU_PRS*$ OR (95% CI) p-value	EU_PRS*# OR (95% CI) p-value	SA_PRS*$ OR (95% CI) p-value	SA_PRS*# OR (95% CI) p-value	AI_PRS*$ OR (95% CI) p-value	AI_PRS*# OR (95% CI) p-value	EU+AI_PRS*$ OR (95% CI) p-value	EU+AI_PRS*# OR (95% CI) p-value
**Training Set (N=1616)**	1.79 (1.62-1.97) $2.7x10^{-77}$	1.94 (1.76-2.12) $6.3x10^{-104}$	2.49 (2.29-2.69) $3.01x10^{-59}$	2.43 (2.17-2.68) $1.1x10^{-52}$	2.44 (2.21-2.66) $2.7x10^{-63}$	2.47 (2.24-2.70) $1.3x10^{-64}$	2.34 (2.08-2.60) $1.81x10^{-62}$	2.49 (2.26-2.73) $1.7x10^{-77}$
**Test Set (N=2986)**	1.36 (1.18-1.53) $7.1x10^{-04}$	1.48 (1.33-1.63) $6.5x10^{-7}$	1.50 (1.33-1.65) $8.4X10^{-07}$	1.50 (1.34-1.65) $6.0X10^{-7}$	1.45 (1.32-1.58) $1.7x10^{-08}$	1.60 (1.46-1.73) $7.4x10^{-12}$	1.60 (1.54-1.65) $5.5x10^{-10}$	1.72 (1.57-1.87) $1.6x10^{-12}$
**UKBB Validation Set (N=9372)**	1.10 (1.02-1.17) $1.0x10^{-08}$	1.13 (1.07-1.20) $1.1x10^{-18}$	1.13 (1.03-1.22) $2.4X10^{-09}$	1.13 (1.05-1.21) $4.5X10^{-13}$	1.15 (1.10-1.21) $9.0x10^{-26}$	1.23 (1.16-1.29) $4.1x10^{-46}$	1.17 (1.11-1.23) $1.3x10^{-30}$	1.24 (1.17-1.30) $2.0x10^{-47}$

*$Model adjusted for covariates (Age, Gender, BMI, and T2D) using PRS QC methods by Choi et al., 2020 (Nature Protocols)

***#** Model adjusted for covariates (Age, Gender, BMI, and T2D) using PRS QC detailed in the manuscript and Supplementary Table 1.

AI: Asian Indians; CI: Confidence interval; EU: Europeans; OR: Odds ratio; PRS: Polygenic risk score; SA: South Asian

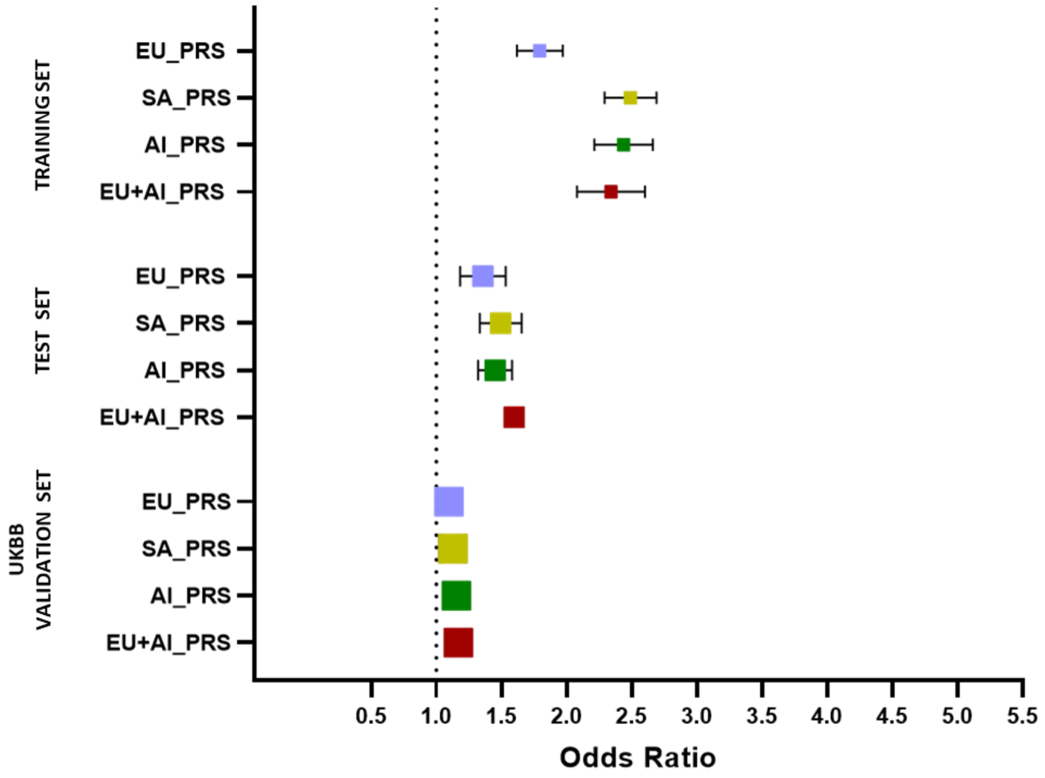

OR (95% CI)	P-value
1.79 (1.62-1.97)	$2.7\text{x}10^{-77}$
2.49 (2.29-2.69)	$3.01\text{x}10^{-59}$
2.44 (2.21-2.66)	$2.7\text{x}10^{-63}$
2.34 (2.08-2.60)	$1.81\text{x}10^{-62}$
1.36 (1.18-1.53)	$7.1\text{x}10^{-04}$
1.50 (1.33-1.65)	$8.4\text{x}10^{-07}$
1.45 (1.32-1.58)	$1.7\text{x}10^{-08}$
1.60 (1.54-1.65)	$5.5\text{x}10^{-10}$
1.10 (1.02-1.17)	$1.0\text{x}10^{-08}$
1.13 (1.03-1.22)	$2.4\text{x}10^{-09}$
1.15 (1.10-1.21)	$9.0\text{x}10^{-26}$
1.17 (1.11-1.23)	$1.3\text{x}10^{-30}$

Supplementary Figure 1. Forest plot showing effect sizes and confidence interval for CAD risk using European (EU_PRS), South Asians (SA_PRS), Asian Indian (AI_PRS), and combined European and Asian Indians (EU+AI_PRS). Following PRS QC guidelines by Choi *et al*. (2020), each PRS was constructed using a Training set (AIDHS/SDS) (n=1616) and then validated on the Test Set (independent additional samples from AIDHS/SDS) (n=2986) and UKBB Validation set (n=9372) comprising South Asian Indians.

AIDHS/SDS: Asian Indian Diabetic Heart Study/Sikh Diabetes Study; CI: Confidence interval; OR: Odds Ratio, PRS: Polygenic risk score; UKBB: UK Biobank
