# Supplementary material for: Identification of lipid quantitative trait loci linked with cardiometabolic disease in Asian Indians and Europeans: A genome-wide association study and Mendelian randomization
Source: PLoS Med. 2026 Apr 23;23(4):e1005039. doi: 10.1371/journal.pmed.1005039 (PMC13105358; doi:10.1371/journal.pmed.1005039)
Supplement: S1 STROBE-MR Checklist — (DOCX) [file pmed.1005039.s030.docx]

**STROBE-MR checklist of recommended items to address in reports of Mendelian randomization studies**^1^ ^2^

| **Item No.** | **Section** | **Checklist item** | **Page No.** | **Relevant text from manuscript** |
| --- | --- | --- | --- | --- |
| 1 | **TITLE and ABSTRACT** | Indicate Mendelian randomization (MR) as the study’s design in the title and/or the abstract if that is a main purpose of the study | Title section, pg 1 | Identification of lipid quantitative trait loci linked with cardiometabolic disease in Asian Indians and Europeans: a genome-wide association study and Mendelian randomization |
|  | **INTRODUCTION** |  |  |  |
| 2 | **Background** | Explain the scientific background and rationale for the reported study. What is the exposure? Is a potential causal relationship between exposure and outcome plausible? Justify why MR is a helpful method to address the study question | Introduction section, paragraph 2 and 3, lines 198-212 | South Asians have a higher incidence of T2D and are significantly more susceptible to CVD. Genome-wide association studies (GWAS) of blood lipids have identified genetic associations shared between lipid subclasses and various diseases; however, the large bulk of the information from such studies is derived from European (EU) populations. In this study, we have for the first time performed a metabolite-GWAS of blood lipid phenotypes associated with T2D and CV traits in a Punjabi population originating from Northern India. |
| 3 | **Objectives** | State specific objectives clearly, including pre-specified causal hypotheses (if any). State that MR is a method that, under specific assumptions, intends to estimate causal effects | Introduction section paragraph 3, lines 204-212 | The objective was to identify genetic associations of the known metabolites, identify ancestry-specific differences, and determine shared genetic associations between metabolites and cardiometabolic disease phenotypes to determine causal relationships using two-sample Mendelian randomization (MR) and therapeutic targets. |
|  | **METHODS** |  |  |  |
| 4 | **Study design and data sources** | Present key elements of the study design early in the article. Consider including a table listing sources of data for all phases of the study. For each data source contributing to the analysis, describe the following: |  |  |
|  | a) | Setting: Describe the study design and the underlying population, if possible. Describe the setting, locations, and relevant dates, including periods of recruitment, exposure, follow-up, and data collection, when available. | Methodology section, lines 217-472 | The methodology includes the study cohort with the study design.  The study cohort with recruitment site, follow-up, and data collection is mentioned under study cohorts. |
|  | b) | Participants: Give the eligibility criteria, and the sources and methods of selection of participants. Report the sample size, and whether any power or sample size calculations were carried out prior to the main analysis | Methodology section, paragraph 1, line 217 | A total of 3000 individuals (1725 T2D cases and 1275 controls) were included in this study. No power calculation was performed. |
|  | c) | Describe measurement, quality control and selection of genetic variants | Methodology section, paragraph 4-6, lines 261-312  Methodology section, paragraph 7, lines 315-335 | Lipidomics data measurement was performed using LC-MS. Its QC and selection of lipids are mentioned in Methodology.  Genotyping, imputation, QC, and selection of variants are clearly mentioned in the Methodology. |
|  | d) | For each exposure, outcome, and other relevant variables, describe methods of assessment and diagnostic criteria for diseases | Methodology section, paragraph 8, lines 337-359  Methodology section, lines 217-472 | The statistical analysis section includes diagnoses, predictors, and outcomes.  The methodology section explains sources of data and the detailed methodology of measurement and estimation. |
|  | e) | Provide details of ethics committee approval and participant informed consent, if relevant | Methodology section, paragraph 1, lines 235-244 | All participants in this study were recruited following the written informed consent procedures approved by the institutional review boards (IRBs). All AIDHS/SDS protocols and consent documents were reviewed and approved by the University of Oklahoma Health Science Center’s IRB, as well as the Human Subject Protection (Ethics) Committees at the participating hospitals and institutes in India. All human studies reported in this manuscript abide by the Declaration of Helsinki principles. |
| 5 | **Assumptions** | Explicitly state the three core IV assumptions for the main analysis (relevance, independence, and exclusion restriction) as well as assumptions for any additional or sensitivity analysis | Methodology section, paragraph 14, lines 422-443 | Three basic hypotheses were considered while conducting two-sample MR: (1) genetic instrument variables (IVs) should be robustly associated with the exposure; (2) IVs should not be directly correlated to the outcome and affect the outcome merely via the exposure without any gene pleiotropy; and (3) IVs should be independent of any potential confounders. |
| 6 | **Statistical methods: main analysis** | Describe statistical methods and statistics used |  |  |
|  | a) | Describe how quantitative variables were handled in the analyses (i.e., scale, units, model) | Methodology section, paragraph 8, lines 337-359 | Statistical analysis subsections depict how quantitative variables were handled. |
|  | b) | Describe how genetic variants were handled in the analyses and, if applicable, how their weights were selected | Methodology section, paragraph 14, lines 422-443 | We ensured that the genetic instrument was strongly associated with the exposure in the target (ancestry similar or non-similar) population based on regression (beta) coefficients and P value/F statistics accounting for the LD and allele frequency. Based on the differences in LD and MAF, the MR sensitivity analysis selected and excluded the variants from each ancestry to ensure data harmonization a reduce pleiotropy. |
|  | c) | Describe the MR estimator (e.g. two-stage least squares, Wald ratio) and related statistics. Detail the included covariates and, in case of two-sample MR, whether the same covariate set was used for adjustment in the two samples | Methodology section, paragraph 14, lines 422-443 | The statistical analysis subsections mention the methods used to perform the analysis and the adjustment of confounders.  For the 2-sample MR, the lipid association with the variant was adjusted for age, sex, BMI, genetic PCs, metabolite PCs, and classical lipids. In the case of CAD, it was adjusted for age, sex, BMI, and T2D. For T2D they were adjusted for age, sex, and BMI. |
|  | d) | Explain how missing data were addressed |  |  |
|  | e) | If applicable, indicate how multiple testing was addressed |  |  |
| 7 | **Assessment of assumptions** | Describe any methods or prior knowledge used to assess the assumptions or justify their validity |  | 2-sample MR analysis method proposed by Burgess et al., 2017 was used. |
| 8 | **Sensitivity analyses and additional analyses** | Describe any sensitivity analyses or additional analyses performed (e.g. comparison of effect estimates from different approaches, independent replication, bias analytic techniques, validation of instruments, simulations) | Methodology section, paragraph 14, lines 422-443 | Sensitivity analyses were performed using the MR Egger method of Burgess and Thompson, which is based on the hypothesis that the pleiotropic effects are independently distributed from the genetic associations with the exposure. |
| 9 | **Software and pre-registration** |  |  |  |
|  | a) | Name statistical software and package(s), including version and settings used |  | Two-sample MR package in R version 4.3.3 |
|  | b) | State whether the study protocol and details were pre-registered (as well as when and where) |  |  |
|  | **RESULTS** |  |  |  |
| 10 | **Descriptive data** |  |  |  |
|  | a) | Report the numbers of individuals at each stage of included studies and reasons for exclusion. Consider use of a flow diagram | Methodology section, paragraph 1, lines 226-230 | The study cohort subsection clearly depicts the number of individuals included and excluded. |
|  | b) | Report summary statistics for phenotypic exposure(s), outcome(s), and other relevant variables (e.g. means, SDs, proportions) | Results section, paragraph 6, lines 578-598 | The summary statistics for phenotypic exposure, outcomes were mentioned in Results section under Univariable Mendelian randomization (MR) and sensitivity analysis subsection. |
|  | c) | If the data sources include meta-analyses of previous studies, provide the assessments of heterogeneity across these studies |  |  |
|  | d) | For two-sample MR:  i.  Provide justification of the similarity of the genetic variant-exposure associations between the exposure and outcome samples  ii.  Provide information on the number of individuals who overlap between the exposure and outcome studies |  |  |
| 11 | **Main results** |  |  |  |
|  | a) | Report the associations between genetic variant and exposure, and between genetic variant and outcome, preferably on an interpretable scale | Results section, paragraph 6, lines 578-598 | The MR results are clearly mentioned under Univariable Mendelian randomization (MR) and sensitivity analysis subsection in Results. |
|  | b) | Report MR estimates of the relationship between exposure and outcome, and the measures of uncertainty from the MR analysis, on an interpretable scale, such as odds ratio or relative risk per SD difference | Results section, paragraph 6, lines 590-598 | The odds ratio or relative risk per SD difference is stated under Univariable Mendelian randomization (MR) and sensitivity analysis subsection in Results. |
|  | c) | If relevant, consider translating estimates of relative risk into absolute risk for a meaningful time period |  |  |
|  | d) | Consider plots to visualize results (e.g. forest plot, scatterplot of associations between genetic variants and outcome versus between genetic variants and exposure) |  | MR sensitivity results are depicted as Forest plots in Figure 4. |
| 12 | **Assessment of assumptions** |  |  |  |
|  | a) | Report the assessment of the validity of the assumptions |  |  |
|  | b) | Report any additional statistics (e.g., assessments of heterogeneity across genetic variants, such as *I^2^*, Q statistic or E-value) |  | The assessments of heterogeneity across variants are tabulated and presented as Supplementary Table 10. |
| 13 | **Sensitivity analyses and additional analyses** |  |  |  |
|  | a) | Report any sensitivity analyses to assess the robustness of the main results to violations of the assumptions | Results section, paragraph 6, lines 578-598 | The sensitivity analyses, such as IVW, weighted median, weighted mode, maximum likelihood, and MR-Egger methods for fixed effects (FE) and random effects (RE) are mentioned under Univariable Mendelian randomization (MR) and sensitivity analysis subsection in Results. |
|  | b) | Report results from other sensitivity analyses or additional analyses |  |  |
|  | c) | Report any assessment of direction of causal relationship (e.g., bidirectional MR) |  |  |
|  | d) | When relevant, report and compare with estimates from non-MR analyses |  |  |
|  | e) | Consider additional plots to visualize results (e.g., leave-one-out analyses) |  |  |
|  | **DISCUSSION** |  |  |  |
| 14 | **Key results** | Summarize key results with reference to study objectives | Discussion section, lines 721-909 | The discussion section effectively summarizes the results and discusses them with reference to the study objectives. |
| 15 | **Limitations** | Discuss limitations of the study, taking into account the validity of the IV assumptions, other sources of potential bias, and imprecision. Discuss both direction and magnitude of any potential bias and any efforts to address them | Discussion section, paragraph 9, lines 893-901 | The limitations of the study clearly consider potential bias or imprecision. |
| 16 | **Interpretation** |  |  |  |
|  | a) | Meaning: Give a cautious overall interpretation of results in the context of their limitations and in comparison with other studies | Discussion section, paragraph 10, lines 910-920 | The summary depicts the identification of new lipid mQTLs associated with phospholipid subclasses LPC O-16:0, PC 38:4 (C), and FA 18:0;(2OH), not reported previously. The involvement of the chromosome 11 region representing FADS1/2 and TMEM258 genes for their role in a variety of metabolic and inflammatory diseases and their association with PC 38:4 (C) in this region, which may increase CAD risk in SAs. Using two-sample MR, PRS, and colocalization analysis, we identified and confirmed a causal association between LPC O-16:0 with T2D, which may be triggered by activation of AMPK, insulin, and leptin signalling contributing to metabolic diseases, immune system dysfunction, cancer, and inflammation. |
|  | b) | Mechanism: Discuss underlying biological mechanisms that could drive a potential causal relationship between the investigated exposure and the outcome, and whether the gene-environment equivalence assumption is reasonable. Use causal language carefully, clarifying that IV estimates may provide causal effects only under certain assumptions |  |  |
|  | c) | Clinical relevance: Discuss whether the results have clinical or public policy relevance, and to what extent they inform effect sizes of possible interventions |  |  |
| 17 | **Generalizability** | Discuss the generalizability of the study results (a) to other populations, (b) across other exposure periods/timings, and (c) across other levels of exposure |  |  |
|  | **OTHER INFORMATION** |  |  |  |
| 18 | **Funding** | Describe sources of funding and the role of funders in the present study and, if applicable, sources of funding for the databases and original study or studies on which the present study is based | Funding section, pg1 | This work was funded by the National Institutes of Health (NIH) grants R01DK082766 and R01DK118427 from the National Institute of Diabetes and Digestive and Kidney Diseases (NIDDK).  DKS, MR, and the Asian Indian Diabetic Heart Study/Sikh Diabetes Study (AIDHS/SDS) were in part supported by NIH grants R01DK082766 and R01DK118427, Dr. Geoffrey Altshuler Children's Hospital Foundation Endowment funds, and funding from Presbyterian Health Foundation of Oklahoma.  CEA, in part, was supported by the Oklahoma Shared Clinical and Translational Resources (U54GM104938) with an Institutional Development Award (IDeA) from NIGMS.  HHG was supported in part by NIH grants R01DK099051 and R01DK118427  RG was supported in part by NIH/R01DK118427.  The funders had no role in study design, data collection and analysis, decision to publish, or preparation of the manuscript. |
| 19 | **Data and data sharing** | Provide the data used to perform all analyses or report where and how the data can be accessed, and reference these sources in the article. Provide the statistical code needed to reproduce the results in the article, or report whether the code is publicly accessible and if so, where | Data availability section, lines 939-952 | The genome-wide genotype data associated with the Punjabi Sikh discovery, along with phenotype data, have already been submitted to dbGaP https://www.ncbi.nlm.nih.gov/gap/advanced_search/?TERM=sanghera. The variant data on genome-wide polygenic risk score (PRS) is available through our previous manuscript (Rout et al, Adv Endocrinol Metab. 2023 [PMID: 38152657] and Rout et al, J Cardiovasc Trans Res. 2024 17(5):1086-1096; [PMID: 38658478] ). The data from these studies, which contain sensitive participant information, will be made available for further analysis upon reasonable request through collaborations with the University of Oklahoma Health Sciences Center’s Institutional IRB (irb@ouhsc.edu). |
| 20 | **Conflicts of Interest** | All authors should declare all potential conflicts of interest | Conflict of interest section, lines 923-924 | We declare that no conflict of interest could be perceived as prejudicing the impartiality of the research reported. |

This checklist is copyrighted by the Equator Network under the Creative Commons Attribution 3.0 Unported (CC BY 3.0) license.

1. Skrivankova VW, Richmond RC, Woolf BAR, Yarmolinsky J, Davies NM, Swanson SA, et al. Strengthening the Reporting of Observational Studies in Epidemiology using Mendelian Randomization (STROBE-MR) Statement. JAMA. 2021;under review.

2. Skrivankova VW, Richmond RC, Woolf BAR, Davies NM, Swanson SA, VanderWeele TJ, et al. Strengthening the Reporting of Observational Studies in Epidemiology using Mendelian Randomisation (STROBE-MR): Explanation and Elaboration. BMJ. 2021;375:n2233.
